# A Systematic Review of Long-Interval Intracortical Inhibition as a Biomarker in Neuropsychiatric Disorders

**DOI:** 10.3389/fpsyt.2021.678088

**Published:** 2021-06-02

**Authors:** Parmis Fatih, M. Utku Kucuker, Jennifer L. Vande Voort, Deniz Doruk Camsari, Faranak Farzan, Paul E. Croarkin

**Affiliations:** ^1^Mayo Clinic Department of Psychiatry and Psychology, Mayo Clinic, Rochester, MN, United States; ^2^School of Mechatronic Systems Engineering, Centre for Engineering-Led Brain Research, Simon Fraser University, Surrey, BC, Canada

**Keywords:** cortical inhibition, electroencephalography, electromyography, long-interval intracortical inhibition, transcranial magnetic stimulation

## Abstract

Long-interval intracortical inhibition (LICI) is a paired-pulse transcranial magnetic stimulation (TMS) paradigm mediated in part by gamma-aminobutyric acid receptor B (GABA_B_) inhibition. Prior work has examined LICI as a putative biomarker in an array of neuropsychiatric disorders. This review conducted in accordance with the Preferred Reporting Items for Systematic Reviews and Meta-Analyses (PRISMA) sought to examine existing literature focused on LICI as a biomarker in neuropsychiatric disorders. There were 113 articles that met the inclusion criteria. Existing literature suggests that LICI may have utility as a biomarker of GABA_B_ functioning but more research with increased methodologic rigor is needed. The extant LICI literature has heterogenous methodology and inconsistencies in findings. Existing findings to date are also non-specific to disease. Future research should carefully consider existing methodological weaknesses and implement high-quality test-retest reliability studies.

## Introduction

Gamma-aminobutyric acid (GABA) is the primary inhibitory neurotransmitter of the central nervous system (1). Cortical inhibition is the physiologic mechanism that modulates cortical excitability and neuroplasticity via the suppression created by the GABAergic neurotransmission (2, 3). Prior studies suggest that GABA and cortical inhibition have a role in the pathophysiology of neuropsychiatric disorders. It has been speculated that GABAergic neurotransmission is altered in various brain based disorders such as mood disorders, psychotic disorders, anxiety, attention-deficit/hyperactivity disorder (ADHD), autism spectrum disorder, neurocognitive disorders, epilepsy, movement disorders, and stroke (4–11). A variety of pre-clinical methods have been used to investigate the role of GABA in the pathophysiology of neuropsychiatric disorders. The safe and non-invasive measurement of GABAergic inhibitory neurotransmission is challenging in clinical studies (12–14).

Transcranial magnetic stimulation (TMS) is a non-invasive brain stimulation technique that utilizes magnetic fields to stimulate nerve cells for the treatment of depression and as a neurophysiological probe (15). Single- and paired-pulse TMS paradigms are frequently used to assess cortical inhibitory and excitatory mechanisms (16–18). Prior research indicated that the pairing of a conditioning stimulus with a subsequent test stimulus at varying interstimulus intervals generates either intracortical inhibition or facilitation based on the duration of interstimulus interval and intensity of the conditioning stimulus (19, 20). Facilitation and inhibition is measured by either electromyographic (EMG) recordings of motor-evoked potentials or electroencephalography (EEG) recordings (21). TMS-EMG has been used to measure the cortical inhibition in the motor cortex. Subsequent studies with TMS-EEG have facilitated the study of the dorsolateral pre-frontal cortex which is implicated in the pathophysiology of neuropsychiatric conditions (22, 23).

Long-interval intracortical inhibition (LICI) is a paired-pulse technique with suprathreshold conditioning and test stimuli applied at interstimulus intervals of 50–200 ms leading to suppression of cortical activity. Prior work suggests that the inhibitory effects of LICI are mediated by GABA_B_ receptors (24). The interstimulus interval that produces LICI corresponds to the timing of GABA_B_ inhibitory post-synaptic potentials (25). Furthermore, pharmacological studies demonstrated that GABA_B_ receptor agonist baclofen, GABA uptake inhibitor tiagabine and GABA structural analog vigabatrin potentiate LICI (26–28). Moreover, short-interval intracortical inhibition (SICI), a TMS paired paradigm mediated by GABA_A_ activity, is suppressed by LICI which could be explained with pre-synaptic GABA_B_ mediated inhibition of interneurons (29).

Numerous prior studies have examined cortical excitatory and inhibitory measures using TMS to understand the underlying pathophysiology of neuropsychiatric disorders. Studies of LICI suggest that GABA_B_ mediated inhibition is altered in various brain based conditions such as mood disorders and psychotic disorders, epilepsy, Parkinson's disease, traumatic brain injury, and dementia. In clinical practice and research, psychiatric disorders are diagnosed based on a checklist approach to symptoms with either Diagnostic and Statistical Manual of Mental Disorders 5th Edition (DSM-5) criteria or structured interviews (30). Treatment response is monitored with interviews and rating scales of symptom severity. Clinical interviews and rating scales have inherent reporting biases. Therefore, the development of non-invasive, quantitative diagnostic, and prognostic biomarkers is essential. Previous studies suggest that LICI might have utility as a diagnostic and prognostic biomarker in neuropsychiatric disorders. There are no prior comprehensive systematic reviews examining studies of LICI. This systematic review sought to summarize the literature examining LICI alterations in brain based disorders. A second goal was to synthesize the existing evidence focused on LICI as a diagnostic and prognostic biomarker for neuropsychiatric disorders. Finally, we review recent work with LICI paradigms related to cognitive neuroscience literature and methodological challenges.

## Materials and Methods

### Search Strategy

This systematic review was conducted according to the guidelines of Preferred Reporting Items for Systematic Reviews and Meta-Analyses (PRISMA) (31, 32). The literature search was performed using the internet databases Embase, EMB Reviews, Medline, APA PsychINFO, Scopus, and Web of Science up to April 8th, 2021. The search strategy was designed in consultation with an experienced medical reference librarian. The full search strategy and search terms used for the literature search are described in the Supplementary Material.

### Study Selection

Studies were included if the following criteria were met: (a) articles in English; (b) original articles including participants with major neurologic and psychiatric disorders; (c) cortical inhibition was measured with a TMS LICI paradigm. Studies were excluded according to the following criteria: (a) animal studies; (b) review articles, letters to the editor, short communication papers, correspondence articles; (c) published conference abstracts, lectures, and presentations; and (d) study contained only healthy participants. Two authors reviewed articles for inclusion (PF and MK) The articles that met inclusion criteria were included in the review, and the articles that met exclusion criteria were excluded from the review. The senior authors (FF and PC) were consulted for any discrepancies or questions regarding inclusion of articles. The references of the included articles were reviewed, and additional papers fulfilling the eligibility criteria were included in the review.

### Data Extraction

The full texts of the eligible articles were reviewed in depth by PF and MK. Data were extracted by PF and the extracted data was verified by MK and PC, JV, and FF. Extracted data included authors, publication year, study design, number of patients and healthy controls, age and sex of control and patient group, stimulation area of cortex, muscle measured [first dorsal interosseous (FDI) and abductor pollicis brevis (APB)], diagnostic assessment instruments (EMG, EEG), stimulation parameters (stimulus intensities, interstimulus interval), medications, interventions, outcomes, and outcome measures.

### Outcomes

The primary outcome of this review focused on alterations in LICI in neuropsychiatric disorders in comparison to healthy controls. As mentioned in the introduction, LICI measurements consist of delivering two consecutive TMS pulses that are 50–200 ms apart in which a conditioning stimulus is followed by a test stimulus. LICI is quantified as the ratio of the amplitude of the evoked potential (EP) elicited following a test stimulus to the EP elicited by the conditioning stimulus (conditioned/unconditioned EP). Therefore, an increase in the exact value of the ratio (greater conditioned EP amplitude) implies reduced inhibition or lower cortical inhibition, whereas a decrease in the exact value of the ratio (smaller conditioned EP amplitude) indicates increased inhibition or greater cortical inhibition. To maintain consistent terminology throughout this review for clarity, reduced cortical inhibition will be referred as reduced/decreased LICI or LICI deficit; and increased cortical inhibition will be referred as enhanced/increased LICI. Given that both reduced and enhanced LICI have been reported in the studies included in this review, “LICI impairment” will refer to any significant difference between clinical populations and healthy controls. Additional outcomes examined correlational analyses of LICI and suicidal remission, suicidal severity, symptom severity, functional connectivity, social cognition, cortical silent period (CSP), synaptic plasticity, visuomotor reaction time, motor dexterity, GABA levels measured with proton magnetic resonance spectroscopy (^1^H-MRS), functional decline, fatigue, and cognitive functioning. The cortical areas of interest were the motor cortex and the dorsolateral pre-frontal cortex (DLPFC). The studies of interest assessed LICI with either EMG or EEG.

## Results

### Search Results

**Figure 1:**
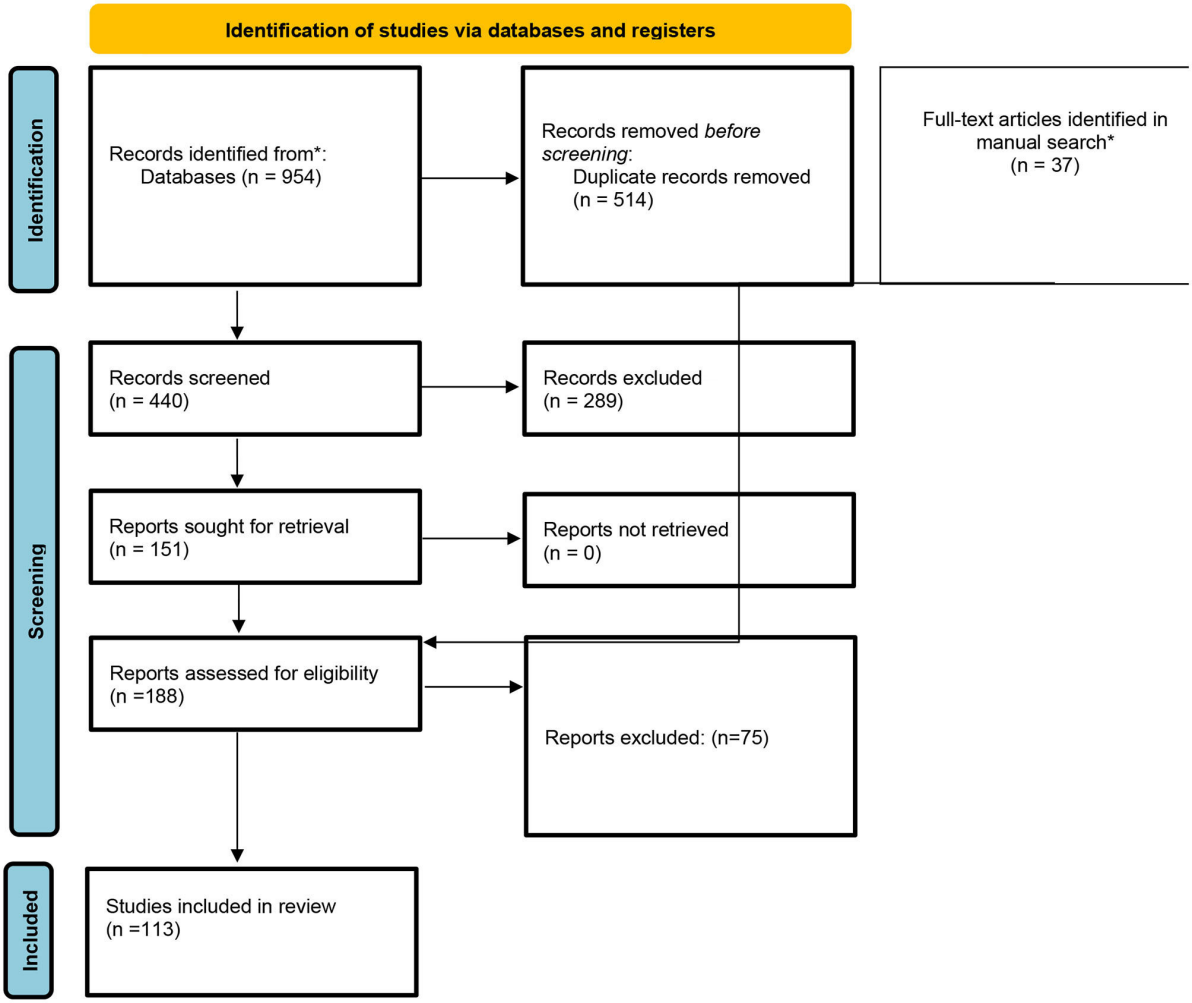
PRISMA diagram. *Records identified from the references of full-text articles.

The detailed description of the search results is shown in the PRISMA flow diagram included in Figure 1. A total of 188 articles were selected for full-text inspection after the title and abstracts of all records were screened. The reviewers identified 37 additional studies through checking the quoted references of full texts of the above-mentioned articles. Finally, 113 articles that met inclusion criteria were included and reported in the systematic review. Overall, LICI was investigated in 2 articles for ADHD, 3 articles for bipolar disorder, 9 articles for depression, 4 articles for neurodevelopmental disorders, 9 articles for schizophrenia, 5 articles for substance use, and 3 articles for other psychiatric disorders. Among the articles investigating neurological disorders, LICI was studied in 9 articles for dementia, 20 for epilepsy patients, 21 for movement disorders, 3 for multiple sclerosis, 6 for stroke patients, 12 traumatic brain injury patients and 7 for other neurological disorders. The main findings and full data extraction are summarized in the tables provided in the Supplementary Material.

### LICI in Psychiatric Disorders

#### LICI in Patients With ADHD

The LICI paradigm in ADHD patients was investigated with TMS-EMG motor cortical measures in two prior studies. One study enrolled adult subjects and the other enrolled pediatric subjects (Table 1). Buchmann et al. examined alterations in LICI among 18 children diagnosed with ADHD compared to 18 healthy control (HC) children. The study evaluated the influence of methylphenidate (MPH) treatment on LICI among children with ADHD. It was shown that baseline LICI was reduced in drug naïve ADHD subjects at an interstimulus interval (ISI) of 100 ms (*p* = 0.001). Treatment with MPH potentiated LICI yielding values similar to healthy controls. For ADHD subjects a reduction in symptoms correlated with LICI improvements following MPH administration (*p* < 0.05) (33). Hoeppner et al. evaluated 21 adult ADHD patients in a cross-sectional study and demonstrated that there was no significant LICI deficit in the patient population compared to age and gender-matched healthy controls (34). These studies indicated significant LICI deficit in children with ADHD, whereas there was no significant LICI difference between adult ADHD patients and healthy controls (33, 34). Therefore, cortical maturation with aging might explain the loss of LICI deficit in the adult ADHD population (34).

**Table 1:** LICI in patients with ADHD.

**References**	**Subjects**	**Method**	**ISI**	**LICI**
Buchmann et al. (33)	18 ADHD, 18 HC	TMS-EMG	100, 200, 300 ms	↓
Hoeppner et al. (34)	21 ADHD, 21 HC	TMS-EMG	100, 200, 300 ms	↔

*ISI, Interstimulus interval; ADHD, Attention deficit hyperactivity disorder; LICI, Long-interval intracortical inhibition; HC, Healthy controls*.

#### LICI in Patients With Bipolar Disorder

Prior studies measuring LICI in patients with bipolar disorder (BD) used TMS-EMG to investigate motor cortex activity (Table 2). Ruiz-Veguilla et al. studied trait and state-dependent LICI deficits in 19 adult patients with BD in the depressed phase vs. 28 healthy controls. In the BD sample, 15 patients who were receiving a combination of lithium/valproate plus antipsychotic treatment were assessed 3 months later to evaluate the influence of symptom remission on LICI. Results demonstrated that there was no significant LICI deficit in patients with BD vs. healthy controls at baseline, and LICI did not significantly change with symptom remission at follow-up (35). A cross-sectional study by Basavaraju et al. investigated LICI in 39 medication naïve patients with BD in a manic episode, 28 remitted first-episode mania patients treated with antipsychotic medications or an antipsychotic plus mood stabilizer, and 45 HC. The LICI measures were significantly enhanced in medication naïve patients who were in a manic episode compared to HC (*p* = 0.021). There was no significant difference in LICI between the patients who were in a manic episode and those who remitted, and there was no significant difference in LICI between the remitted patients and HC. Correlation of symptom severity, measured with Young's Mania Rating Scale (YMRS), and LICI was non-significant (36). Another cross-sectional study by Basavaraju et al. evaluated Mirror Neuron Activity (MNA) using LICI, and its correlation with symptom severity in the same group of 39 medication naïve BD patients in a manic episode and 45 HC. MNA was measured while subjects were observing a goal-directed activity. Enhanced MNA assessed with LICI was detected in the medication naïve symptomatic BD patients (*p* = 0.033). LICI mediated putative MNA was significantly correlated with symptom severity (*p* = 0.038) (37).

**Table 2:** LICI in patients with bipolar disorder.

**References**	**Subjects**	**Method**	**ISI**	**LICI**
Ruiz-Veguilla et al. (35)	19 BD, 28 HC	TMS-EMG	100, 150, 250 ms	↔
Basavaraju et al. (36)	67 BD, 45 HC	TMS-EMG	100 ms	↑
Basavaraju et al. (37)	39 BD, 45 HC	TMS-EMG	100 ms	↑

*ISI, Interstimulus interval; BD, Bipolar disorder; LICI, Long-interval intracortical inhibition; HC, Healthy controls. ↑: increased, ↔: no significant difference*.

The studies examining BD patients had varied results demonstrating both normal LICI and enhanced LICI relative to HC. These results are interesting as studies with other neurophysiological paradigms have shown inhibitory deficits in BD (36). Possible explanations and supporting evidence of enhanced LICI in bipolar disorder comes from the studies showing manic symptoms triggered with baclofen and elevated GABA/Creatine ratio in anterior cingulate cortex of patients with BD (38). These results might be affected by study flaws such as larger manic sample and medication naïve condition (36) or small sample size (35).

#### LICI in Patients With Depression

Seven prior studies in depressed patients examined cortical inhibition measured with TMS-EMG. Two other studies investigated both DLPFC and motor cortex LICI paradigms using EEG (Table 3).

**Table 3:** LICI in patients with depression.

**References**	**Subjects**	**Method**	**ISI**	**LICI**
Croarkin et al. (39)	8 treatment responder MDD, 8 treatment resistant MDD	TMS-EMG	100, 150, 200 ms	↓ in treatment resistant subjects
Croarkin et al. (40)	14 MDD 19 HC	TMS-EMG	100, 150, 200 ms	↑ with age in adolescents
Sun et al. (41)	33 TRD	TMS-EEG	100 ms	↑ at baseline correlated with decreased suicidality following MST
Sun et al. (42)	23 TRD	TMS-EEG	100 ms	↓ following MST in patient with resolved SI
Lewis et al. (43)	37depressed, 17 depressed + SB, 20 HC	TMS-EMG	100, 150, 200 ms	↓ in depressed subjects with SB
Jeng et al. (44)	20 TRD, 16 non-TRD, 36 HC	TMS-EMG	100, 200 ms	↓ in TRD subjects
Lewis et al. (45)	10 Depressed	TMS-EMG	100, 150 ms	↑ associated with decrease in SI following antidepressant treatment
Balzekas et al. (46)	5 Depressed	TMS-EMG	100, 150, 200 ms	Comparison not measured
Doruk Camsari et al. (47)	15 MDD, 22 HC	TMS-EMG	100, 150, 200 ms	↔

*ISI, Interstimulus interval; TRD, Treatment resistant depression; LICI, Long-interval intracortical inhibition; MST, Magnetic seizure therapy; MDD, Major depressive disorder; SI, Suicidal ideation; HC, Healthy controls; SB, Suicidal behavior. ↓: decreased, ↔: no significant difference*.

Croarkin et al. investigated the association of pre-treatment LICI levels with treatment response in 16 children and adolescents with major depressive disorder (MDD) who were initiated on treatment with fluoxetine. Treatment-resistant subjects had greater LICI deficits at baseline relative to treatment responders at each ISIs of 100, 150, 200 ms (*p* = 0.01, 0.03, 0.01) (39). A cross-sectional study examining the relationship between cortical inhibition and age evaluated LICI in 14 youth with MDD and 19 age-matched HC. Results suggested that increased LICI at 200 ms was associated with older age in depressed youth in both right and left hemispheres (*p* = 0.034, 0.002), whereas there was no significant association with age in the control group (40). LICI was assessed in depressed adolescents with and without a history of suicidal behavior and compared to HC in a study by Lewis et al. Depressed adolescents with a history of suicidal behavior exhibited decreased LICI at interstimulus intervals of 100 ms and 150 ms relative to HC (*p* = 0.0002, 0.0009) and depressed adolescents without suicidal behavior (*p* = 0.0049, 0.0418). Moreover, increased suicidal severity correlated with the reduction in LICI at ISI of 100 and 150 ms (43). Jeng et al. investigated LICI in 20 adult patients with treatment-resistant depression, 16 adult patients who responded to treatment for MDD, and HC to evaluate the utility of LICI as a biomarker in distinguishing treatment response. LICI was significantly reduced in treatment-resistant subjects relative to treatment responders or HC, and decreased LICI was correlated with higher symptom severity (*p* < 0.001). This study also demonstrated a reduction in LICI in treatment after 3 months of SSRI treatment (*p* = 0.002) (44). Lewis et al. examined LICI change and its association with change in suicidal ideation (SI) in depressed adolescents treated with antidepressants and found that decreased SI was associated with enhanced LICI measured at 100 ms when controlling for depression severity (*p* = 0.021). Adolescents with prior suicidal behavior had a greater reduction in follow up LICI relative to those without a previous history of suicidal behavior (*p* = 0.038) (45). In a cross-sectional study by Balzekas et al., the association between LICI and cortical connectivity, measured with resting-state functional magnetic resonance imaging, was found to be non-significant (46). Doruk Camsari et al. investigated the change of LICI with antidepressant treatment and found no significant post-treatment alteration in LICI (47).

Sun et al. investigated pre-treatment LICI in DLPFC and motor cortex, as a biomarker for suicidal remission after Magnetic Seizure Therapy (MST). This study examined 33 treatment-resistant adult depression patients and revealed that remission of suicidal ideation, measured with the Scale for Suicide Ideation (SSI), was correlated with greater LICI in DLPFC at baseline (*p* = 0.02) although pre-treatment LICI in the motor cortex was not significantly correlated with treatment outcome (41). Another study by Sun et al. examined changes in neuroplasticity, using LICI, and suicidal ideation following MST treatment in adults with treatment-resistant depression. Results showed that LICI in DLPFC was reduced following MST treatment in patients with resolved suicidal ideation and that the decrease in LICI was correlated with SSI score reduction (*p* = 0.048, 0.044). There was no significant finding for LICI in the motor cortex (42).

Two studies showed significantly more impaired LICI in treatment-resistant subjects compared to treatment responders. Furthermore, greater LICI deficits in depressed patients with suicidal ideation compared to non-suicidal depressed patients were indicated. Supporting this, enhanced LICI following MST treatment was shown to predict the reduction in suicidal ideation while another study showed the correlation of alteration in LICI and the improvement in suicidal ideation. These results are consistent with the findings showing both GABA_A_ and GABA_B_ mediated cortical inhibitory deficits in the pathophysiology of depression (14) in different age groups (48). Moreover, these findings suggest that LICI might be a used as a prognostic biomarker evaluating depressed patients. Regarding suicidal ideation and suicidal behavior, literature was limited to the papers in our review. It is known that impaired behavioral inhibition (i.e., impulsivity) is central to the pathophysiology of suicide. It might be postulated that cognitive and behavioral inhibitory deficits result from cortical inhibition alterations in brain regions vital for inhibitory control, such as the anterior cingulate gyrus (49, 50).

#### LICI in Patients With Neurodevelopmental Disorders

LICI in neurodevelopmental disorders was investigated in Fragile X and patients with autism spectrum disorder (ASD) using TMS-EMG (Table 4). Oberman et al. evaluated LICI in 2 subjects with Fragile X, 2 with ASD, and 5 HC and found no significant LICI difference across these three groups (51). Another study examining LICI alteration in ASD with a larger sample size of 36 patients with ASD and 34 HC had similar results showing no significant LICI deficit in individuals with ASD (52). Morin-Parent et al. studied LICI in 18 individuals with molecular Fragile X diagnosis (7 of them receiving psychotropic medication) and compared these patients to 18 age and gender-matched HC, and results demonstrated that LICI was enhanced in Fragile X subjects relative to HC (*p* = 0.011). When the analysis was limited to only non-medicated Fragile X individuals, results demonstrated a similar trend of enhanced LICI although, the significance was lost (*p* = 0.060) (53). Bernardo et al. investigated LICI in Rett syndrome patients comparing them to non-Rett syndrome epilepsy patients and health controls. LICI was reduced in the Rett syndrome patients relative to epilepsy and healthy controls (*p* = 0.002). Furthermore, impaired motor performance was significantly associated with decreased LICI (*p* = 0.003) (54).

**Table 4:** LICI in patients with neurodevelopmental disorders.

**References**	**Subjects**	**Method**	**ISI**	**LICI**
Oberman et al. (51)	2 Fragile X, 2 ASD, 5 HC	TMS-EMG	100 ms	↔
Enticott et al. (52)	36 ASD, 34 HC	TMS-EMG	100 ms	↔
Morin-Parent et al. (53)	18 Fragile X, 18 HC	TMS-EMG	100 ms	↑
Bernardo et al. (54)	14 Rett syndrome, 9 epilepsy control, 11 HC	TMS-EMG	100, 150 ms	↓ in Rett syndrome patients

*ISI, Interstimulus interval; ASD, Autism spectrum disorder; LICI, Long-interval intracortical inhibition; HC, Healthy controls. ↑: increased, ↔: no significant difference*.

#### LICI in Patients With Schizophrenia Spectrum Disorders

Five prior studies examined the motor cortex, TMS-EMG LICI in patients with schizophrenia (SCZ). Three studies focused on both DLPFC and motor cortex, and 1 study focused on only DLPFC using EEG (Table 5).

**Table 5:** LICI in patients with schizophrenia.

**References**	**Subjects**	**Method**	**ISI**	**LICI**
Fitzgerald et al. (55)	18 SCZ, 8 HC	TMS-EMG	100 ms	↔
Farzan et al. (56)	14 SCZ, 14 BD, 14 HC	TMS-EEG	100 ms	↓ in DLPFC of SCZ, ↔ in BD
Mehta et al. (57)	54 SCZ, 45 HC	TMS-EMG	100 ms	↔
Mehta et al. (58)	54 SCZ, 45 HC	TMS-EMG	100 ms	↔ MNA
Radhu et al. (59)	38 SCZ, 27 OCD, 46 HC	TMS-EEG	100 ms	↓ in DLPFC of SCZ, ↔ in OCD
Basavaraju et al. (60)	18 SCZ with EBD, 32 SCZ w/o EBD	TMS-EMG	100 ms	↔
Lett et al. (61)	80 SCZ, 115 HC	TMS-EEG	100 ms	↔
Radhu et al. (62)	19 SCZ, 30 FDR of SCZ, 13 OCD, 18 FDR of OCD, 49 HC	TMS-EEG	100 ms	↓ in DLPFC of SCZ, ↔ in OCD
Goodman et al. (63)	12 SCZ with cannabis use, 11 cannabis free SCZ, 10 controls with cannabis use, 13 cannabis free controls	TMS-EMG	100, 150, 200 ms	↔

*ISI, Interstimulus interval; HC, Healthy controls; LICI, Long-interval intracortical inhibition; MNA, Mirror neuron activity; DLPFC, Dorsolateral pre-frontal cortex; OCD, Obsessive-compulsive disorder; SCZ, Schizophrenia; EBD, Ego boundary disturbance; BD, Bipolar disorder; FDR, First degree relative. ↓: decreased, ↔: no significant difference*.

In a study including 18 SCZ patients (9 were medicated with antipsychotics) and 18 HC, Fitzgerald et al. demonstrated that there was no significant difference across groups regarding LICI (55). A cross-sectional study by Mehta et al. examined a larger sample size of 54 SCZ patients and 45 HC, and they also found no significant LICI difference in SCZ patients relative to HC. There was also no significant correlation between social cognition measures and LICI in both groups (57). Another study by Mehta et al. evaluated MNA using LICI in the same group of above-mentioned subjects and showed that action observation did not have a significant effect on LICI. Further, there was no significant correlation between MNA and social cognition in both SCZ patients and HC (58). Basavaraju et al. examined LICI to investigate MNA with a sample of 50 SCZ patients, 18 with ego boundary disturbances (EBD) and 32 without EBD, and demonstrated no significant MNA difference between groups (60). Goodman et al. examined the effect of cannabis use on LICI in SCZ patients in a study involving 4 groups of subjects: 12 cannabis dependent SCZ patients, 11 cannabis free SCZ patients, 10 cannabis dependent controls, and 13 cannabis free controls. The findings demonstrated that cannabis use did not significantly alter LICI in either the SCZ group or control group; likewise, LICI was not significantly different in SCZ subjects and controls (63).

Farzan et al. investigated alterations in LICI assessed with gamma oscillations in DLPFC and motor cortex of 14 patients with SCZ, 14 patients with BD, and 14 HC. The study revealed that SCZ patients had significantly impaired cortical inhibition of gamma oscillations in DLPFC relative to BD patients and HC (*p* < 0.01, *p* < 0.01), whereas there was no significant difference between BD subjects and HC. Notably, in this study, LICI measured from the motor cortex did not differ significantly across SCZ patients, BD patients, and HC (56). Another study targeting DLPFC in SCZ patients as compared to OCD patients and HC demonstrated that SCZ patients had significantly greater LICI reduction relative to OCD group and HC (*p* = 0.0465, 0.004, respectively). There were no significant differences between the OCD and HC samples. Once again there were no significant differences in LICI measures from the motor cortex among the three groups. However, the study revealed that SCZ symptom severity, measured with the Brief Psychiatric Rating Scale (BPRS), was correlated with a deficit in LICI (*p* = 0.0457) (59). Radhu et al. examined LICI in SCZ and OCD patients and their unaffected first-degree relatives compared to HC. Results exhibited that SCZ patients had greater LICI deficit in the DLPFC relative to first-degree relatives (*p* = 0.03) and HC (*p* = 0.032). First degree relatives had impaired LICI compared to HC patients, but this was not statistically significant. LICI measures in the motor cortex did not differ across SCZ patients, first-degree relatives, and HC. Likewise, there was no significant difference across OCD patients, first-degree relatives, and HC in either the motor cortex or DLPFC (62).

Lett et al. investigated LICI in the DLPFC using TMS-EEG and examined the correlation between GAD glutamic acid decarboxylase 1 (GAD1) variant and LICI in SCZ patients and HC. It was shown that GAD1 T allele carrier healthy controls had greater LICI cluster size (*p* = 0.003), whereas patients with SCZ who were allele carriers had a lower cluster size (0.04) (61).

The studies examining LICI in motor cortex of SCZ patients failed to show any significant correlation between LICI alterations and neural correlates of SCZ symptoms (i.e., MNA for ego boundary disturbances). Whereas, studies investigating LICI in DLFPC of SCZ demonstrated significant findings. The dysfunctional frontal inhibitory neurotransmission might be underlying the cognitive function deficits present in SCZ principally the working memory performance (59, 64, 65).

#### LICI in Patients With Substance Use Disorder

Prior studies examined LICI in nicotine, cocaine, alcohol, and cannabis users (Table 6). Four of those studies examined the motor cortex using TMS-EMG, whereas one study investigated the DLPFC using TMS-EEG. Another study investigating the effects of cannabis use on LICI in SCZ patients is mentioned above in the “LICI in patients with Schizophrenia Spectrum Disorders” section.

**Table 6:** LICI in patients with substance use.

**References**	**Subjects**	**Method**	**ISI**	**LICI**
Sundaresan et al. (66)	10 cocaine users, 10 HC	TMS-EMG	50, 100 ms	↔
Lang et al. (67)	19 nicotine users, 19 HC	TMS-EMG	50, 100, 150 ms	↔
Fitzgerald et al. (68)	42 cannabis user, 19 HC	TMS-EMG	100 ms	↔
Gjini et al. (69)	52 cocaine users, 42 HC	TMS-EMG	50, 100 ms	↔
Naim-Feil et al. (70)	12 alcohol dependent, 14 HC	TMS-EEG	100 ms	↓ in DLPFC

*ISI, Interstimulus interval; HC, Healthy controls; LICI, Long-interval intracortical inhibition; DLPFC, Dorsolateral pre-frontal cortex. ↓: decreased, ↔: no significant difference*.

In a cross-sectional study involving 10 abstinent cocaine-dependent subjects and 10 HC, results demonstrated no significant LICI deficit in cocaine users relative to the control group (66). Gjini et al. confirmed the same finding, showing no significant difference of LICI, in their study evaluating 52 abstinent cocaine-dependent subjects and 42 HC (69). Lang et al. demonstrated that there were no differences in LICI among subjects using nicotine and subjects who were not using nicotine (67). Fitzgerald et al. investigated LICI alterations in 42 chronic cannabis users and showed that there was no significant difference in LICI relative to the non-user control group (68).

Naim-Feil et al. investigated the alteration of LICI in DLPFC of 12 alcohol-dependent subjects post-detoxification and 14 HC. Results revealed that the alcohol-dependent group had greater reduction in LICI in both left and right DLPFC relative to the control group (*p* = 0.003, *p* = 0.006) (70).

The studies regarding substance use disorders suggested that LICI measurements from DLPFC tend to reveal more significant differences than LICI measurements from motor cortex. The prior negative findings with LICI paradigms in substance use disorders are important to ponder and reconcile with pre-clinical studies showing the alterations in the GABAergic changes of brain after chronic cocaine administration (71, 72) as well as modulatory effects of GABAergic agents in cocaine addiction treatment (73, 74). As mentioned in the above studies alterations in GABAergic circuits were in the relative brain regions for addiction, such as the DLPFC [59]. Collectively, these findings suggest that patients with substance use disorders may have underlying GABAergic deficits in pre-frontal neurocircuitry. Further studies utilizing TMS-EEG to investigate the cortical inhibition in potentially affected brain regions are essential to explicate the role of LICI in substance use disorders.

#### LICI in Patients With Other Psychiatric Disorders

A study investigating LICI over DLPFC and motor cortex in psychopathic offenders through TMS-EEG indicated that LICI was impaired in DLPFC (*p* = 0.005) but not in the motor cortex relative to healthy subjects (Table 7). Moreover, psychopathic offenders displayed worse working memory performance (*p* = 0.005), measured with the letter-number sequencing test, which demonstrated a non-significant but trending correlation with LICI in DLPFC (*p* = 0.069). Healthy subjects showed better working memory performance associated with greater LICI in DLPFC (*p* = 0.005) (75). Salas et al. showed that there were no abnormalities of motor cortex LICI in individuals with chronic insomnia relative to good sleepers (76). LICI alteration was investigated by Li et al. evaluating 26 patients with generalized anxiety disorder who were medication naive and 35 age and sex-matched controls, and it was demonstrated that LICI did not differ significantly in patients with anxiety relative to HC. Nevertheless, decrements in LICI correlated with higher symptom scores measured with the Hamilton Anxiety Rating Scale (*p* = 0.020) (77).

**Table 7:** LICI in patients with other psychiatric disorders.

**References**	**Subjects**	**Method**	**ISI**	**LICI**
Hoppenbrouwers et al. (75)	13 psychopathic offenders, 15 HC	TMS-EEG	100, 150, 250 ms	↓ in DLPFC
Salas et al. (76)	18 chronic insomnia, 10 HC	TMS-EMG	100 ms	↔
Li et al. (77)	26 GAD, 35 HC	TMS-EMG	100 ms	↔

*ISI, Interstimulus interval; HC, Healthy controls; LICI, Long-interval intracortical inhibition; DLPFC, Dorsolateral pre-frontal cortex; GAD, Generalized anxiety disorder. ↓: decreased, ↔: no significant difference*.

### LICI in Neurologic Disorders

#### LICI in Patients With Dementia

Among 9 studies investigating the role of LICI in dementia, 6 studied subjects with frontotemporal dementia (FTD), 2 with Alzheimer's disease (AD), and 1 investigated both disorders (Table 8). The motor cortex was the area of interest for all the studies. Brem et al. studied LICI alterations in 7 AD patients taking acetylcholinesterase inhibitor (AChEI) medication, 9 AD patients taking AChEI medication with memantine, and 13 HC. The study demonstrated reductions in LICI in the group receiving AChEI medications and those receiving AChEI medication+memantine relative to HC subjects (*p* = 0.025, 0.015). Impairments in cognitive functioning, measured with Alzheimer's Disease Assessment Scale-Cognitive Subscale (ADAS-Cog), were significantly associated with LICI deficit (*p* = 0.010) (78). Benussi et al. examined the LICI alterations in FTD mutation carriers, 13 pre-symptomatic and 14 symptomatic, relative to HC. Although there was no significant LICI deficit in FTD mutation carriers, symptomatic carriers had greater LICI deficit compared to the other groups (79). In a study investigating the role of LICI as a biomarker distinguishing AD from FTD (*n* = 79 AD, *n* = 61 FTD, *n* = 32 HC) Benussi et al. did not detect any significant difference of LICI between AD and FTD patients or AD patients and HC. The LICI impairment in the FTD group was significantly greater relative to controls at ISI of 150 ms (80). Fried et al. demonstrated LICI's high reproducibility in AD patients and HC with a prospective cohort study (α = 0.88, 0.98) (81). Another study by Benussi et al. examined LICI as a disease progression biomarker in FTD mutation carriers and non-carrier at-risk individuals. 113 subjects carrying monogenic FTD mutation and 75 non-carriers with affected first-degree relatives were evaluated, and years from symptom onset were determined by subtracting the age of the participant from mean familial age at symptom onset. Reduced LICI was detected in mutation carriers relative to non-carriers at 20 years before expected symptom onset (*p* < 0.001) (82). Assogna et al. examined change in LICI following palmitoylethanolamide luteoline (PEA-LUT) administration and found that LICI was increased after PEA-LUT (83). In another cross-sectional study the same group demonstrated that LICI was more reduced in GRN mutation carriers relative to non-carrier first-degree relatives and there was no significant association of LICI with behavioral symptoms (84). LICI alteration in different phenotypes of FTD and its correlation with functional decline and symptom severity were studied in another study of Benussi et al. LICI was demonstrated to be impaired in all phenotypes (behavioral variant of FTD, agrammatic variant of Primary Progressive Aphasia, semantic variant of PPA) of FTD relative to HC (*p* < 0.05). Disease duration, functional decline, and increased symptom severity were significantly associated with LICI deficit in FTD patients (*p* < 0.001) (85). LICI's association with brain network connectivity and fluidity was examined by Benussi et al. and they indicated that there was no significant relation (86).

**Table 8:** LICI in patients with dementia.

**References**	**Subjects**	**Method**	**ISI**	**LICI**
Brem et al. (78)	16 AD, 13 HC	TMS-EMG	100 ms	↓
Benussi et al. (79)	27 FTD, 24 HC	TMS-EMG	50, 100, 150 ms	↔
Benussi et al. (80)	79 AD, 61 FTD, 32 HC	TMS-EMG	50, 100, 150 ms	↓ in FTD, ↔ in AD
Fried et al. (81)	9 AD, 15 DM, 12 HC	TMS-EMG	100 ms	High reproducibility of LICI indicated
Benussi et al. (82)	113 FTD mutation carrier FDR, 75 mutation non-carrier FDR	TMS-EMG	50, 100, 150 ms	↓ in FTD mutation carriers
Assogna et al. (83)	17 probable FTD	TMS-EMG	50, 100, 150 ms	↑ following palmitoylethanolamide luteoline administration
Benussi et al. (84)	186 FTD	TMS-EMG	50, 100, 150 ms	↓ in GRN mutation carriers
Benussi et al. (85)	171 FTD, 74 HC	TMS-EMG	50, 100, 150 ms	↓
Benussi et al. (86)	66 FTD	TMS-EMG	50, 100, 150 ms	↔ no association with whole brain fluidity

*ISI, Interstimulus interval; HC, Healthy controls; LICI, Long-interval intracortical inhibition; FTD, Frontotemporal dementia; AD, Alzheimer's disease; GRN, Granulin; FDR, First degree relative*.

These studies of LICI have examined Alzheimer's disease and Frontotemporal Dementia. Alzheimer studies have presented varied results. Several prior studies have shown the modulatory effect of GABA_B_ stimulation in Alzheimer's disease (87). Interaction between the cholinergic and GABAergic systems as well as inhibitory interactions between SAI and LICI might explain the difference between AD group and HC. However, the findings in FTD patients were quite consistent, showing LICI deficits in the patient population. The results showing LICI deficit detected before symptom onset in FTD mutations carriers and LICI's correlation with functional decline underlie the potential utility of LICI as a biomarker estimating the disease progression.

#### LICI in Patients With Epilepsy

LICI in epilepsy was studied in 20 studies (Table 9). Brodtmann et al. demonstrated reduced LICI at ISI of 200–300 ms in idiopathic generalized epilepsy (IGE) patients, and significant facilitation instead of inhibition was observed at the same intervals in the IGE group (*p* < 0.05) (88). Valzania et al. demonstrated impaired LICI in progressive myoclonic epilepsy patients relative to HC at the ISI of 100–150 ms and facilitation of motor evoked potential at 50 ms ISI (*p* < 0.001) (89). Manganotti et al. studied juvenile myoclonic epilepsy patients and found no significant LICI difference relative to HC (90). Molnar et al. did not find any significant effect of bilateral anterior thalamus deep brain stimulation (DBS) on LICI in epilepsy patients, and LICI was impaired in all DBS stimulus conditions (off, cycling, and continuous) at ISI of 50 ms (*p* = 0.0003, 0.0015, 0.0001) (91). Badawy et al. demonstrated facilitation instead of inhibition in both hemispheres of IGE patients using the LICI paradigm; thus, LICI was reduced relative to HC (*p* < 0.01 at ISI of 250 ms). Similar findings were also demonstrated with ipsilateral LICI measures of focal epilepsy patients (*p* < 0.01 at ISI of 250 ms) but there was no significant impairment in the contralateral hemisphere relative to HC (92). Additionally, Badawy et al. examined the effect of the antiepileptic drug treatment in epilepsy patients and showed that in seizure-free IGE patients, LICI was restored post-treatment in the dominant hemisphere at interstimulus intervals of 50, 150, 250, and 300 ms (p < 0.01). Focal epilepsy patients who were seizure-free post-treatment also had restored LICI in the ipsilateral hemisphere at interstimulus intervals of 250–300 ms (*p* < 0.05). IGE and focal epilepsy patients with ongoing seizures did not have any significant change in LICI post-treatment (93). Badawy and Jackson examined LICI in migraine and epilepsy patients and demonstrated LICI impairment in migraine, IGE, and focal epilepsy patients relative to HC at the interstimulus interval of 250 ms (*p* < 0.05). Results showed that LICI had greater reduction in focal epilepsy (*p* < 0.05) and IGE patients (*p* < 0.01) relative to migraine patients (94). In a prospective cohort study, the reproducibility of LICI in drug naïve epilepsy patients was investigated by measuring LICI in two separate sessions 4–20 weeks apart. Results suggested that LICI was reduced in epilepsy patients relative to HC at baseline, and there was no significant intersession variability in both patient and control groups (95). In another longitudinal study, Badawy et al. investigated the effect of the antiepileptic drug on LICI over time by evaluating epilepsy patients in 4 phases 1–2 weeks, 2–6, 12–18, and 30–36 months apart. Results showed that refractory IGE and focal epilepsy patients demonstrated worsening LICI over time whereas in seizure-free IGE and focal epilepsy patients, significant improvement of LICI and decreased cortical excitability was observed with antiepileptic medications (96). In a cross-sectional study, Badawy et al. compared patients with juvenile myoclonic epilepsy (JME), juvenile absence epilepsy (JAE), and generalized epilepsy with tonic-clonic seizures (GE-TCS). All drug naïve patients (JME, JAE, and GE-TCS) groups demonstrated lower LICI relative to HC (*p* < 0.01). JME patients had greater impairment in LICI compared to JAE and GE-TCS subjects (*p* < 0.05) (97). Temporal lobe epilepsy (TLE) patients were examined for LICI in a cross-sectional study by Badawy et al. and results revealed that LICI in the ipsilateral hemisphere of drug naïve TLE patients was reduced relative to HC (*p* < 0.01). Refractory TLE subjects demonstrated the same findings but LICI impairment also applied for the contralateral hemisphere, and the refractory group demonstrated more reduced LICI compared to seizure-free and drug naïve TLE patients (98). Another cross-sectional study evaluated the role of glucose levels in LICI alteration and found that in both healthy controls and epilepsy patients (IGE and focal epilepsy) LICI in both hemispheres was reduced in the fasting state relative to the postprandial state (*p* < 0.05) (99). In another study, Badawy et al. included patients having isolated unprovoked seizures and revealed this sample of patients had reduced LICI relative to HC (*p* < 0.01), though impairment in LICI was lower compared to IGE patients (*p* < 0.01) and focal epilepsy patients (*p* < 0.05) (100). Focal epilepsy patients with different epileptogenic regions were examined in a cross-sectional study, and it was demonstrated that LICI was reduced in drug naïve, refractory, and seizure-free TLE and extra-TLE patients relative HC (*p* < 0.05). Notably, in drug naïve and seizure-free patients significant LICI impairment was only seen in the ipsilateral hemisphere. Refractory groups demonstrated lower LICI relative to seizure-free and drug naïve TLE and extra-TLE patients (*p* < 0.05) (101). Silbert et al. studied IGE patients and found that LICI was not significantly different between unmedicated IGE patients and HC, whereas IGE patients on antiepileptic drug treatment had enhanced LICI relative to unmedicated IGE patients (*p* <0.001) and HC (*p* = 0.003) (102). Pawley et al. investigated LICI in longstanding uncontrolled epilepsy patients and revealed that LICI in poorly controlled or moderately controlled generalized epilepsy patients did not differ significantly from HC. In focal epilepsy patients, poorly controlled and moderately controlled epilepsy was associated with enhanced LICI compared to HC (*p* = 0.040) (103). Bauer et al. demonstrated similar findings showing no significant LICI differences across HC, IGE, and focal epilepsy patients (104). Bolden et al. also found that controlled IGE patients, treatment resistant IGE patients, and HC did not have a significantly different LICI. Nevertheless, results demonstrated that patients with lower LICI performed worse in attention tasks (105). A companion study by the same research group found that participants with the excitatory response on LICI demonstrated greater mood disturbance relative to participants with the inhibitory response (106). In a cross-sectional study, Huang et al. studied TLE patients and demonstrated that LICI was stronger in poorly controlled and well-controlled TLE patients relative to HC at interstimulus intervals of 50, 100, and 200 ms (*p* = 0.026, 0.002, 0.001) (107).

**Table 9:** LICI in patients with epilepsy.

**References**	**Subjects**	**Method**	**ISI**	**LICI**
Brodtmann et al. (88)	7 IGE, 16 HC	TMS-EMG	50–400 ms	↓
Valzania et al. (89)	12 IGE, 8 HC	TMS-EMG	50, 100, 150, 250 ms	↓
Manganotti et al. (90)	15 JME, 12 HC	TMS-EMG	30–400 ms	↔
Molnar et al. (91)	5 Epilepsy, 9 HC	TMS-EMG	50–200 ms	↓
Badawy et al. (92)	35 IGE, 27 focal epilepsy, 29 HC	TMS-EMG	200–400 ms	↓ in IGE and focal epilepsy
Badawy et al. (93)	59 IGE, 47 focal epilepsy, 32 HC	TMS-EMG	50–300 ms	↓ at baseline, ↑ following AED treatment in seizure free subjects
Badawy and Jackson (94)	26 migraine, 22 focal epilepsy, 28 IGE 19 HC	TMS-EMG	50–400 ms	↓ in migraine, IGE and focal epilepsy
Badawy et al. (95)	11 focal epilepsy, 13 IGE, 17 HC	TMS-EMG	50–400 ms	↓ IGE and focal epilepsy
Badawy et al. (96)	30 refractory epilepsy, 35 seizure free on monotherapy, 12 seizure free on dual therapy	TMS-EMG	100–300 ms	↓
Badawy et al. (97)	46 JME, 41 JAE, 50 GE-TCS, 20 HC	TMS-EMG	100–300 ms	↓
Badawy et al. (98)	85 TLE, 20 HC	TMS-EMG	100–300 ms	↔
Badawy et al. (99)	11 IGE, 11 focal epilepsy, 10 HC	TMS-EMG	100–400 ms	↓
Badawy et al. (100)	21 isolated seizure, 20 IGE, 18 focal epilepsy, 20 HC	TMS-EMG	100–300 ms	↓ in IGE and focal epilepsy
Badawy et al. (101)	46 TLE, 39 Extra-TLE, 20 HC	TMS-EMG	100–300 ms	↓ at baseline, ↑ following AED treatment in seizure free subjects
Silbert et al. (102)	10 IGE, 12 HC	TMS-EMG	100–350 ms	↓ in migraine, IGE, and focal epilepsy
Pawley et al. (103)	28 moderately controlled epilepsy, 40 poorly controlled epilepsy, 28 HC	TMS-EMG	50–250 ms	↓ IGE and focal epilepsy
Bauer et al. (104)	40 IGE, 69 focal epilepsy, 95 HC	TMS-EMG	50–250 ms	↓
Bolden et al. (105)	30 IGE, 24 HC	TMS-EEG	50–200 ms	↓
Bolden et al. (106)	30 IGE, 22 HC	TMS-EEG	50–200 ms	↔
Huang et al. (107)	41 poorly controlled TLE, 71 well-controlled TLE, 44 HC	TMS-EMG	50–300 ms	↓

*ISI, Interstimulus interval; JAE, juvenile absence epilepsy; LICI, Long-interval intracortical inhibition; GE-TCS, Generalized epilepsy with tonic-clonic seizures; IGE, Idiopathic generalized epilepsy; TLE, Temporal lobe epilepsy; HC, Healthy controls; AED, Antiepileptic drug; JME, Juvenile myoclonic epilepsy; PME, Progressive myoclonic epilepsy*.

The majority of the studies demonstrated reduced LICI in patients with various seizure disorders. However, some studies demonstrated normal or increased LICI in patients with epilepsy. It would be anticipated that patients with seizure disorders have GABAergic inhibitory deficits, as this is supported by previous studies that have consistently shown the alterations in GABAergic transmission, especially in patients with absence seizures as well as in mouse models of generalized and focal seizures (108). The non-significant findings in studies of LICI with epilepsy patients might related to small intertrial intervals, the selection of the hemisphere (i.e., dominant vs. ipsilateral) examined in analyses (104) or the criteria used to classify participants as treatment-refractory (105).

#### LICI in Patients With Movement Disorders

LICI was investigated in dystonia, Huntington's disease, and Parkinson's disease patients (Table 10). Chen et al. studied 8 patients with writer's cramp and 18 HC in a cross-sectional study and showed that LICI measured from the left (symptomatic) hemisphere at 50–80 ms interstimulus interval was reduced in dystonia patients relative to HC during voluntary muscle contraction (*p* = 0.02) (111). The study did not find any significant results for LICI alteration at rest and in the right hemisphere. In another cross-sectional study, Espay et al. compared LICI in psychogenic dystonia, organic dystonia patients, and HC during rest and active muscle contraction. The results indicated that organic dystonia patients had an impaired LICI relative to healthy controls at rest (*p* = 0.009). Psychogenic dystonia patients did not differ significantly from controls at rest. However, psychogenetic dystonia patients demonstrated significantly greater LICI compared to patients with organic dystonia. Results for LICI measured during active muscle contraction were not significant (121). Meunier et al. examined the influence of paired associative stimulation and motor learning on LICI in dystonia patients. It was demonstrated that LICI decreased following learning of simple motor tasks and paired associative stimulation in HC (*p* < 0.01), but both did not have a significant effect in dystonia patients (125). In a cross-sectional study Latorre et al. examined LICI alteration in dystonic syndrome, primary writing tremor and essential tremor patients relative to HC. Baseline LICI was not significantly different across groups but paired associative plasticity induced LICI was significantly decreased in essential tremor patients and HC whereas it did not change in dystonic syndrome and primary writing tremor patients (129).

**Table 10:** LICI in patients with movement disorders.

**References**	**Subjects**	**Method**	**ISI**	**LICI**
Berardelli et al. (109)	20 PD, 11 HC	TMS-EMG	100–250 ms	↑
Tegenthoff et al. (110)	13 HD, 21 HC	TMS-EMG	1–999 ms	Prolonged in classical HD patients
Chen et al. (111)	8 Dystonia, 18 HC	TMS-EMG	20–200 ms	↓ during voluntary contraction, ↔ at rest
Valzania et al. (112)	13 PD, 12 HC	TMS-EMG	40–300 ms	↑
Romeo et al. (113)	10 ET, 8 HC	TMS-EMG	100, 150, 200 ms	↔
Rona et al. (114)	10 Dystonia, 11 HC	TMS-EMG	100–250 ms	↑
Priori et al. (115)	16 HD, 28 HC	TMS-EMG	100–250 ms	↔
Chen et al. (116)	7 HD, 7 HC	TMS-EMG	50–200 ms	↔
Pierantozzi et al. (117)	29 PD, 29 HC	TMS-EMG	20–200 ms	↓, restored following Apomorphine
Cunic et al. (118)	12 PD, 8 HC	TMS-EMG	50–200 ms	↔
Bares et al. (119)	12 PD, 10 HC	TMS-EMG	200–250 ms	↔
Sailer et al. (120)	10 PD, 10 HC	TMS-EMG	100 ms	↔
Espay et al. (121)	18 dystonia, 12 HC	TMS-EMG	50–200 ms	↓
Cantello et al. (122)	18 PD, 12 HC	TMS-EMG	50–300 ms	↑
Fierro et al. (123)	14 PD, 8 HC	TMS-EMG	80 ms	↓ in non-medicated PD patients
Chu et al. (124)	11 PD, 9 HC	TMS-EMG	100, 150 ms	↓
Meunier et al. (125)	17 dystonia, 19 HC	TMS-EMG	90 ms	↔
Barbin et al. (126)	20 PD, 10 HC	TMS-EEG	100 ms	↓ in dyskinetic PD patients
Lu et al. (127)	12 PD, 12 ET, 12 HC	TMS-EEG	100 ms	Comparison not measured
Philpott et al. (128)	28 HD, 17 HC	TMS-EMG	100 ms	↓
Latorre et al. (129)	10 DTS, 7 PWT, 10 ET, 10 HC	TMS-EEG	100 ms	↔ baseline, PAS induced LICI ↓ in ET and HC

*ISI, Interstimulus interval; ET, Essential tremor; LICI, Long-interval intracortical inhibition; PWT, Primary writing tremor; PD, Parkinson's Disease; DTS, Dystonic syndrome; HC, Healthy controls; PAS, Paired associative stimulation; HD, Huntington's disease. ↑: increased, ↓: decreased, ↔: no significant difference*.

LICI alteration in Huntington's disease (HD) was investigated by Tegenthoff et al. and the results of their study demonstrated that LICI was prolonged in classical hypotonic-hyperkinetic HD patients relative to HC. In contrast, Westphal variant HD patients had a shortened LICI relative to classical type HD patients (*p* < 0.05) (110). A cross-sectional study by Priori et al. indicated that there were no significant LICI differences across HD patients and HC (115). Philpott et al. compared asymptomatic HD patients, symptomatic HD patients, and HC and found that both patient groups had a significantly impaired LICI relative to controls (*p* = 0.02). In pre-HD patients, LICI deficit was correlated with the number of CAG repeats (*p* = 0.01), and LICI was negatively correlated with behavioral symptoms in both groups (128).

Berardelli et al. examined LICI measures in Parkinson's disease (PD) and the potential impact of L-dopa treatment on LICI. The results revealed that PD patients had an enhanced LICI relative to HC at interstimulus intervals of 150 and 200 ms (*p* < 0.05). Following L-dopa treatment, LICI values were restored approaching healthy subjects (*p* = 0.01) (109). Valzania et al. demonstrated similar findings showing enhanced LICI in PD patients relative to HC at interstimulus intervals of 40, 50, and 75 ms (*p* < 0.01) (112). Chen at al. studied 7 PD patients with Globus Pallidus internus (GPi) stimulators and did not show any significant difference in LICI between PD patients and HC. Additionally, there was no difference in LICI in patients with PD when the GPi stimulator was turned on, off, or to half the amplitude (116). Pierantozzi et al. investigated the effect of apomorphine infusion on LICI in PD patients and demonstrated that baseline LICI was reduced in PD patients relative to HC and apomorphine infusion enhanced and restored LICI (117). Cunic et al. studied PD patients with subthalamic nucleus stimulators and showed that there was no significant LICI difference between patients and HC at baseline, and stimulation conditions did not affect LICI levels in PD patients (118). In a cross-sectional study, Bares et al. did not find any significant LICI impairment in L-dopa or dopamine agonist naïve PD patients relative to HC (119). Sailer et al. presented similar findings in their study, showing no significant LICI alteration in PD patients relative to HC both in the presence or absence of dopaminergic medications (120). Cantello et al. had contradictory results demonstrating enhanced LICI in PD patients relative to HC in both affected and less affected hemispheres at only 250 ms interstimulus interval (122). Fierro et al. collected LICI measures in PD patients with and without L-dopa treatment and following rTMS. LICI was reduced in patients with PD who were not taking L-dopa relative to those who were taking L-dopa (*p* = 0.005) and HC (*p* < 0.016). LICI improved following rTMS in PD patients without L-dopa (*p* < 0.01), but there was no significant effect of rTMS in PD patients who were taking L-dopa (123). LICI reduction in PD patients both on and off medication relative to HC was demonstrated by Chu et al. at interstimulus intervals of 100–150 ms (*p* = 0.035) (124). Barbin et al. compared dyskinetic PD patients to non-dyskinetic PD patients on and off L-dopa treatment. The results revealed that among medication and unmedicated PD subjects, dyskinetic PD patients had a reduced LICI relative to HC (*p* < 0.05). Conversely, there was no significant difference between non-dyskinetic PD patients and controls. LICI was only significantly different between dyskinetic and non-dyskinetic patients when L-dopa was present (*p* < 0.05) (126). Lu et al. examined the effect of paired associative stimulation (PAS) on LICI in Essential Tremor (ET), PD patients, and HC and demonstrated that PAS induced a reduction in LICI irrespective of group (*p* < 0.01) (127). Patients with essential tremor were investigated by Remeo et al. and there was no significant difference in LICI between the patient group and HC (113).

Studies examining LICI in patients with Parkinson's disease have presented mixed results. Several studies have shown enhanced LICI in the patient population. One suggested mechanism for this difference was larger motor-evoked potentials (MEPs) of conditioning stimulus (109). Conversely, several other studies have shown impairment in LICI, which is consistent with repeated findings of a shorter silent period in patients with Parkinson's disease (16, 130). Decreased MEP facilitation of test stimulus in patients due to increased tonic activity might explain the impairments in LICI (122). Studies have varied in their methodology in terms of the Parkinson's disease treatment and TMS protocol, which might also contribute to the discrepancies in findings. Findings also varied in Huntington's disease some results indicating normal LICI and some showing reduced LICI in HD patients. Inhibition impairments in HD have been shown before and might be attributed to increased excitability and constant preparation for movement (128). Discrepancies between findings could be related to the methodologic issues that might confound the results such as coil type and active contraction vs. resting muscle. In dystonia, studies have shown LICI impairment in dystonic patients compared to healthy controls. Previous studies showing the effectiveness of GABA_B_ receptor agonist baclofen also support our findings (131, 132).

#### LICI in Patients With Multiple Sclerosis

Three prior studies examined LICI in the context of Multiple Sclerosis (MS) (Table 11). In a cross-sectional study, Mori et al. examined the correlation between disability scores and LICI levels in MS patients, and the study did not result in any significant findings for LICI (133). Nantes et al. compared MS patients with HC and examined the association between LICI and cortical damage measured with MRI. It was demonstrated that LICI did not differ across relapsing-remitting MS patients, progressive MS patients, and HC. Additionally, there was no significant correlation between measures of cortical damage and LICI in MS patients (134). Squintani et al. investigated the role of LICI in the improvement of spastic hypertonia in MS patients following 9-tetrahydrocannabinol and cannabidiol (THC: CBD) oromucosal spray treatment. The results showed that LICI impairment in MS patients relative to HC (*p* < 0.05) significantly improved after THC: CBD treatment for 4 weeks (*p* < 0.05) (135).

**Table 11:** LICI in patients with multiple sclerosis.

**References**	**Subjects**	**Method**	**ISI**	**LICI**
Mori et al. (133)	89 MS	TMS-EEG	100 ms	Comparison not measured
Nantes et al. (134)	36 MS, 18 HC	TMS-EMG	100 ms	↔
Squintani et al. (135)	19 MS, 19 HC	TMS-EMG	100 ms	↓

*ISI, Interstimulus interval; MS, Multiple Sclerosis; LICI, Long-interval intracortical inhibition; HC, Healthy controls. ↓: decreased, ↔: no significant difference*.

Findings have varied with respect to multiple sclerosis. The dysfunctional GABAergic transmission and cortical inhibition in MS patients have been demonstrated in the literature and baclofen, which acts on GABA_B_ receptors, is known as a reliable agent in treating spasticity, which is a common debilitating symptom in patients with MS (136, 137). The study included in our review presented significant LICI deficit in MS patients with treatment resistant spasticity (135). Therefore, stratified analysis, according to spasticity, might be required to reveal the association between LICI and MS symptoms.

#### LICI in Patients With Stroke

LICI in stroke patients was studied in 6 studies (Table 12). LICI and its evolvement over time were examined in post-stroke patients by Swayne et al. Results demonstrated no significant change in LICI over time measured at 1, 3, and 6 months following stroke. LICI in the affected hemisphere was reduced in the patient group relative to healthy controls (*p* = 0.029), and it was correlated with poorer clinical scores in the acute period and 3 months post-stroke but not at 6 months (138). In a cross-sectional study, Kuppuswamy et al. investigated the association between post-stroke fatigue and LICI and did not demonstrate any significant relation (139). Schambra et al. failed to demonstrate any significant LICI difference across acute and chronic stroke patients and controls (140). In a double-blinded placebo-controlled randomized cross over study, the same group examined the effect of theophylline treatment on LICI in 18 chronic stroke patients. There was no significant LICI change in the theophylline group relative to placebo in chronic stroke patients (141). Mooney et al. compared chronic stroke patients and HC based on LICI and investigated its correlation with GABA concentration measured with magnetic resonance spectroscopy. LICI was found to be enhanced in chronic stroke patients relative to HC in the ipsilesional motor cortex (*p* <0.001), whereas there was no significant association between LICI and metabolite concentrations in stroke patients and HC (142). Another study by the same group confirmed the same finding by demonstrating greater LICI in the ipsilesional motor cortex of chronic stroke patients relative to HC at the ISI of 150 ms and indicated that LICI did not change significantly following motor skill learning task in both chronic stroke patients and HC (143).

**Table 12:** LICI in patients with stroke.

**References**	**Subjects**	**Method**	**ISI**	**LICI**
Swayne et al. (138)	10 stroke patients, 10 HC	TMS-EMG	100 ms	↓
Kuppuswamy et al. (139)	70 stroke patients	TMS-EMG	100 ms	Comparison not measured
Schambra et al. (140)	41 stroke patients, 21 HC	TMS-EMG	100 ms	↔
Schambra et al. (141)	18 stroke patients	TMS-EMG	150–250 ms	Comparison not measured
Mooney et al. (142)	12 stroke patients, 16 HC	TMS-EEG	100 ms	↑
Mooney et al. (143)	10 stroke patients, 12 HC	TMS-EMG	100, 150	↑

*ISI, Interstimulus interval; HC, Healthy controls; LICI, Long-interval intracortical inhibition. ↑: increased, ↓: decreased, ↔: no significant difference.*

Studies have presented mixed results in stroke. Studies that found alterations in LICI discussed the methodological differences (using single ISI vs. using range of ISI) as an explanation for the discrepancy between studies (142). On the other hand, it has been consistently shown that LICI has not changed after interventions. Animal studies demonstrated the importance of GABA_B_ mediated inhibitory transmission in post-stroke recovery (144). Baclofen was also shown to be effective compared to conventional medical management in increasing quality of life in patients with post-stroke spasticity (145). Stratified analysis of patients, according to spasticity, might reveal differences in LICI between patients with a history of stroke and healthy controls.

#### LICI in Patients With Traumatic Brain Injury

Most prior studies examining LICI alteration after traumatic brain injury (TBI) focused on the motor cortex and used EMG measures except for one prior TMS-EEG study (Table 13). Tremblay et al. investigated LICI in 12 football players with concussion history that occurred more than a year ago and 14 non-concussed players. The findings demonstrated that athletes with concussion history had a significantly enhanced LICI relative to non-concussed (*p* = 0.05) (146). De Beaumont et al. confirmed the same finding showing enhanced LICI in concussed football players (*p* < 0.03), which was found to be correlated with the number of previous concussions (*p* < 0.05) (147). Another study by De Beaumont et al. examined the effect of LICI on synaptic plasticity, measured with paired associative stimulation (PAS) inducing long-term potentiation (LTP)/ long-term depression (LTD) effects in concussed football players and non-concussed control groups. Results indicated enhanced LICI in the concussed group at baseline, which was correlated with suppressed synaptic plasticity (*p* = 0.037) (148). A study including 40 retired concussed Australian football players and 20 HC, presented contradictory results relative to previous findings indicating that concussed athletes had reduced LICI relative to HC (*p* > 0.001). Additionally, reduction in LICI was associated with poorer performance in finer motor dexterity (*p* = 0.049) (149). Tremblay et al. assessed LICI and its association with metabolic disruption after TBI, ^1^H-MRS. Unlike previous studies, results demonstrated that there was no significant LICI difference in concussed players relative to non-concussed. Nevertheless, GABA levels measured with ^1^H-MRS were positively correlated with LICI in concussed players (*p* = 0.001) (150). Another study investigating cortical inhibition in concussed football players examined LICI during the acute asymptomatic phase following concussion (1–4 weeks after), and results indicated that there was no significant LICI alteration in concussed players relative to non-concussed (151). Lewis et al. compared retired elite rugby players, community-level rugby players, and non-contact sport players as controls and found that LICI was enhanced in elite players relative to controls, whereas there was no significant difference between community-level players and controls (152). Seeger et al. evaluated children 4-weeks after mild TBI (mTBI), which included 35 symptomatic and 27 asymptomatic subjects, all with mTBI, and 28 HC. Findings indicated that the symptomatic mTBI group had reduced LICI relative to HC, and reduction in LICI was associated with increased post-concussion symptom severity (*p* = 0.027, 0.012). This study was different from previous TBI studies as it contained both female and male subjects, and results demonstrated that females had more pronounced LICI (*p* = −0.016) (153). A cross-sectional study by Pearce et al. showed reduced LICI in concussed rugby players, 15–21 years after the injury, relative to HC, and LICI alteration was associated with slower motor dexterity (*p* = 0.03, *p* < 0.01) (154). In another study by Pearce et al. LICI alteration in post-concussion syndrome (PCS) was investigated evaluating 20 concussed subjects with PCS, 20 asymptomatic subjects with a history of concussion, and 20 HC. Results indicated that LICI was enhanced in the PCS group relative to recovered subjects and controls (*p* < 0.001); furthermore, worsened fatigue and poorer amplitude discrimination was associated with altered LICI (*p* < 0.001, 0.02) (155). A prospective cohort study by King et al. involving 78 children with persistent post-concussive symptoms, 29 asymptomatic with TBI history, and 26 age and gender-matched HC, examined LICI alteration 1 and 2 months post-injury and its association with the persistence of symptoms. It was shown that LICI did not differ across groups at 1 and 2 months post-injury; likewise, it did not significantly change over time (157). Opie et al. examined LICI in the motor cortex of adult subjects with history of mTBI and HC using both EMG and EEG. Results demonstrated that LICI measured with EMG was enhanced in subjects with a history of mTBI relative to healthy controls (*p* < 0.0001) whereas TMS-EEG measures for LICI did not significantly differ across groups (156).

**Table 13:** LICI in patients with traumatic brain injury.

**References**	**Subjects**	**Method**	**ISI**	**LICI**
Tremblay et al. (146)	12 concussed, 14 HC	TMS-EMG	100 ms	↑
De Beaumont et al. (147)	21 concussed, 15 HC	TMS-EMG	100 ms	↑
De Beaumont et al. (148)	13 concussed, 19 HC	TMS-EMG	100 ms	↑
Pearce et al. (149)	40 concussed, 20 HC	TMS-EMG	100 ms	↓
Tremblay et al. (150)	16 concussed, 14 HC	TMS-EMG	100 ms	↔
Powers et al. (151)	8 concussed, 8 HC	TMS-EMG	100 ms	↔
Lewis et al. (152)	51 concussed, 22 HC 62 with TBI, 22 HC	TMS-EMG	99 ms	↑ in elite players, ↔ in community players ↓ in symptomatic patients
Seeger et al. (153)	25 concussed, 25 HC	TMS-EMG	100 ms	↓
Pearce et al. (154)	20 PCS, 20 recovered concussed, 20 HC	TMS-EMG	100 ms	↑ in PCS patients, ↔ in recovered patients
Pearce et al. (155)	17 TBI, 15 HC	TMS-EMG	100 ms	↑ measured with EMG, ↔ measured with EEG
Opie et al. (156)	78 PPCS, 29 asymptomatic TBI, 26 HC	TMS-EEG	100 ms	↔
King et al. (157)	12 concussed, 14 HC	TMS-EMG	100 ms	↑

*ISI, Interstimulus interval; TBI, traumatic brain injury; LICI, Long-interval intracortical inhibition; PCS, Post-concussion syndrome; HC, Healthy controls; PPCS, Persistent post-concussive symptoms. ↑: increased, ↓: decreased, ↔: no significant difference*.

The majority of the studies have shown enhanced LICI in groups with a history of concussion compared to healthy controls or non-concussed athletes. However, several studies showed reduction in LICI in patients with a history of concussion. Authors discussed that the etiology of the trauma (American football vs. Australian football), number of concussions, and severity of the injury might be confounding the results (149). Excessive GABAergic activity that occurs after a concussion is thought to be a protective mechanism against the excessive glutamatergic activity, which is thought to be an initial response to brain injury (146). Nevertheless, animal studies pointed out that excess GABAergic inhibition might be responsible for TBI instead (158). Therefore, regardless of the timeline after the trauma, excessive GABAergic activity seemed to be associated with TBI.

#### LICI in Patients With Other Neurological Disorders

Two prior studies examined LICI paradigms in patients with Amyotrophic Lateral Sclerosis (ALS). Salerno et al. demonstrated that LICI was reduced in bulbar ALS (*p* = 0.01) and spinal ALS (*p* = 0.02) relative to HC at the ISI of 155 ms (159) (Table 14). However, the difference between the two ALS groups was not significant. Zanette et al. confirmed the same results showing reduced LICI in ALS patients (*p* < 0.01), specifically in those with upper motor neuron involvement (*p* < 0.05) (160). Tamburin et al. studied ataxic patients with pure cerebellar syndrome and demonstrated that LICI was enhanced in the patient group relative to HC at ISI of 200–500 ms (*p* = 0.007) (161). In a cross-sectional study, Kang et al. revealed that LICI was reduced in drug naïve paroxysmal kinesigenic dyskinesia patients relative to HC (162). Patients with migraines were studied by Siniatchkin et al. and results showed no significant LICI alteration in migraine without aura patients (163). Cosentino et al. indicated that there was a correlation between impaired LICI measured with test stimulus of 150% resting motor threshold and increased migraine disease duration (165). Canafoglia et al. investigated LICI in genetically different progressive myoclonus epilepsy syndromes. They found that LICI was significantly impaired in Lafora Body Disease (LBD) relative to HC at ISI of 80–100 ms, but the LICI difference between LBD and Unverricht-Lundborg Disease was not significant (164).

**Table 14:** LICI in patients with other neurologic disorders.

**References**	**Subjects**	**Method**	**ISI**	**LICI**
Salerno et al. (159)	21 ALS, 12 HC	TMS-EMG	55–255 ms	↓
Zanette et al. (160)	35 ALS, HC	TMS-EMG	50–300 ms	↓
Tamburin et al. (161)	8 cerebellar syndrome, 14 HC	TMS-EMG	30–500 ms	↑
Kang et al. (162)	12 PKD, 10 HC	TMS-EMG	80 ms	↓
Siniatchkin et al. (163)	16 migraine, 15 HC	TMS-EEG	60–120 ms	↔
Canafoglia et al. (164)	10 ULD, 5 LBD, 16 HC	TMS-EMG	30–100 ms	↓ in LBD patients
Cosentino et al. (165)	24 migraine, 24 HC	TMS-EMG	100 ms	Comparison not measured

*ISI, Interstimulus interval; PKD, Paroxysmal kinesigenic dyskinesia; LICI, Long-interval intracortical inhibition; ULD, Unverricht-Lundborg Disease (ULD); ALS, Amyotrophic lateral sclerosis; LBD, Lafora body disease; HC, Healthy controls. ↑: increased, ↓: decreased, ↔: no significant difference*.

## Discussion

The search for biomarkers in neuropsychiatric disorders spans several decades and arguably has progressed slower for psychiatric disorders as compared to neurological disorders. The interest for ongoing research in neuropsychiatric biomarkers is catalyzed by concern for poor clinical outcomes, enhanced diagnostics, and interventional development. Descriptive diagnostic approaches to psychiatric disorders are necessary clinical realities that often fail to provide valid neurophysiological constructs of disease. In general, psychiatric research is plagued by variable methodologies, meager effect sizes, and limited replications.

This was the first systematic review of LICI as a putative biomarker in neuropsychiatric disorders. Broadly, present LICI findings are somewhat mixed and not disease specific. Impairments of LICI have been demonstrated in ADHD, depression, schizophrenia, epilepsy, ALS, and dementia. There were mixed and inconsistent findings in bipolar disorder, neurodevelopment disorders, substance use disorders, multiple sclerosis, stroke, and TBI. Among the studies, LICI has been investigated as a diagnostic and prognostic biomarker, a predictor of treatment response, and a marker of symptom severity. Few studies investigated the reproducibility of LICI. It is also important to highlight that many of the studies focused on bipolar disorder, depression, schizophrenia, dementia, epilepsy, and TBI had overlapping samples among separate manuscripts (36, 37, 39–42, 57, 79, 80, 85, 97, 99). This creates additional challenges in considering the validity, reliability, and synthesis of existing neuropsychiatric LICI literature.

Impairment in LICI in neuropsychiatric diseases has been mostly demonstrated in the direction of reduced cortical inhibition, however, increased cortical inhibition (increased LICI) has also been shown, especially in bipolar disorder, TBI, and Parkinson's disease. It is possible that the disruption of the networks directly associated with the inhibitory GABAergic activity results in reduced LICI whereas, an insult to the networks associated with increased excitatory activity leads to a compensatory increase in GABAergic activity resulting in increased cortical inhibition (increased LICI). Even though there is not sufficient evidence to suggest whether the disruption of inhibitory/excitatory balance in neuropsychiatric disorders is state or trait dependent, several studies have shown restoration of LICI following symptoms remission (93, 155). It is important to highlight a substantial limitation of the present review. A number of studies that were included examined the response to paired-pulse stimulation with ISIs of 200–300 ms and were referred to as LICI. However, work by Cash et al. suggests that stimulation with ISIs at these durations produces a period of late cortical disinhibition that is distinct from LICI (166). These studies were included as the intent was to provide and exhaustive review of prior work with LICI and many of the studies included measurements with ISIs above and below 200 ms. This is an important confound in interpreting the results and could explain some of the broad discrepant findings.

Broadly, TMS-EMG and TMS-EEG measures of LICI are appealing from a practical standpoint. Cortical inhibition measures with TMS are relatively inexpensive, easy to collect, straight forward to analyze, and have demonstrated high test-retest reliability. The prior work focused on LICI has important methodologic limitations to consider for future studies. Medication regimens in clinical populations must be carefully considered with respect to both safety and as confounds. These factors must be characterized and accounted for in future research. When ethically and pragmatically feasible, medicated and unmedicated patient populations should be tested. Disease progression or staging should be carefully described in future studies. Methodology to standardize TMS coil orientation and stimulus intensity is an important future consideration. Electrical field modeling and stereotactic neuronavigation are invaluable tools in establishing reliable study protocols. Further considerations include an online inspection of MEP or TMS-evoked potential data to monitor for artifact and signal-to-noise ratio. Standardized pre-processing and post-processing methodology with detailed published descriptions are additional considerations. Studies with TMS-EEG present unique challenges as recent work has advocated for careful peripheral sensory controls as peripheral effects may have presented confounds in prior TMS-EEG work. Experts have advocated for standardized approaches, the methodology that controls for peripheral effects, and data sharing.

## Conclusion

Current studies with LICI have methodologic weaknesses and discordant findings. Future study, with rigorous methodology is needed to develop LICI paradigms for risk assessment, screening, diagnosis, prognosis, and monitoring treatment effects in neuropsychiatric disorders. With further work, measures of LICI could be rapidly translated into clinical settings.

## Data Availability Statement

The original contributions presented in the study are included in the article/Supplementary Material, further inquiries can be directed to the corresponding author/s.

## Author Contributions

All authors made contributions to the conception and design of the study, assisted with the acquisition and review of articles, contributed to the interpretation of findings, drafted the article, assisted with revisions, and approved the final version of the manuscript prior to submission.

## Conflict of Interest

PC has received research support from Neuronetics, NeoSync, and Pfizer. He has received in-kind support from AssureRx, MagVenture, and Neuronetics. He has provided consultation for Myriad Genetics Procter & Gamble Co., and Sunovion. JV has received in-kind support from AssureRx. The remaining authors declare that the research was conducted in the absence of any commercial or financial relationships that could be construed as a potential conflict of interest.
