# Supplementary material for: A Systematic Review of Long-Interval Intracortical Inhibition as a Biomarker in Neuropsychiatric Disorders
Source: Front Psychiatry. 2021 Jun 2;12:678088. doi: 10.3389/fpsyt.2021.678088 (PMC8206493; doi:10.3389/fpsyt.2021.678088)
Supplement: Supplementary file 1 [file Data_Sheet_1.PDF]

Search Strategy:

EBM Reviews:
(long-interval-cortical-inhibition or long-interval-intracortical-inhibition or lici or long-interval-intra-cortical-inhibition).ab,hw,ti. AND (Affective or Anxiety or Autis* or Bipolar or Borderline or Mental* or Dissociative or Eating-Disorder* or Neuros* or Neurot* or Paraphili* or Personality-Disorder* or Psych* or Somatoform or Addict* or Mood* or Depress* or Seasonal-Affective or Dysthymi* or Hoard* or Cyclothymi* or Mania or Manic or Hypomani* or Melancholi* or Obsessive or Compulsive or OCD or Panic or Phobia* or Trichotillomania or Acrophobi* or Agoraphobi* or Claustrophobi* or Ophidiophobi* or Asperger* or Developmental-Disorder* or Neurodevelopmental-Disorder* or Depersonalization or Fugue or Deliri* or Anorexi* or Binge or Bulimi* or Eating-Disorder* or Hyperphagia or Kleine-Levin or Pica or Attention-Deficit or ADHD or Conduct-Disorder* or Oppositional-Defiant or Disruptive-Behavior* or Exhibitionis* or Fetishis* or Incest* or Pedophil* or Paraphili* or Sadomasochis* or Voyeuris* or Masochis* or Sadis* or Self-Destructive or Self-Injur* or Suicid* or Self-Mutilat* or Automutilat* or (Personality adj1 Disorder*) or Schizo* or Body-Dysmorphic or Conversion-Disorder* or Hypochondria* or Hysteri* or Neurasthenia or Neurodermatitis or Somat* or Neuropsych* or Neurocognitive-Disorder* or Amnesia* or Traumatic-Brain or Traumatic-Encephalopathy or TBI or Tourette*or Alzheimer* or Insan*or Pseudocyesis or Pseudodementia or Dementia or Delusion* or Munchausen or Adjustment-Disorder* or Attachment-Disorder* or Posttraumatic or post-traumatic or PTSD or Disinhibited-Social-Engagement-Disorder or Addict* or Alcohol* or ((Drug or Chemical* or Substance) adj1 (Abuse or Dependen*)) or Opioid* or Cannabis or Cocaine or Nicotine* or Tobacco or Smok* or Stroke* or Transient-Ischemic-Attack* or Transient-Ischaemic-Attack or TIA or cerebrovascular-accident* or Epilep* or Fragile-X or (martin adj1 bell) or (gillian adj1 turner) or dystoni* or Dyskinesia* or Parkinson* or Lewy-body or prion-disease* or transmissible-spongiform-encephalopath* or Creutzfeldt-Jakob or Movement-disorder* or ALS or amyotrophic-lateral-sclerosis or Lou-Gehrig* or brain-injur* or concussion* or tremor* or Chorea or Huntington* or myoclonus or hemiballis* or ((Corticobasal or Cortical-basal) adj2 degeneration) or (Wilson* adj1 disease) or Progressive-supranuclear-palsy or Multiple-sclerosis or Migraine* or (Frontotemporal adj1 degeneration) or Ataxi* or Tetraplegi* or Quadriplegi* or Developmental-coordination-disorder or dyspraxi* or Encephalopath* or (Rett* adj1 Syndrome) or Encephalitis or extrapyramidal)).ab,hw,ti.

Embase:
(long-interval-cortical-inhibition or long-interval-intracortical-inhibition or lici or long-interval-intra-cortical-inhibition).ab,kw,ti. AND (exp mental disease/ or (Affective or Anxiety or Autis* or Bipolar or Borderline or Mental* or Dissociative or Eating-Disorder* or Neuros* or Neurot* or Paraphili* or Personality-Disorder* or Psych* or Somatoform or Addict* or Mood* or Depress* or Seasonal-Affective or Dysthymi* or Hoard* or Cyclothymi* or Mania or Manic or Hypomani* or Melancholi* or Obsessive or Compulsive or OCD or Panic or Phobia* or Trichotillomania or Acrophobi* or Agoraphobi* or Claustrophobi* or Ophidiophobi* or Asperger* or Developmental-Disorder* or Neurodevelopmental-Disorder* or Depersonalization or Fugue or Deliri* or Anorexi* or Binge or Bulimi* or Eating-Disorder* or Hyperphagia or Kleine-Levin or Pica or Attention-Deficit or ADHD or Conduct-Disorder* or Oppositional-Defiant or Disruptive-Behavior* or Exhibitionis* or Fetishis* or Incest* or Pedophil* or Paraphili* or Sadomasochis* or Voyeuris* or Masochis* or Sadis* or Self-Destructive or Self-Injur* or Suicid* or Self-Mutilat* or Automutilat* or (Personality adj1 Disorder*) or Schizo* or Body-Dysmorphic or Conversion-Disorder* or Hypochondria* or Hysteri* or Neurasthenia or Neurodermatitis or Somat* or Neuropsych* or Neurocognitive-Disorder* or Amnesia* or Traumatic-Brain or Traumatic-Encephalopathy or TBI or Tourette*or Alzheimer* or Insan*or Pseudocyesis or Pseudodementia or Dementia or Delusion* or Munchausen or Adjustment-Disorder* or Attachment-Disorder* or Posttraumatic or post-traumatic or PTSD or Disinhibited-Social-Engagement-Disorder or Addict* or Alcohol* or ((Drug or Chemical* or Substance) adj1 (Abuse or Dependen*)) or Opioid* or Cannabis or Cocaine or Nicotine* or Tobacco or Smok* or exp cerebrovascular accident/ or Stroke* or exp transient ischemic attack/ or Transient-Ischemic-Attack* or Transient-Ischaemic-Attack or TIA or cerebrovascular-accident* or exp Epilepsy/ or Epilep* or Fragile X Syndrome/ or Fragile-X or (martin adj1 bell) or (gillian adj1 turner) or exp dystonia/ or dystoni* or Dyskinesia* or exp Parkinson disease/ or Parkinson* or Lewy body/ or Lewy-body or exp Prion Disease/ or prion-disease* OR transmissible-spongiform-encephalopath* or Creutzfeldt-Jakob or exp motor dysfunction/ or movement-disorder* or Amyotrophic Lateral Sclerosis/ or ALS or amyotrophic-lateral-sclerosis or Lou-Gehrig* or exp Dyskinesia/ or exp Brain Injury/ or brain-injur* or concussion* or tremor*or Chorea or exp extrapyramidal syndrome/ or Huntington* or exp corticobasal degeneration/ or Wilson disease/ or myoclonus or hemiballis* or ((Corticobasal or Cortical-basal) adj2 degeneration) or (Wilson* adj1 disease) or progressive supranuclear palsy/ or Progressive-supranuclear-palsy or exp Multiple Sclerosis/ or multiple-sclerosis or exp Migraine/ or migraine* or (Frontotemporal adj1 degeneration) OR ataxi* or exp Quadriplegia/ or tetraplegi* or quadriplegi* or Motor Skills Disorders/ or developmental-coordination-disorder or exp Brain Disease/ or encephalopath* or Rett Syndrome/ or (Rett* adj1 Syndrome) or exp Encephalitis/ or encephalitis or extrapyramidal).ab,kw,ti.) Limit to English

Ovid MEDLINE(R) 1946 to Present and Epub Ahead of Print, In-Process & Other Non-Indexed Citations and Ovid MEDLINE(R) Daily:
(long-interval-cortical-inhibition or long-interval-intracortical-inhibition or lici or long-interval-intra-cortical-inhibition).ab,kw,ti. AND (exp Mental Disorders/ or (Affective or Anxiety or Autis* or Bipolar or Borderline or Mental* or Dissociative or Eating-Disorder* or Neuros* or Neurot* or Paraphili* or Personality-Disorder* or Psych* or Somatoform or Addict* or Mood* or Depress* or Seasonal-Affective or Dysthymi* or Hoard* or Cyclothymi* or Mania or Manic or Hypomani* or Melancholi* or Obsessive or Compulsive or OCD or Panic or Phobia* or Trichotillomania or Acrophobi* or Agoraphobi* or Claustrophobi* or Ophidiophobi* or Asperger* or Developmental-Disorder* or Neurodevelopmental-Disorder* or Depersonalization or Fugue or Deliri* or Anorexi* or Binge or Bulimi* or Eating-Disorder* or Hyperphagia or Kleine-Levin or Pica or Attention-Deficit or ADHD or Conduct-Disorder* or Oppositional-Defiant or Disruptive-Behavior* or Exhibitionis* or Fetishis* or Incest* or Pedophil* or Paraphili* or

Sadomasochis* or Voyeuris* or Masochis* or Sadis* or Self-Destructive or Self-Injur* or Suicid* or Self-Mutilat* or Automutilat* or (Personality adj1 Disorder*) or Schizo* or Body-Dysmorphic or Conversion-Disorder* or Hypochondria* or Hysteri* or Neurasthenia or Neurodermatitis or Somat* or Neuropsych* or Neurocognitive-Disorder* or Amnesia* or Traumatic-Brain or Traumatic-Encephalopathy or TBI or Tourette*or Alzheimer* or Insan*or Pseudocyesis or Pseudodementia or Dementia or Delusion* or Munchausen or Adjustment-Disorder* or Attachment-Disorder* or Posttraumatic or post-traumatic or PTSD or Disinhibited-Social-Engagement-Disorder or Addict* or Alcohol* or ((Drug or Chemical* or Substance) adj1 (Abuse or Dependen*)) or Opioid* or Cannabis or Cocaine or Nicotine* or Tobacco or Smok* or exp Stroke/ or Stroke* or Ischemic Attack, Transient/ or Transient-Ischemic-Attack* or Transient-Ischaemic-Attack or TIA or cerebrovascular-accident* or exp Epilepsy/ or Epilep* or Fragile X Syndrome/ or Fragile-X or (martin adj1 bell) or (gillian adj1 turner) or dystoni* or Dyskinesia* or exp Parkinsonian Disorders/ or Parkinson* or Lewy-body or exp Prion Diseases/ or prion-disease* OR transmissible-spongiform-encephalopath* or Creutzfeldt-Jakob or exp Movement Disorders/ or movement-disorder* or Amyotrophic Lateral Sclerosis/ or ALS or amyotrophic-lateral-sclerosis or Lou-Gehrig* or exp Dyskinesias/ or exp Brain Injuries/ or brain-injur* or concussion* or tremor*or Chorea or Huntington Disease/ or Huntington* or Hepatolenticular Degeneration/ or myoclonus or hemiballis* or ((Corticobasal or Cortical-basal) adj2 degeneration) or (Wilson* adj1 disease) or Supranuclear Palsy, Progressive/ or Progressive-supranuclear-palsy or exp Multiple Sclerosis/ or multiple-sclerosis or exp Migraine Disorders/ or migraine* or (Frontotemporal adj1 degeneration) OR ataxi* or exp Quadriplegia/ or tetraplegi* or quadriplegi* or Motor Skills Disorders/ or developmental-coordination-disorder or exp Brain Diseases/ or encephalopath* or Rett Syndrome/ or (Rett* adj1 Syndrome) or exp Encephalitis/ or encephalitis or extrapyramidal).ab,kw,ti.) Limit to English

Ovid APA PsycINFO:

(long-interval-cortical-inhibition or long-interval-intracortical-inhibition or lici or long-interval-intra-cortical-inhibition).ab,id,ti. AND (exp mental disorders/ or (Affective or Anxiety or Autis* or Bipolar or Borderline or Mental* or Dissociative or Eating-Disorder* or Neuros* or Neurot* or Paraphili* or Personality-Disorder* or Psych* or Somatoform or Addict* or Mood* or Depress* or Seasonal-Affective or Dysthymi* or Hoard* or Cyclothymi* or Mania or Manic or Hypomani* or Melancholi* or Obsessive or Compulsive or OCD or Panic or Phobia* or Trichotillomania or Acrophobi* or Agoraphobi* or Claustrophobi* or Ophidiophobi* or Asperger* or Developmental-Disorder* or Neurodevelopmental-Disorder* or Depersonalization or Fugue or Deliri* or Anorexi* or Binge or Bulimi* or Eating-Disorder* or Hyperphagia or Kleine-Levin or Pica or Attention-Deficit or ADHD or Conduct-Disorder* or Oppositional-Defiant or Disruptive-Behavior* or Exhibitionis* or Fetishis* or Incest* or Pedophil* or Paraphili* or Sadomasochis* or Voyeuris* or Masochis* or Sadis* or Self-Destructive or Self-Injur* or Suicid* or Self-Mutilat* or Automutilat* or (Personality adj1 Disorder*) or Schizo* or Body-Dysmorphic or Conversion-Disorder* or Hypochondria* or Hysteri* or Neurasthenia or Neurodermatitis or Somat* or Neuropsych* or Neurocognitive-Disorder* or Amnesia* or Traumatic-Brain or Traumatic-Encephalopathy or TBI or Tourette*or Alzheimer* or Insan*or Pseudocyesis or Pseudodementia or Dementia or Delusion* or Munchausen or Adjustment-Disorder* or Attachment-Disorder* or Posttraumatic or post-traumatic or PTSD or Disinhibited-Social-Engagement-Disorder or Addict* or Alcohol* or ((Drug or Chemical* or Substance) adj1 (Abuse or Dependen*)) or Opioid* or Cannabis or Cocaine or Nicotine* or Tobacco or Smok* or exp cerebrovascular accidents/ or Stroke* or Transient-Ischemic-Attack* or Transient-Ischaemic-Attack or TIA or cerebrovascular-accident* or exp epilepsy/ or Epilep* or fragile x syndrome/ or Fragile-X or (martin adj1 bell) or (gillian adj1 turner) or exp neurodegenerative diseases/ or dystoni* or Dyskinesia* or Parkinson* or Lewy-body or prion-disease* or transmissible-spongiform-encephalopath* or Creutzfeldt-Jakob or exp movement disorders/ or Movement-disorder* or ALS or amyotrophic-lateral-sclerosis or Lou-Gehrig* or exp brain injuries/ or brain-injur* or concussion* or tremor* or Chorea or Huntington* or myoclonus or hemiballis* or ((Corticobasal or Cortical-basal) adj2 degeneration) or (Wilson* adj1 disease) or progressive supranuclear palsy/ or Progressive-supranuclear-palsy or multiple sclerosis/ or Multiple-sclerosis or migraine headache/ or Migraine* or (Frontotemporal adj1 degeneration) or Ataxi* or Tetraplegi* or quadriplegia/ or Quadriplegi* or Developmental-coordination-disorder or exp dyspraxia/ or dyspraxi* or exp encephalopathies/ or Encephalopath* or rett syndrome/ or (Rett* adj1 Syndrome) or exp encephalitis/ or Encephalitis or extrapyramidal).ab,id,ti.).ab,id,ti.) Limit to English

Scopus:

(TITLE-ABS-KEY(long-interval-cortical-inhibition OR long-interval-intracortical-inhibition OR lici OR long-interval-intra-cortical-inhibition)) AND ((TITLE-ABS-KEY((personality W/1 disorder*) OR ((drug OR chemical* OR substance) W/1 (abuse OR dependen*)))) OR (TITLE-ABS-KEY(Affective or Anxiety or Autis* or Bipolar or Borderline or Mental* or Dissociative or Eating-Disorder* or Neuros* or Neurot* or Paraphili* or Personality-Disorder* or Psych* or Somatoform or Addict* or Mood* or Depress* or Seasonal-Affective or Dysthymi*)) OR (TITLE-ABS-KEY(Hoard* or Cyclothymi* or Mania or Manic or Hypomani* or Melancholi* or Obsessive or Compulsive or OCD or Panic or Phobia* or Trichotillomania or Acrophobi* or Agoraphobi* or Claustrophobi* or Ophidiophobi* or Asperger* or Developmental-Disorder*)) OR (TITLE-ABS-KEY(Neurodevelopmental-Disorder* or Depersonalization or Fugue or Deliri* or Anorexi* or Binge or Bulimi* or Eating-Disorder* or Hyperphagia or Kleine-Levin or Pica or Attention-Deficit or ADHD)) OR (TITLE-ABS-KEY(Conduct-Disorder* or Oppositional-Defiant or Disruptive-Behavior* or Exhibitionis* or Fetishis* or Incest* or Pedophil* or Paraphili* or Sadomasochis* or Voyeuris* or Masochis* or Sadis* or Self-Destructive or Self-Injur* or Suicid* or Self-Mutilat*)) OR (TITLE-ABS-KEY(Automutilat* or Schizo* or Body-Dysmorphic or Conversion-Disorder* or Hypochondria* or Hysteri* or Neurasthenia or Neurodermatitis or Somat* or Neuropsych* or Neurocognitive-Disorder* or Amnesia* or Traumatic-Brain or Traumatic-Encephalopathy or TBI)) OR (TITLE-ABS-KEY(Tourette* or Alzheimer* or Insan* or Pseudocyesis or Pseudodementia or Dementia or Delusion* or Munchausen or Adjustment-Disorder* or Attachment-Disorder* or Posttraumatic or post-traumatic or PTSD or Disinhibited-Social-Engagement-Disorder or Addict*)) OR (TITLE-ABS-KEY(Alcohol* or Opioid* or Cannabis or Cocaine or Nicotine* or Tobacco or Smok* OR Stroke* or Transient-Ischemic-Attack* or Transient-Ischaemic-Attack or TIA or cerebrovascular-accident* or Epilep* or Fragile-X or (martin W/1 bell) or (gillian W/1 turner) or dystoni* or Dyskinesia* or Parkinson* or Lewy-body or prion-disease* or transmissible-spongiform-encephalopath* or Creutzfeldt-Jakob or Movement-disorder* or ALS or amyotrophic-lateral-sclerosis or Lou-Gehrig* or brain-injur* or concussion* or tremor* or Chorea or Huntington* or myoclonus or hemiballis* or ((Corticobasal or Cortical-basal) W/2 degeneration) or (Wilson* W/1 disease)

or Progressive-supranuclear-palsy or Multiple-sclerosis or Migraine* or (Frontotemporal W/1 degeneration) or Ataxi* or Tetraplegi* or Quadriplegi* or Developmental-coordination-disorder or dyspraxi* or Encephalopath* or (Rett* W/1 Syndrome) or Encephalitis or extrapyramidal))) AND (LIMIT-TO (LANGUAGE,"English"))

Web of Science:
TS=(long-interval-cortical-inhibition or long-interval-intracortical-inhibition or lici or long-interval-intra-cortical-inhibition) AND TS=(Affective or Anxiety or Autis* or Bipolar or Borderline or Mental* or Dissociative or Eating-Disorder* or Neuros* or Neurot* or Paraphili* or Personality-Disorder* or Psych* or Somatoform or Addict* or Mood* or Depress* or Seasonal-Affective or Dysthymi* or Hoard* or Cyclothymi* or Mania or Manic or Hypomani* or Melancholi* or Obsessive or Compulsive or OCD or Panic or Phobia* or Trichotillomania or Acrophobi* or Agoraphobi* or Claustrophobi* or Ophidiophobi* or Asperger* or Developmental-Disorder* or Neurodevelopmental-Disorder* or Depersonalization or Fugue or Deliri* or Anorexi* or Binge or Bulimi* or Eating-Disorder* or Hyperphagia or Kleine-Levin or Pica or Attention-Deficit or ADHD or Conduct-Disorder* or Oppositional-Defiant or Disruptive-Behavior* or Exhibitionis* or Fetishis* or Incest* or Pedophil* or Paraphili* or Sadomasochis* or Voyeuris* or Masochis* or Sadis* or Self-Destructive or Self-Injur* or Suicid* or Self-Mutilat* or Automutilat* or (Personality NEAR/1 Disorder*) or Schizo* or Body-Dysmorphic or Conversion-Disorder* or Hypochondria* or Hysteri* or Neurasthenia or Neurodermatitis or Somat* or Neuropsych* or Neurocognitive-Disorder* or Amnesia* or Traumatic-Brain or Traumatic-Encephalopathy or TBI or Tourette*or Alzheimer* or Insan*or Pseudocyesis or Pseudodementia or Dementia or Delusion* or Munchausen or Adjustment-Disorder* or Attachment-Disorder* or Posttraumatic or post-traumatic or PTSD or Disinhibited-Social-Engagement-Disorder or Addict* or Alcohol* or ((Drug or Chemical* or Substance) NEAR/1 (Abuse or Dependen*)) or Opioid* or Cannabis or Cocaine or Nicotine* or Tobacco or Smok* or Stroke* or Transient-Ischemic-Attack* or Transient-Ischaemic-Attack or TIA or cerebrovascular-accident* or Epilep* or Fragile-X or (martin NEAR/1 bell) or (gillian NEAR/1 turner) or dystoni* or Dyskinesia* or Parkinson* or Lewy-body or prion-disease* or transmissible-spongiform-encephalopath* or Creutzfeldt-Jakob or Movement-disorder* or ALS or amyotrophic-lateral-sclerosis or Lou-Gehrig* or brain-injur* or concussion* or tremor* or Chorea or Huntington* or myoclonus or hemiballis* or ((Corticobasal or Cortical-basal) NEAR/2 degeneration) or (Wilson* NEAR/1 disease) or Progressive-supranuclear-palsy or Multiple-sclerosis or Migraine* or (Frontotemporal NEAR/1 degeneration) or Ataxi* or Tetraplegi* or Quadriplegi* or Developmental-coordination-disorder or dyspraxi* or Encephalopath* or (Rett* NEAR/1 Syndrome) or Encephalitis or extrapyramidal))) Limit to English

Supplementary Table 1 LICI in ADHD patients

Reference	Study Description	Findings
Buchmann, et al. 2007	Design: Clinical trial<br>Participants: Total of 36 participants, 18 ADHD children, 18 HC<br>*Age (yrs):* ADHD: 11 ± 1.91, HC: 11 ± 2<br>*Sex:* ADHD: 15M/3F HC: 15M/3F<br>Measures: TMS administered to left motor cortex, EMG data collected from right FDI. CS was set to produce MEP of 1.0 mV, TS was set to produce MEP 1.0 mV. ISI: 100, 200, 300 ms.<br>Major outcome: LICI in young ADHD patients<br>Additional outcome: Modulation of LICI with methylphenidate treatment	Reduced LICI was identified in drug naïve ADHD children at 100 ms interval. ($p=0.001$). Methylphenidate administration improved LICI significantly yielding results similar to the control group. ($p<0.001$)<br>Reduction in Conners score following methylphenidate treatment was correlated with LICI restoration ($p<0.05$).
Hoeppner, et al. 2008	Design: Cross-sectional<br>Participants: Total of 42 participants, 21 drug naïve ADHD, 21 HC<br>*Age (yrs):* ADHD: 28.9 ± 9.2, HC: 29.4 ± 9.3<br>*Sex:* ADHD: 9F/12M, HC: 9F/12M<br>Measures: TMS administered to left motor cortex, EMG data collected from right FDI. CS was set to produce MEP of 1.0 mV, TS was set to produce MEP 1.0 mV. ISI: 100, 200, 300 ms. Conners scale was used to evaluate hyperactive behavior.<br>Major outcome: LICI deficit in adult ADHD patients	There was no significant inhibitory deficit in the adult ADHD group. (ns)

ADHD: Attention deficit hyperactivity disorder
HC: Healthy controls
TMS: Transcranial magnetic stimulation
EMG: Electromyography
MEP: Mean evoked potential
FDI: First dorsal interosseous
CS: Conditioning stimulus
TS: Test stimulus
ISI: Interstimulus interval
LICI: Long-interval intracortical inhibition

Supplementary Table 2 LICI and Bipolar Disorder patients

Reference	Study Description	Findings
Ruiz-Veguilla, et al. 2016	Design: Cohort<br>Participants: 47 Total participants: 19 Bipolar Disorder patients, 28 HC Follow up assessment performed in 15 Bipolar patients.<br>*Age (yrs):* Bipolar: 35.5 ± 11.4, HC: 33.1 ± 7.0<br>*Sex:* Bipolar: 12M/7F, HC: 18M/10F<br>Measures: TMS administered to left motor cortex, EMG data collected from right FDI. CS was set to 120% RMT, TS was set to produce MEP of 1.0 mV ISI: 100, 150, or 250 ms. Protocol was repeated 3 months later.<br>Major outcome: LICI during acute manic phase and after remission in bipolar patients	There was no significant LICI difference between bipolar and control group at baseline.<br>Follow up assessments showed no significant effect of episode on LICI in bipolar patients.
Basavaraju, et al. 2017	Design: Cross-sectional<br>Participants: Total of 112 participants: 39 Manic episode medication-naïve, 28 Remitted first episode manic, 45 HC<br>*Age (yrs):* Manic patients: 32.82 ± 11.01, Remitted mania: 26.86 ± 7.12, HC: 30.68 ± 9.57<br>*Sex:* Manic Bipolar: 17F/22M, Remitted Bipolar: 10F/18M, HC: 22F/23M<br>Measures: TMS administered to left motor cortex, EMG data collected from right FDI. CS was set to produce MEP of 1.0 mV, TS was set to produce MEP 1.0 mV. ISI: 100 ms. Symptom severity was measured with Young's Mania Rating Scale (YMRS).<br>Major outcome: LICI in manic and remitted first episode mania patients	LICI was enhanced in manic medication-naïve BD ($p= 0.021$) patients relative to HC.<br>LICI difference between the remitted first episode mania patients and HC was non-significant.<br>The difference between remitted patients and manic patients was non-significant.<br>There was no significant correlation between symptom severity and LICI levels.
Basavaraju, et al. 2018	Design: Cross-sectional<br>Participants: Total of 84 participants: 39 medication free manic patients, 45 HC<br>*Age (yrs):* Manic patients: 32.82 ± 11.01, HC: 30.68 ± 9.57<br>*Sex:* Manic patients: 17F/22M, HC: 22F/23M<br>Measures: TMS administered to left motor cortex, EMG data collected from right FDI. CS was set to produce MEP of 1.0 mV, TS was set to produce MEP 1.0 mV. ISI: 100 ms. To assess the Mirror neuron system activity (MNA), measurements were done while participants observed goal directed action. Symptom severity was measured with YMRS.<br>Major outcome: MNA, using LICI, in bipolar patients<br>Additional outcome: MNA correlation with symptom severity	LICI was higher in BD patients relative to HC at rest. ($P= 0.026$)<br>Enhanced MNA, using LICI was, was observed in the patient group ($p=0.033$).<br>LICI mediated putative MNA found to have significant correlation with symptom severity. ($p=0.038$)

HC: Healthy controls
TMS: Transcranial magnetic stimulation
EMG: Electromyography
RMT: Resting motor threshold
MEP: Mean evoked potential
FDI: First dorsal interosseous
CS: Conditioning stimulus
TS: Test stimulus
ISI: Interstimulus interval
LICI: Long-interval intracortical inhibition
YMRS: Young's Mania Rating Scale
MNA: Mirror Neuron System Activity

Supplementary Table 3 LICI in patients with Depression

Reference	Study Description	Findings
Croarkin, et al. 2014	Design: Cross-sectional<br>Participants: 16 participants with MDD: 8 treatment responders, 8 treatment nonresponders<br>*Age (yrs):* Responders: 14.1 ± 2.8, Nonresponders: 13.1 ± 1.6<br>*Sex:* Responders: 2M/6F, Nonresponders: 5M/3F<br>Measures: TMS administered to motor cortex, EMG data collected from contralateral APB. CS was set to produce MEP of 0.5-1.5 mV, TS was set to produce MEP of 0.5-1.5 mV ISI: 100, 150, or 200 ms. Patients were receiving Fluoxetine treatment.<br>Major outcome: Pretreatment LICI and treatment response in patients with depression	Treatment-resistant patients showed greater pretreatment LICI deficits (reduced) relative to treatment responders at all ISI (p=0.01, 0.03, 0.01).<br>The relationship was stronger measuring for the left motor cortex.
Croarkin, et al. 2014b	Design: Cross-sectional<br>Participants: 46 Participants, 24 MDD, 22 HC. 33 completed LICI protocol, 14 MDD 19 HC.<br>*Age (yrs):* MDD group: 14 ± 2.1 HC: 13.9 ± 2.2<br>*Sex:* MDD group: 8F/6M HC: 11F/8M<br>Measures: TMS administered to motor cortex, EMG data collected from contralateral APB. CS was set to produce MEP of 0.5-1.5 mV, TS was set to produce MEP of 0.5-1.5 mV. ISI: 100, 150, or 200 ms<br>Major outcome: Association of LICI with age in depressed patients	In the sample of all participants, LICI-200 had a significant negative relationship with age in both right (p=0.05) and left hemisphere (p=0.002).<br>Lowered LICI-200 mean value (enhanced inhibition) with age was observed for depressed group in both right (p= 0.034) and left hemisphere (0.002).<br>LICI and age association in other interstimulus intervals was non-significant.<br>No significant association was observed in the control group.
Sun, et al. 2016	Design: Open label clinical trial<br>Participants: 33 TRD participants, 27 of them had data available for study<br>*Age (yrs):* 46.0 ± 15.3 *Sex:* 12 M/15F<br>Measures: TMS administered to the left DLPFC and motor cortex, data recorded with EEG. CS was set to produce MEP of 1.0 mV, TS was set to produce MEP 1.0 mV. ISI: 100 ms. Baseline measurements were conducted 1 week before MST treatment. Suicidal ideation was measured with Scale for Suicide Ideation (SSI).<br>Major outcome: Association between pre-MST LICI levels and remission of suicidality following MST	SSI score reduction following MST was correlated with greater LICI in DLPFC at baseline (p=0.02).<br>Pretreatment LICI in the motor cortex and change in suicidal ideation following MST treatment were not correlated significantly.

Sun, et al. 2018	Design: Open label clinical trial<br>Participants: 23 TRD<br>*Age (yrs):* 45 ± 12.2. *Sex:* 11M, 12F<br>Measures: TMS administered to the left DLPFC and motor cortex, data recorded with EEG. CS was set to produce MEP of 1.0 mV, TS produce MEP 1.0 mV. ISI: 100 ms. Suicidality was measured with Scale for Suicide Ideation (SSI).<br>Major outcome: Changes in neuroplasticity and cortical inhibition, measured with LICI, and suicidal ideation after MST	LICI in the DLPFC was reduced after MST treatment in patients with resolved suicidal ideation. (p=0.048)<br>No significant LICI change was detected when patients were limited to those without SI remission.<br>LICI reduction was correlated with the SSI change following MST in DLPFC. (p=0.044).<br>No significant LICI alteration was observed in the motor cortex.
Lewis, et al. 2018	Design: Cross-sectional<br>Participants: Total of 74 participants: 37 depressed, 17 depressed + suicidal behavior, 20 HC<br>*Age (yrs):* Depressed: 15.70 ± 1.88, Depressed + SB: 15.88 ± 1.57, HC: 14.20 ± 1.76<br>*Sex:* Depressed:24F/13M, Depressed + SB: 11F/6M, HC: 9F/ 11M<br>Measures: TMS administered to left motor cortex, EMG data collected from right APB. CS was set to produce MEP of 1.0 mV, TS was set to produce MEP 1.0 mV. ISI: 100, 150, 200 ms. Depressive symptom rating measured by HDRS-17. SI was measured with C-SSRS.<br>Major outcome: LICI deficit in depressed individuals with and without SB<br>Additional outcome: Suicidal severity and LICI correlation	Depressed + SB patients exhibited reduced LICI (ISI: 100, 150 ms) relative to HC (p=0.0002, 0.0009) and depressed w/o SB (p= 0.0049, 0.0418).<br>Greater suicidal severity is correlated with greater LICI deficit at ISI of 100, 150 ms (p= 0.0123, 0.0102).
Jeng, et al. 2019	Design: Clinical trial<br>Participants: Total of 72 participants, 36 with MDD: 20 TRD + 16 non-TRD, 36 HC<br>*Age (yrs):* TRD: 42.5 ± 1.3, non-TRD: 42.9 ± 2.6, HC: 41.3 ± 1.8<br>*Sex:* TRD: 9M/11F, non-TRD: 8M/8F, HC: 14M/ 22F<br>Measures: TMS administered to motor cortex, EMG data collected from contralateral APB. CS 120% of RMT mV, TS 120% of RMT. ISI: 100, 200 ms. Depressive symptom rating was measured with HDRS-17.<br>Major outcome: LICI deficit in treatment resistant MDD and treatment responsive MDD patients. Non-TRD patients were receiving SSRIs.<br>Additional outcome: Modulation of LICI following SSRI treatment	TRD patients had markedly reduced LICI compared to non-TRD or HC. (p<0.001)<br>LICI was reduced in non-TRD after 3 months of SSRI treatment. (p = 0.002)<br>Lower LICI was correlated with higher HDRS-17 scores. (p < 0.001)

Lewis, et al. 2019	Design: Cohort<br>Participants: Total 10 depressed participant<br>*Age (yrs):* 15.50 ± 1.18 *Sex:* 6F/4M<br>Measures: TMS administered to left motor cortex, EMG data collected from right APB. CS was set to produce MEP of 1.0 mV, TS was set to produce MEP 1.0 mV. ISI: 100, 150 ms. LICI measured at baseline at 8 weeks after treatment. SI was measured with C-SSRS. Depression severity was measured with CDRS-R.<br>Major outcome: Association between LICI alteration and SI change following antidepressant treatment	Decrease in suicidal ideation is correlated with increase in cortical inhibition (LICI-100), controlling for depression severity, in depressed adolescents treated with antidepressants (p=0.021)<br>Participants with prior SB had reduced follow up LICI-100 than those without history of SB. (p=0.038)
Balzekas, et al. 2019	Design: Cross-sectional<br>Participants: Total of 5 depressed participant<br>*Age (yrs):* 15.5 ± 2.1 *Sex:* 5F<br>Measures: TMS administered to left motor cortex, EMG data collected from right APB. CS was set to produce MEP of 1.0 mV, TS was set to produce MEP 1.0 mV. ISI: 100, 150, 200 ms. Functional connectivity was measured with resting-state functional magnetic resonance imaging (RS-fMRI).<br>Major outcome: Association between RS-fMRI connectivity and LICI	There was no significant correlation between LICI and RS-fMRI connectivity.<br>LICI-200 showed the highest correlation with resting state functional connectivity.
Camsari, et al. 2019	Design: Cohort<br>Participants: Total of 37 participants, 15 MDD, 22 HC. 10 of the MDD returned for follow up assessment.<br>*Age (yrs):* MDD: 15.4 ± 1.2, HC: 13.8 ± 2.2 *Sex:* MDD: 6M/9F, HC: 11M/11F<br>Measures: TMS administered to motor cortex, EMG data collected from contralateral APB. CS was set to produce MEP of 1.0 mV, TS was set to produce MEP 1.0 mV. ISI: 100 ms, 150 ms, 200 ms.<br>Major outcome: Baseline LICI and SSRI post treatment changes in MDD patients	There was no significant LICI difference between groups at baseline.<br>No significant change in LICI was detected following antidepressant treatment.

MDD: Major depressive disorder
HC: Healthy controls
TMS: Transcranial magnetic stimulation
EMG: Electromyography
APB: Abductor Pollicis Brevis
CS: Conditioning stimulus
MEP: Motor evoked potential
TS: Test stimulus
LICI: Long-interval intracortical inhibition
TRD: Treatment resistant depression
HDRS-17: Hamilton depression rating scale
EEG: Electroencephalogram
MST: Magnetic Seizure therapy
SI: Suicidal ideation
RMT: Resting motor threshold
fMRI: Functional Magnetic Resonance Imaging
DLPFC: Dorsolateral Prefrontal cortex
ISI: interstimulus interval
SB: Suicidal behavior
SSRI: Selective serotonin reuptake inhibitor
C-SSRS: Columbia–Suicide Severity Rating Scale
CDRS-R: Children's Depression Rating Scale, Revised

Supplementary Table 4 LICI in Neurodevelopmental disorders patients

Reference	Study Description	Findings
Oberman, et al. 2010	Design: Cross-sectional<br>Participants: Total of 7 participants, 2 Fragile X, 2 ASD, 5 HC<br>*Age (yrs):* FXS: 16,23 ASD: 25,35,44,45,54 HC: 22,27,41,49,54<br>*Sex:* FXS: 2F, ASD: 3M/2F, HC: 2M/3F<br>Measures: TMS administered to left motor cortex, EMG data collected from right FDI. CS was set to 120% RMT, TS was set to 120% RMT. ISI: 100 ms.<br>Major outcome: Intracortical inhibition, measuring LICI, in FXS and ASD patients	No significant LICI difference was found across groups.<br>One of the ASD participants showed enhancement during the LICI paradigm.
Enticott, et al. 2011	Design: Cross-sectional<br>Participants: Total of 70 participants, 36 ASD, 34 HC<br>*Age (yrs):* ASD: 26.00 ±10.48, HC: 26.21 ±6.60<br>*Sex:* ASD: 28/8, HC:23/11<br>Measures: TMS administered to motor cortex, EMG data collected from contralateral FDI. CS was set to 120% RMT, TS was set to 120% RMT. ISI: 100 ms.<br>Major outcome: LICI in ASD individuals	LICI was not significantly affected in both hemispheres in ASD individuals.
Morin-Parent, et al. 2019	Design: Cross-sectional<br>Participants: Total of 36 participants, 18 FXS, 18 HC<br>*Age (yrs):* FXS: 24.88 ± 7.5, HC: 23.88 ± 5.3<br>*Sex:* FXS:16M/2F, HC:16M/2F<br>Measures: TMS administered to motor cortex, EMG data collected from contralateral FDI. CS was set to produce MEP of 1.0 mV, TS was set to produce MEP 1.0 mV. ISI: 100 ms.<br>Major outcome: LICI in individuals with molecular FXS diagnosis	FXS patients showed enhanced LICI. (p= 0.011)<br>When analysis was conducted for only non-medicated FXS individuals versus HC, LICI showed a trend of being enhanced in the FXS group. (p=0.060)
Bernardo, et al. 2020	Design: Cross-sectional<br>Participants: Total of 34 participants,14 patients with typical Rett syndrome, 9 non-RTT epilepsy controls, 11 HC<br>*Age (yrs):* RTT: 22.64 ± 2.12, Epilepsy controls: 25.11 ± 2.56, HC: 22.64 ± 1.75<br>*Sex:* not given<br>Measures: TMS administered to left motor cortex, EMG data collected from right FDI. CS was set to 120% of RMT, TS was set to produce 120% of RMT. ISI: 100 ms, 150 ms. Clinical motor scores were	LICI was reduced in the RTT group relative to HC and epilepsy controls (p=0.002).<br>Lower motor performance measured with CSS and RSGMS motor scales was corelated with reduced LICI (p=0.003).

	measured with clinical severity score (CSS) and the Rett Syndrome Gross Motor Scale (RSGMS).<br>Major outcome: LICI in individuals with Rett syndrome<br>Additional outcome: LICI's correlation with motor scores	

ASD: Autism Spectrum Disorder
HC: Healthy controls
TMS: Transcranial magnetic stimulation
EMG: Electromyography
FDI: First dorsal Introsseus
CS: Conditioning stimulus
RMT: Resting motor threshold
TS: Test stimulus
ISI: Interstimulus interval
MEP: Mean evoked potential
LICI: Long-interval intracortical inhibition
FXS: Fragile X Syndrome
RTT: Rett syndrome

Supplementary Table 5 LICI in Schizophrenia patients

Reference	Study Description	Findings
Fitzgerald, et al. 2003	Design: Cross-sectional<br>Participants: Total of 26 participants, 18 SCZ: 9 medicated + 9 unmedicated, 8 HC<br>*Age (yrs):* Medicated SCZ: 27.7 ± 5.1, Unmedicated SCZ: 33.8 ± 8.2, HC: 29.1 ± 6.0<br>*Sex:* Medicated SCZ: 3F/6M, Unmedicated SCZ: 3F/6M, HC: 2F/6M<br>Measures: TMS administered to left motor cortex, EMG data collected from right APB. CS was set to produce MEP of 0.5-1.0 mV, TS was set to produce MEP of 0.5-1.0 mV ISI: 100 ms.<br>Major outcome: LICI deficit in SCZ patients	There was no significant difference in LICI across medicated SCZ, unmedicated SCZ patients and HC.
Farzan, et al. 2010	Design: Cross-sectional<br>Participants: Total of 42 participants: 14 SCZ, 14 Bipolar, 14 HC<br>*Age (yrs):* SCZ: 37.5± 10.4, Bipolar: 32.6± 13.4, HC: 36.7± 7.6<br>*Sex:* SCZ: 10M/4F, Bipolar: 9M/5F, HC: 9M/5F<br>Measures: TMS administered to left motor cortex and DLPFC, EEG data collected from motor cortex and DLPFC. EMG data collected from the right APB. CS was set to produce MEP of 1.0 mV, TS was set to produce MEP of 1.0 ISI: 100 ms.<br>Major outcome: LICI of gamma oscillations in DLPFC and motor cortex of SCZ patients	SCZ patients demonstrated significant reduction in cortical inhibition of gamma oscillations in DLPFC relative to healthy controls ($p<0.01$) and bipolar patients ($p<0.01$).<br>There was no significant cortical inhibition of gamma oscillation deficit in the motor cortex of SCZ compared to bipolar patients or HC.<br>Significant deficit in bipolar patients was not detected.<br>There was no correlation between LICI deficit and antipsychotic medication dose.
Mehta, et al. 2014	Design: Cross-sectional<br>Participants: Total of 99 participants, 54 SCZ: 33 unmedicated + 21 medicated, 45 HC<br>*Age (yrs):* Unmedicated SCZ: 33.60± 9.74, Medicated SCZ: 29.19± 6.6, HC: 30.68±9.57<br>*Sex:* Unmedicated SCZ: 15F/18M, Medicated SCZ: 12F/9M, HC: 22F/23M<br>Measures: TMS administered to left motor cortex, EMG data collected from right FDI. CS was set to produce MEP of 1.0 mV, TS was set to produce MEP of 1.0 mV. ISI: 100 ms. Social cognition was measured with Social Cognition Rating Tools in Indian Setting (SOCRATIS) and Tool for Recognition of Emotions in Neuropsychiatric Disorders (TRENDS).	There was no significant group difference in LICI parameters at baseline.<br>There was no significant relationship between social cognition measures and LICI in SCZ patients and control group.

	Major outcome: Relationship between LICI and social cognition in SCZ patients	
Mehta, et al. 2014b	Design: Cross-sectional<br>Participants: Total of 99 participants, 54 SCZ: 33 unmedicated + 21 medicated, 45 HC<br>*Age (yrs):* Unmedicated SCZ: 33.60± 9.74, Medicated SCZ: 29.19± 6.6, HC: 30.68±9.57<br>*Sex:* Unmedicated SCZ: 15F/18M, Medicated SCZ: 12F/9M, HC: 22F/23M<br>Measures: TMS administered to left motor cortex, EMG data collected from right FDI. CS was set to produce MEP of 1.0 mV, TS was set to produce MEP of 1.0 ISI: 100 ms. To assess the Mirror neuron activity, experiment was conducted while participants observed goal directed action. Social cognition was measured with Social Cognition Rating Tools in Indian Setting (SOCRATIS) and<br>Tool for Recognition of Emotions in Neuropsychiatric Disorders (TRENDS).<br>Major outcome: Putative MNA activity, measured with LICI, in SCZ patients	LICI was not modulated by action observation in the patient and control group.<br>MNA using LICI did not have any significant correlation with social cognition measures in SCZ and control group.
Radhu, et al. 2015	Design: Cross-sectional<br>Participants Total of 111 participants, 38 SCZ, 27 OCD, 46 HC<br>*Age (yrs):* SCZ: 35.71, OCD: 36.15, HC: 33.63<br>*Sex: SCZ:* 25M/13F, OCD: 11M/16F, HC: 23F/23M<br>Measures: TMS administered to left motor cortex and DLPFC, EEG data collected from motor cortex and DLPFC. EMG data collected from the right APB. CS was set to produce MEP of 1.0 mV, TS was set to produce MEP of 1.0 ISI: 100 ms. Symptom severity of SCZ was measured with Brief Psychiatric Rating Scale (BPRS).<br>Major outcome: LICI in SCZ and OCD patients in DLPFC and motor cortex	SCZ patients had reduced LICI in DLPFC relative to OCD ($p = 0.0465$) and control group ($p = 0.004$).<br>No significant difference between OCD and HC in DLPFC.<br>LICI values in the motor cortex were not significantly different across SCZ, OCD and HC.<br>Increased clinical severity of SCZ was correlated with reduced LICI ($p = 0.0457$).

Basavaraju, et al. 2015	Design: Cross-sectional<br>Participants Total of 50 SCZ patients, 18 with ego-boundary disturbance (EBD), 32 w/o EBD<br>*Age (yrs):* SCZ + EBD: 33.11± 8.20, SCZ w/o EBD: 29.97 ± 8.59<br>*Sex:* SCZ + EBD: 9F/9M, SCZ w/o EBD: 18F/14M<br>Measures: TMS administered to left motor cortex, EMG data collected from right FDI. CS was set to produce MEP of 1.0 mV, TS was set to produce MEP of 1.0 ISI: 100 ms. To assess Mirror neuron activity (MNA), experiment was conducted while participants observed goal directed action.<br>Major outcome: Putative MNA in SCZ patient with and without EBD	No significant difference was detected in MNA measured using LICI between SCZ patients with EBD versus without EBD. (ns)
Lett, et al. 2016	Design: Cross-sectional<br>Participants Total of 195 participants, 80 SCZ, 115 HC. TMS-EEG completed by 23 SCZ, 33 HC<br>*Age (yrs):* SCZ: 34.59± 10.39, HC: 34.58 ±10.93<br>*Sex:* SCZ: 18M/5F, HC: 15M/18F<br>Measures: TMS administered to DLPFC, EEG data collected from DLPFC. CS was set to produce MEP of 1.0 mV, TS was set to produce MEP of 1.0 ISI: 100 ms.<br>Major outcome: Association between glutamic acid decarboxylase 1 (GAD1) variant and cortical inhibition using LICI	GAD1 genotype and diagnosis were not significantly correlated with DLPFC LICI cluster size.<br>In healthy controls GAD1 T allele carriers had greater LICI cluster size. (p= 0.003)<br>In SCZ patients GAD1 T allele carriers had lower cluster size. (p=0.04)
Radhu, et al. 2017	Design: Cross-sectional<br>Participants Total of 129 participants, 19 SCZ/schizoaffective, 30 unaffected first degree relative of SCZ patients, 13 OCD, 18 unaffected first degree relative of OCD patients, 49 HC<br>*Age (yrs):* SCZ: 30.2, SCZ relatives: 53.8, OCD:28.9, OCD relatives: 41.9, HC: 30.2<br>*Sex:* SCZ: 9F/10M SCZ relatives: 17F/13M OCD: 9F/4M OCD relatives: 12F/6M, HC: 25F/24M<br>Measures: TMS administered to left motor cortex and DLPFC, data collected by EEG. EMG data collected from the right APB. CS was set to produce MEP of 1.0 mV, TS was set to produce MEP of 1.0 mV. ISI: 100 ms.<br>Major outcome: LICI in SCZ, OCD patients and their relatives	First degree relatives were found to have intermediate inhibition, LICI, in DLPFC relative to related probands and HC.<br>HC >first-degree relatives of SCZ > SCZ probands.<br>First degree relative vs SCZ LICI difference was found significant in DLPFC (p=0.03).<br>SCZ vs HC LICI difference was found significant in DLPFC (p=0.032).<br>No significant difference was detected between first degree relatives of SCZ vs HC in DLPFC.<br>Frontal gamma inhibition followed the same pattern. (HC >first-degree relatives of SCZ > SCZ probands)<br>No significant difference of LICI in the motor cortex detected between SCZ vs relatives vs HC.<br>There was no significant deficit of LICI in OCD patients compared to relatives of OCD or HC in both motor cortex and DLPFC.<br>There was no significant correlation between antipsychotic medication use and LICI.

Goodman, et al. 2017	Design: Cross- sectional<br>Participants: 12 cannabis dependent SCZ, 11 cannabis free SCZ, 10 cannabis dependent controls, 13 cannabis free controls<br>*Age (yrs):* SCZ+cannabis: 29.4 ± 8.4, SCZ: 38.5 ± 8.9, HC+cannabis: 30.4 ± 7.4, HC: 35.5 ± 10.5<br>*Sex:* SCZ+cannabis: 12M/0F, SCZ cannabis free: 7M/4F, HC+cannabis: 10M/0F, HC: 10M/3F<br>Measures: TMS administered to left motor cortex, EMG data collected from right APB. CS was set to produce MEP of 1.0 mV, TS was set to produce MEP 1.0 mV. ISI: 100, 150, 200 ms.<br>Major outcome: LICI in SCZ patients with and without cannabis use versus controls with and without cannabis use	No significant effect of cannabis use on LICI was observed in both SCZ and control group. (ns)<br>SCZ and control groups did not differ from each other significantly based on LICI.

SCZ: Schizophrenia
HC: Healthy controls
TMS: Transcranial magnetic stimulation
EMG: Electromyography
APB: Abductor Pollicis Brevis
MEP: Mean evoked potential
CS: Conditioning stimulus
TS: Test stimulus
ISI: Interstimulus interval
LICI: Long-interval intracortical inhibition
EEG: Electroencephalogram
DLPFC: Dorsolateral prefrontal cortex
FDI: First dorsal interosseous
MNA: Mirror neuron activity
OCD: Obsessive Compulsive Disorder
EBD: Ego-boundary disturbance

Supplementary Table 6 LICI in patients with Substance use

Reference	Study Description	Findings
Sundaresan, et al. 2007	Design: Cross-sectional<br>Participants: Total of 20 patients, 10 abstinent (3 weeks- 6 months) cocaine dependent, 10 HC<br>*Age (yrs):* Cocaine user: 39.7, HC: 41.2<br>*Sex:* Cocaine user: 5M/5F, HC: 8M/2F<br>Measures: TMS administered to left motor cortex, EMG data collected from right FDI. CS was set to 120% RMT, TS was set to 120% RMT ISI: 50, 100 ms.<br>Major outcome: LICI in abstinent cocaine dependent patients	No significant LICI deficit noted in cocaine dependent patients.
Lang, et al. 2018	Design: Cross-sectional<br>Participants: Total of 38 patients, 19 smoker, 19 non-smokers<br>*Age (yrs):* Smokers: 24 ± 0.44, Non-smokers: 24 ± 0.35<br>*Sex:* Smokers: 13M/6F, Non-smokers: 13M/6F<br>Measures: TMS administered to left motor cortex, EMG data collected right FDI. CS was set to 110% RMT, TS was set to 110% RMT ISI: Mean percentage of inhibition at 50,100, 150 ms.<br>Major outcome: Alteration in LICI in chronic smokers	Smokers did not show any significant deficit of LICI relative to non-smokers.
Fitzgerald, et al. 2009	Design: Cross-sectional<br>Participants: Total of 61 patients, 42 chronic cannabis users: 25 heavy + 17 light user, 19 HC<br>*Age (yrs):* Heavy cannabis users: 28.56 ± 9.45, Light cannabis users: 25.12 ± 6.94, HC: 28.89 ± 9.05<br>*Sex:* Heavy users: 20M/5F, Light users: 11M/6F, HC: 13M/6<br>Measures: TMS administered to motor cortex, EMG data collected contralateral APB. CS was set to 120% RMT, TS was set to 120% RMT ISI: 100 ms.<br>Major outcome: Alteration in LICI in chronic cannabis users	There was no significant difference of LICI in cannabis users relative to nonuser HC.<br>There was no correlation significant between tetrahydrocannabinol levels and LICI.
Gjini, et al. 2012	Design: Cross-sectional<br>Participants: Total of 94 patients, 52 abstinent (3 weeks- 6 months) cocaine dependent subjects, 42 HC<br>*Age (yrs):* Cocaine user: 42, HC: 41 *Sex:* Cocaine user: 48M/4F, HC: 34M/8F<br>Measures: TMS administered to motor cortex, EMG data collected contralateral FDI. CS was set to 120% RMT, TS was set to 120% RMT. ISI: 50, 100 ms.<br>Major outcome: LICI in cocaine dependent patients	There was no significant difference in LICI between cocaine users and HC.

Naim-Feil, et al. 2016	Design: Cross-sectional<br>Participants: Total of 26 participants, 12 alcohol dependent (post detoxification- 2 years) patients, 14 HC<br>*Age (yrs):* Alcohol dependent: 40.1 ± 13.4, HC: 31.2 ± 5.3<br>*Sex:* Alcohol dependent: 8M/4F, 7M/7F<br>Measures: TMS administered to right and left DLPFC. EEG used for data collection. CS was set to produce MEP of 1.0 mV, TS was set to produce MEP 1.0 mV. ISI: 100 ms.<br>Major outcome: LICI alteration in alcohol dependent patients	Alcohol dependent subjects showed reduced LICI in both left and right DLPFC relative to HC. (p= 0.003, 0.006)

HC: Healthy controls
TMS: Transcranial magnetic stimulation
EMG: Electromyography
FDI: First dorsal Interosseous
RMT: Resting motor threshold
CS: Conditioning stimulus
TS: Test stimulus
ISI: Interstimulus interval
LICI: Long-interval intracortical inhibition
APB: Abductor Pollicis Brevis
EEG: Electroencephalogram
DLPFC: Dorsolateral prefrontal cortex
MEP: Mean evoked potential

Supplementary Table 7 LICI in Dementia patients

Reference	Study Description	Findings
Brem, et al. 2013	Design: Cross-sectional<br>Participants: Total of 29 participants, 16 AD patients: 7 on AChEI + 9 on AChEI & Memantine, 13 HC<br>*Age (yrs):* AChEI & Memantine: 71.8 ± 3.73, AChEI: 68.0±7.55, HC: 67.8± 6.05<br>*Sex:* AChEI & Memantine: 6F/3M, AChEI: 5F/2M HC: 7F/6M<br>Measures: TMS administered to left motor cortex, EMG data collected from right FDI. CS was set to 120% RMT, TS was set to 120% RMT ISI: 100 ms. Cognitive function was assessed with ADAS-Cog (higher score indicates greater impairment).<br>Major outcome: LICI in AD patients receiving ACHEI monotherapy or ACHEI combined with Memantine<br>Additional outcome: Cognitive functions and its correlation with cortical inhibition in AD patients	LICI was reduced in both AD patient groups, combination treatment (p=0.025) and AChEI groups (0.015) relative to HC.<br>The LICI difference between two AD patients groups was not significant.<br>Cognitive impairment, measured with ADAS-Cog, was significantly correlated with the reduction in LICI. (p= 0.010).
Benussi, et al. 2016	Design: Cross-sectional<br>Participants: Total of 51 participants, 27 with pathogenic FTD mutation: 13 presymptomatic (baring GRN mutation) +14 symptomatic, 24 HC<br>*Age (yrs):* FTD presymptomatic: 42.0 ± 8.1, 14 symptomatic: 64.8 ± 5.4, HC: 44.7 ± 18.5<br>*Sex:* FTD presympt: 9F/4M, 9F/5M, symptomatic: 9F/5M, HC: 18F/6M<br>Measures: TMS administered to left motor cortex, EMG data collected from right FDI. CS was set at MEP of 130% RMT, TS was set to produce MEP 1.0 mV. ISI: 50, 100, 150 ms. Mean measures of ISIs were used.<br>Major outcome: LICI in symptomatic and presymptomatic genetic FTD subjects	Significant difference in LICI between asymptomatic carriers versus HC was not identified.<br>There was a non-significant trend towards decreased LICI in symptomatic FTD patients.
Benussi, et al. 2017	Design: Cross-sectional<br>Participants: Total of 172 participants, 79 AD, 61 FTD, 32 HC<br>*Age (yrs):* AD: 71.2 ± 8.1, FTD: 65.6 ± 9.2, HC: 61.5 ± 10.3<br>*Sex:* AD: 39F/40M, FTD: 26F/35M, HC: 18F/14M<br>Measures: TMS administered to left motor cortex, EMG data collected from right FDI. CS was set at MEP of 130% RMT, TS was set to produce MEP 1.0 mV. ISI: 50, 100, 150 ms.<br>Major outcome: Utility of LICI as a biomarker distinguishing AD from FTD	Reduced LICI at 150 ms ISI was detected in FTD patients relative to HC. (p= 0.004)<br>There was no significant LICI difference between AD and FTD or AD and HC.

Fried, et al. 2017	<u>Design:</u> Cross-sectional<br><u>Participants:</u> Total of 36 participants, 9 AD, 15 Type 2 DM, 12 HC<br>*Age (yrs):* AD: 67.7 ± 6.9, Type 2 DM: 63.4 ± 7.3, HC: 58.6 ± 9.1<br>*Sex:* AD: 4M/5F, Type 2 DM: 9M/6F, HC: 6M/6F<br><u>Measures:</u> TMS administered to left motor cortex, EMG data collected from right FDI. CS was set at MEP of 120% RMT, CS was set at MEP of 120% rMT. ISI: 100 ms<br><u>Major outcome:</u> Reproducibility of cortical inhibition measures in AD, Type-2 DM and healthy group	LICI demonstrated high reproducibility in AD (α= 0.88) patients and HC (α =0.98).
Benussi, et al. 2019	<u>Design:</u> Cohort<br><u>Participants:</u> Total of 186 participants, 113 monogenic FTD mutation (GRN and C9orf72) carriers, 75 with noncarrier first degree relatives. 74 participants were reevaluated with follow-up sessions.<br>*Age (yrs):* FTD carriers: 54.8 ± 13.2, Noncarriers: 43.6 ±13.7<br>*Sex:* AD: FTD carriers: 66F/47M, Noncarriers: 47F/26M<br><u>Measures:</u> TMS administered to left motor cortex, EMG data collected from right FDI. CS was set at MEP of 130% RMT, TS was set to produce MEP 1.0 mV. ISI: 50, 100, 150 ms. Years from symptom onset was determined by subtracting age of participant from mean familial age at symptom onset.<br><u>Major outcome:</u> LICI change during disease progression in FTD mutation carriers and noncarriers at risk patients.	LICI was significantly reduced in FTD mutation carriers relative to noncarriers, at 20 years before expected symptom onset. ($p < 0.001$)
Assogna, et al. 2020	<u>Design:</u> Cohort<br><u>Participants:</u> Total of 17 patients with probable FTD<br>*Age (yrs):* 62.5 ± 9.43<br>*Sex:*11F/6M<br><u>Measures:</u> TMS administered to left motor cortex, EMG data collected from right FDI. CS was set at MEP of 110% RMT, TS was set to produce MEP 1.0 mV. ISI: 50, 100, 150 ms.<br><u>Major outcome:</u> LICI change following palmitoylethanolamide combined with luteolin (PEA-LUT) administration in patients with FTD.	LICI at 100 ms demonstrated restoration (increased) following PEA-LUT demonstration (p=0.038).
Benussi, et al 2020a	<u>Design:</u> Cross-sectional<br><u>Participants:</u> Total of 186 FTD participants, 130 bvFTD+ 35 avPPA+ 21 svPPA<br>*Age:* bvFTD: 65.8 ± 9.1, avPPA: 65.4± 9.2., svPPA: 68.6 ± 9.0<br>*Sex:* bvFTD: 51F/79M, avPPA: 22F/13M, svPPA: 11F/10M<br><u>Measures:</u> TMS administered to left motor cortex, EMG data collected from right FDI. CS was set at MEP of 130% RMT, TS was set to produce MEP 1.0 mV. ISI: 50, 100, 150 ms. Behavioral symptoms were measured with Frontal Behavioral Inventory (FBI).	LICI was found to be more reduced in GRN mutation carriers (p=0.011).<br>There was a significant association between reduction in LICI and increase in negative symptoms (p<0.001), same applied for positive symptoms (p<0.001). But the significance of the results was lost after multiple regression analysis.

	Major outcome: Correlation of LICI with degree of positive and negative symptoms in FTD patients	
Benussi, et al. 2020b	Design: Cross-sectional<br>Participants: Total of 245 participants, 171 FTD: 122 bvFTD+ 31 avPPA+ 18 svPPA, 74 HC<br>*Age (yrs):* bvFTD: 65.7 ± 9.0, avPPA: 67.7±8.8, svPPA: 63.0±7.8, HC: 64.0 ± 11.5<br>*Sex:* bvFTD: 47F/75M, avPPA: 20F/11M, svPPA: 10F/8M, HC: 45F/29M<br>Measures: TMS administered to left motor cortex, EMG data collected from right FDI. CS was set at MEP of 130% RMT, TS was set to produce MEP 1.0 mV. ISI: 50, 100, 150 ms. Disease severity was measured with FTLD-CDR, MMSE. Behavioral disturbance was measured with BADL, IADL.<br>Major outcome: LICI alteration in different FTD phenotypes.<br>Additional outcome: LICI's relationship with functional decline and symptom stage.	LICI was significantly reduced in all phenotypes of FTD, bvFTD, avPPA, svPPA at all ISI (50,100, 150 ms) relative to HC. ($p < 0.05$)<br>Symptom severity was found to be correlated with LICI deficit. ($p < 0.001$)<br>Disease duration was correlated with LICI reduction. ($p < 0.001$)<br>Reduction in LICI was correlated with functional decline. ($p < 0.001$)
Benussi, et al. 2020c	Design: Cross-sectional<br>Participants: Total of 66 with FTD<br>*Age (yrs):* 63.6 ± 8.2<br>*Sex:* 29F/37M<br>Measures: TMS administered to left motor cortex, EMG data collected from right FDI. CS was set at MEP of 130% RMT, TS was set to produce MEP 1.0 mV. ISI: 50, 100, 150 ms. Meta-state indexes measured with MRI were used to evaluate brain network connectivity and fluidity.<br>Major outcome: LICI's association with whole brain network fluidity.	There was no significant association between LICI and meta-state indexes.

AD: Alzheimer's disease
AChEI: Acetylcholinesterase inhibitors
HC: Healthy controls
TMS: Transcranial magnetic stimulation
EMG: Electromyography
FDI: First dorsal interosseous
CS: Conditioning stimulus
RMT: Resting motor threshold
TS: Test stimulus
ISI: Interstimulus interval
ADAS-Cog: Alzheimer disease assessment scale-cognitive subscale
LICI: Long-interval intracortical inhibition
FTD: Frontotemporal dementia
GRN: Granulin
DM: Diabetes Mellitus
bvFTD: Behavioral variant of FTD
avPPA: Agrammatic variant of primary progressive aphasia
svPPA: Semantic variant of primary progressive aphasia
FTLD-CDR: Frontotemporal Lobar Degeneration-modified Clinical Rating Scale
MMSE: Mini Mental State Examination
BADL: Basic Activities of Daily Living
IADL: Instrumental Activities of Daily Living

Supplementary Table 8 LICI in Epilepsy patients

Reference	Study Description	Findings
Brodtmann, et al, 1999	Design: Cross-sectional<br>Participants: Total of 23 participants: 7 idiopathic generalized epilepsy patients, 16 HC.<br>*Age (yrs):* IGE patients: 25, HC: 29<br>*Sex:* IGE patients: 5M/2F, HC: 7M/9F<br>Measures: TMS administered to dominant motor cortex, EMG data collected from APB. Both CS and TS were set to 115% RMT. ISI: 50, 75, 100, 125, 150, 175, 200, 225, 250, 300, 350 and 400 ms.<br>Major outcome: LICI alteration in IGE patients	LICI was reduced in epilepsy patients relative to HC at interstimulus intervals of 200, 225, 250, 300 ms and there was significant facilitation of motor evoked potential instead of inhibition at the same ISI ($p<0.05$, $p<0.005$, $p<0.0005$, $p<0.05$).
Valzania, et al. 1999	Design: Cross-sectional<br>Participants: Total of 20 participants: 12 progessive myoclonic epilepsy patients, 8 HC.<br>*Age (yrs):* PME patients: 24.2, HC: 30.8<br>*Sex:* PME patients: 4F/8M, HC: 6M/2F<br>Measures: TMS administered to motor cortex, EMG data collected from ADM. Both CS and TS were set to 110% RMT. ISI: 50,100,150 and 200 ms.<br>Major outcome: LICI alteration in PME patients	LICI was reduced in PME subjects relative to HC at interstimulus intervals of 100-150 ms and 50 ms ($p<0.001$).<br>At interstimulus of 50 ms PME patients demonstrated facilitation instead of inhibition.
Manganotti, et al. 2000	Design: Cross-sectional<br>Participants: Total of 27 participants: 15 juvenile myoclonic epilepsy patients, 12 HC<br>*Age (mean not given, Range, yrs):* JME patients: 20-38, HC: 24-39<br>*Sex:* JME patients: 11F/4M, HC: 7F/5M<br>Measures: TMS administered to left and right motor cortex, EMG data collected from thenar eminence muscles. Both CS and TS were set to 120% RMT. ISI: 30, 50, 70, 100, 125, 150, 200, 250, 300, and 400 ms.<br>Major outcome: LICI alteration in JME patients	There was no significant difference between JME patients and controls in means of LICI.

Molnar, et al. 2006	Design: Clinical trial<br>Participants: Total of 14 participants: 5 Epilepsy patients, 9 HC<br>*Age (yrs):* Patients (mean not given): 23, 33, 40, 43, 48, HC: 56 ± 15<br>*Sex:* Patients: 3M/2F, HC: 7M/2F<br>Measures: TMS administered to left motor cortex, EMG data collected from right FDI. CS adjusted to produce 1 mV MEP; TS adjusted to produce 1 mV MEP. ISI: 50-200 ms. The muscle was tested both in rest and active conditions. DBS was tested in three conditions being OFF, cycling and continuous stimulation<br>Major outcome: Effect of DBS on LICI in epilepsy patients	DBS did not have a significant effect on LICI.<br>LICI in all three stimulus conditions (off, cycling and continuous) was impaired relative to HC at ISI of 50 ms in the resting muscle (p=0.0003, 0.0015. 0.0001)
Badawy, et al. 2007	Design: Cross-sectional<br>Participants: Total of 91 participants: 35 IGE patients, 27 focal epilepsy patients, 29 HC<br>*Age (yrs):* IGE patients: 25, focal epilepsy patients: 32, HC: 33<br>*Sex:* IGE patients: 18F/17M, focal epilepsy patients: 16F/11M, HC: 12F/ 17M<br>Measures: TMS administered to both hemispheres in 32 of 35 IGE patients and all focal epilepsy patients. 12 of the HC received stimulation to both hemispheres and 17 only to the dominant hemisphere. EMG data collected from APB. Both CS and TS were set to 120% RMT. ISI: 200-400 ms.<br>Major outcome: LICI alteration in antiepileptic drug naive epilepsy patients	IGE group demonstrated facilitation of MEP at long interstimulus intervals therefore LICI was reduced relative to healthy controls (p<0.01 at 250 ISI).<br>There was no significant difference between hemispheres.<br>Same findings applied for ipsilateral LICI measures of focal epilepsy patients (p<0.01 at 250 ISI).<br>There was no significant LICI difference between controls and contralateral hemisphere measure of focal epilepsy patients.
Badawy, et al. 2010	Design: Clinical trial<br>Participants: Total of 138 participants: 59 IGE patients, 47 focal epilepsy patients, 32 HC<br>*Age (yrs):* IGE patients: 29, focal epilepsy patients: 37, HC: 31<br>*Sex:* IGE patients: 32F/27M, focal epilepsy patients: 29F/18M, HC: 20F/ 12M<br>Measures: TMS administered to the dominant hemisphere in IGE patients and both hemispheres in focal epilepsy patients and HC. EMG data collected from APB. Both CS and TS were set to 120% RMT. ISI: 50-300 ms. Investigations were performed right before and 4-16 weeks after treatment.<br>Major outcome: Effect of anti-epileptic drug treatment on LICI in epilepsy patients.	Dominant hemisphere LICI impairment (reduced) at baseline in seizure free IGE patients, was restored (increased) following AED treatment at interstimulus intervals of 50, 150, 250 and 300 ms (p<0.01).<br>LICI did not change significantly in IGE patients with ongoing seizures after AED treatment.<br>Reduced LICI in the ipsilateral hemisphere of focal epilepsy patients was restored following AED treatment in seizure free group at interstimulus intervals of 250-300 ms (p<0.01).<br>LICI did not change significantly in focal epilepsy patients with ongoing seizures after AED treatment.

Badawy and Jackson, 2012	Design: Cross-sectional<br>Participants: Total of 95 participants: 26 migraine patients, 22 focal epilepsy patients, 28 IGE patients, 19 HC<br>*Age (yrs):* Migraine patients: 19, focal epilepsy patients: 21, IGE patients: 20, HC: 20<br>*Sex:* Migraine patients: 8M/18F, focal epilepsy patients: 7M/15M, IGE patients: 10M/ 18F, HC: 13F/6M<br>Measures: TMS administered both hemispheres in all subjects. EMG data collected from APB. Both CS and TS were set to 120% RMT. ISI: 50-400 ms.<br>Major outcome: LICI alteration in migraine and epilepsy patients	LICI was reduced in migraine patients relative to HC at ISIs of 250 ms ($p<0.05$).<br>In IGE patients and the ipsilateral hemisphere of focal epilepsy patients LICI was reduced relative to HC at interstimulus intervals of 250 and 300 ms.<br>LICI deficit was greater in focal epilepsy ($p<0.05$) and IGE patients ($p<0.01$) relative to migraine patients at ISI of 250 ms.
Badawy, et al. 2012	Design: Cross-sectional<br>Participants: Total of 41 participants: 11 focal epilepsy patients, 13 IGE patients, 17 HC<br>*Age (yrs):* Focal epilepsy patients: 31, IGE patients: 22, HC: 30<br>*Sex:* Focal epilepsy patients: 6F/5M, IGE patients: 8F/5M, HC: 11F/6M<br>Measures: TMS administered both hemispheres in all subjects. EMG data collected from APB. Both CS and TS were set to 120% RMT. ISI: 50-400 ms. Subjects were investigated in two separate sessions 4-20 weeks apart.<br>Major outcome: Reproducibility of LICI in drug naive epilepsy patients	LICI was reduced in IGE patients relative to HC at ISIs of 50, 150, 250, 300 ms and the ipsilateral side of focal epilepsy patients at ISIs of 250, 300 ms (p values not given).<br>There was no significant inter session variability in all groups (rho value ranging from 0.93-0.95).
Badawy, et al. 2013a	Design: Clinical trial<br>Participants: Total of 77 participants: 30 Refractory epilepsy patients: 14 IGE + 16 focal epilepsy, 35 seizure free on monotherapy patients: 18 IGE + 17 focal epilepsy, 12 seizure free on dual therapy patients: 5 IGE + 7 focal epilepsy.<br>*Age (yrs):* Refractory epilepsy patients: IGE: 23, focal epilepsy: 31, Seizure free on monotherapy patients: IGE: 24, Focal epilepsy: 30, Seizure free on dual therapy patients: IGE: 24, Focal epilepsy: 30<br>*Sex:* Refractory epilepsy patients: IGE: 8F/6M, focal epilepsy: 7F/9M, Seizure free on monotherapy patients: IGE: 12F/6M, Focal epilepsy: 10F/7M Seizure free on dual therapy patients: IGE: 2F/3M, Focal epilepsy: 3F/4M<br>Measures: TMS administered both hemispheres in all subjects. EMG data collected from APB. Both CS and TS were set to 120% RMT. ISI: 100-300 ms. Subjects were investigated in 4 phases: in 1-2 weeks, 2-6 months, 12-18 months, 30-36 months.<br>Major outcome: Effect of AEDs on LICI in epilepsy patients over time	At 30-36 months the refractory group of both IGE and focal epilepsy patients had a progressively worsening LICI ($p<0.01$).<br>Seizure free IGE (both hemispheres) and focal epilepsy patients (ipsilateral hemisphere) on monotherapy and dual therapy showed significant enhancement in LICI at multiple interstimulus intervals ($p<0.05$).

Badawy, et al. 2013b	<u>Design:</u> Cross-sectional<br><u>Participants:</u> Total of 157 participants: JME patients: 10 drug naive + 16 refractory + 20 seizure free, JAE patients: 8 drug naive + 15 refractory + 18 seizure free, GE-TCS patients: 12 drug naive + 18 refractory + 20 seizure free, 20 HC<br>*Age (yrs):* JME patients: Drug naive: 20, Refractory: 25, Seizure-free: 22 JAE patients: Drug naive: 18, Refractory: 24, Seizure-free: 24 GE-TCS patients: Drug naive: 20, Refractory: 25, Seizure-free: 26, HC: 27.<br>*Sex:* JME patients: Drug naive: 6F/4M, Refractory: 9F/7M, Seizure-free: 11F/9M. JAE patients: Drug naive: 5F/3M, Refractory: 7F/8M, Seizure-free: 7F/11M. GE-TCS patients: Drug naive: 7F/5M, Refractory: 9F/9M, Seizure-free: 9F/11M. HC: 11F/9M.<br><u>Measures:</u> TMS administered both hemispheres in all subjects. EMG data collected from APB. Both CS and TS were set to 120% RMT. ISI: 100-300 ms.<br><u>Major outcome:</u> LICI alteration in different generalized epilepsy syndromes	All drug naive epilepsy groups, JME, JAE and GE-TCS subjects, demonstrated reduced LICI relative to HC at interstimulus intervals of 150, 250 and 300 ms ($p<0.01$).<br>Drug naive JME patients had a greater LICI deficit relative to JAE and GE-TCS subjects ($p<0.01$).<br>In refractory seizure groups JME subjects had a greater LICI deficit at interstimulus intervals of 100 and 200 ms relative to JAE and GE-TCS patients. ($p< 0.05$).<br>Same applied for seizure free groups results showing worse LICI in JME subjects relative to JAE and GE-TCS subjects at ISIs ot 150, 250 and 300 ms ($p< 0.05$).
Badawy, et al. 2013c	<u>Design:</u> Cross-sectional<br><u>Participants:</u> Total of 105 participants, 85 Temporal lobe epilepsy patients: 10 drug naive new onset, 13 early medically refractory, 14 late medically refractory, 12 early seizure free on AEDs, 12 late seizure free on AEDs, 12 postoperative refractory seizure, 12 postoperative seizure free, 20 HC<br>*Age (yrs):* Drug naive new onset: 24, Early medically refractory: 27, Late medically refractory: 25, Early seizure free on AEDs: 26, Late seizure free on AEDs: 26, Postoperative refractory seizure: 31, Postoperative seizure free: 29, HC: 27<br>*Sex:* Drug naive new onset: 5F/5M, Early medically refractory: 7F/6M, Late medically refractory: 8F/6M, Early seizure free on AEDs: 5F/7M, Late seizure free on AEDs: 7F/5M, Postoperative refractory seizure: 6F/6M, Postoperative seizure free: 9F/3M HC: 11F/9M<br><u>Measures:</u> TMS administered both hemispheres in all subjects. EMG data collected from APB. Both CS and TS were set to 120% RMT. ISI: 100-300 ms.<br><u>Major outcome:</u> LICI alteration in temporal lobe epilepsy patients with different levels of seizure control	Early onset drug naive TLE patients had reduced LICI in the ipsilateral hemisphere relative to HC whereas contralateral hemisphere did not significantly differ from HC. ($p<0.01$) .<br>Refractory seizure patients (both medical and postoperative) had reduced LICI in both hemispheres relative to HC ($p<0.01$).<br>LICI was more reduced in refractory seizure patients (both medical and postoperative) compared to drug naive new onset patients and seizure free control group ($p<0.05$).

Badawy, et al. 2013d	Design: Cross-sectional<br>Participants: Total of 32 participants, 22 drug naive epilepsy patients: 11 IGE + 11 Focal, 10 HC<br>*Age (yrs):* IGE patients: 19, focal epilepsy: 25, HC: 20<br>*Sex:* IGE patients: 6F/5M, focal epilepsy: 5F/6M, HC: 5F/5M<br>Measures: TMS administered both hemispheres in all subjects. EMG data collected from APB. Both CS and TS were set to 120% RMT. ISI: 100-400 ms. Subjects were studied in 2 sessions, 12 h before fasting and 2 hours after food ingestion. Serum glucose levels were measured right before each TMS session.<br>Major outcome: LICI alteration in epilepsy patients in relation to blood glucose levels.	LICI was reduced in fasting relative to postprandial state in healthy controls in both hemispheres at interstimulus intervals of 250 and 300 ms ($p<0.05$)<br>LICI was more reduced in fasting state relative to postprandial state in both hemispheres of IGE patients at interstimulus intervals of 300-350 ms ($p<0.05$).<br>Focal epilepsy patients demonstrated lower LICI in fasting state relative to postprandial state in both hemispheres, but alteration was more prominent in the ipsilateral hemisphere ($p<0.05$).
Badawy, et al. 2014	Design: Cross-sectional<br>Participants: Total of 79 participants, 21 isolated unprovoked seizure patients, 20 IGE patients, 18 Focal epilepsy patients, 20 HC<br>*Age (mean not given, range):* Patients: Isolated seizure: 16-34, IGE: 14-26, focal epilepsy: 14-32, HC: 16-40.<br>*Sex:* Patients: Isolated seizure: 11F/10M, IGE: 11F/9M, Focal epilepsy: 10F/8M, HC: 11F/9M<br>Measures: TMS administered to both hemispheres in all subjects. EMG data collected from APB. Both CS and TS were set to 120% RMT. ISI: 100-300 ms.<br>Major outcome: LICI alteration in isolated seizure and epilepsy patients	LICI was reduced in isolated seizure group relative to HC at interstimulus intervals of 250 and 300 ms. ($p<0.05$).<br>LICI was more reduced in generalized epilepsy ($p<0.01$) and focal epilepsy patients ($p<0.05$) relative to isolated seizure subjects at interstimulus intervals of 250 and 300 ms.
Badawy et al. 2015	Design: Cross-sectional<br>Participants: Total of 105 participants, 46 TLE patients: 10 Drug naive new onset + 20 Refractory seizure + 16 Seizure free, 39 Extra-TLE patients: 8 Drug naive new onset + 18 Refractory seizure + 13 Seizure free, 20 HC<br>*Age (yrs):* TLE patients: Drug naive: 24, Refractory seizure: 27, Seizure free: 26. Extra-TLE: Drug naive: 25, Refractory seizure: 27, Seizure free: 29. HC: 27<br>*Sex:* TLE patients: Drug naive: 5F/5M, Refractory seizure: 8F/12M, Seizure free: 8F/8M. Extra-TLE: 5F/3M, Refractory seizure: 9F/9M, Seizure free: 7F/6M. HC: 11F/9M<br>Measures: TMS administered to both hemispheres in all subjects. EMG data collected from APB. Both CS and TS were set to 120% RMT. ISI: 100-300 ms.<br>Major outcome: LICI alteration in focal epilepsy patients with different epileptogenic regions.	Both drug naive TLE and Extra-TLE patient groups demonstrated lower LICI relative to HC in the ipsilateral hemisphere at interstimulus intervals of 250 and 300 ms ($p<0.01$).<br>Interhemispheric difference was significant for both drug naive groups at interstimulus intervals of 250 and 300 ms ($p<0.01$).<br>Drug naive, refractory and seizure free TLE patients and extra-TLE patients did not have significantly different LICI.<br>In refractory TLE and Extra-TLE patients both hemispheres demonstrated lower LICI relative to healthy controls ($p<0.01$).<br>In seizure free TLE and Extra-TLE patients only the affected hemisphere demonstrated significantly lower LICI relative to HC ($p<0.05$).

		LICI in both hemispheres was reduced in refractory TLE and Extra-TLE patients relative to seizure free and drug naive ones ($p<0.05$).
Silbert, et al. 2015	Design: Cross-sectional<br>Participants: Total of 22 participants: 10 IGE patients, 12 HC<br>*Age (yrs):* Patients: 23, HC: 23<br>*Sex:* Patients: 4M/6F, HC: 9M/3F<br>Measures: TMS administered to left motor cortex, EMG data collected from right FDI. CS adjusted to produce 1 mV MEP; TS adjusted to produce 1 mV MEP. ISI: 100-350 ms. 4 patients were evaluated in a second session as 3 of them started AEDs and 1 ceased AED during study.<br>Major outcome: LICI alteration in IGE patients	LICI was not significantly different between unmedicated IGE patients and HC.<br>Patients on AED treatment demonstrated enhanced LICI relative to HC ($p=0.003$) and unmedicated IGE patients at ISI of 200-250 ms ($p<0.001$).
Pawley et al. 2017	Design: Cross-sectional<br>Participants: Total of 96 participants: 28 patients with moderately controlled epilepsy, 40 with poorly controlled epilepsy, 28 HC.<br>*Age (yrs):* Moderately controlled epilepsy patients: 36.25 ± 13.14, Poorly controlled: 40.90 ± 14.64, HC: 33.46 ± 8.25<br>*Sex:* Moderately controlled epilepsy patients: 16F/12M, Poorly controlled: 21F/19M, HC: 15F/13M<br>Measures: TMS administered to both motor cortices, EMG data collected from right FDI. Both CS and TS were set to 120% RMT. ISI: 50, 150, 200, 250 ms. 4 patients were evaluated in a second session as 3 of them started AEDs and 1 ceased AED during study.<br>Major outcome: LICI alteration in long standing uncontrolled epilepsy patients.	There was no significant LICI difference across moderately controlled generalized epilepsy patients, poorly controlled generalized epilepsy patients and HC.<br>In focal epilepsy patients LICI at the interstimulus interval of 200 ms was enhanced in poorly and moderately controlled patients relative to HC ($p=0.040$).

Bauer, et al. 2018	Design: Cross-sectional<br>Participants: Total of 204 participants: 40 Refractory IGE patients, 69 refractory focal epilepsy patients, 95 HC. 21 HC were included in centre 1, 37 HC in centre 2, 37 controls 23 IGE patients and 28 focal epilepsy patients in centre 3, 17 IGE and 41 focal epilepsy patients in centre 4.<br>*Age (yrs):* Centre 1 HC: 28.6, Centre 2 HC: 38.1, Centre 3 HC: 30.2, IGE patients: 30.1, focal epilepsy patients: 39.4, Centre 4 IGE: 34.4, focal epilepsy patients: 39.7<br>*Sex:* IGE patients: 18M/22F, focal epilepsy patients: 30M/39F, *HC*: 36M/59F<br>Measures: Experiments were conducted in 4 different centers. TMS administered to motor hotspot in centre 1, 3 and 4, and to vertex in centre 2. EMG data collected from ADM in centre 1, APB in centre 2 and 4, FDI in centre 3. CS and TS used in centre 1 and 3 was 120% of RMT, and 110% of RMT in centre 2 and 4. ISI: 50, 150, 200, 250 ms.<br>Major outcome: LICI alteration in long standing uncontrolled epilepsy patients.	Results including findings of all of the studies showed no significant LICI difference across HC, IGE patients and focal epilepsy patients.
Bolden, et al. 2018	Design: Cross-sectional<br>Participants: Total of 54 participants: 30 IGE patients: 16 controlled + 14 treatment resistant, 24 HC.<br>*Age (yrs):* Controlled IGEs: 28.63 ± 7.99, Treatment resistant: 26.07 ± 10.48, HC: 30.04 ± 7.28<br>*Sex:* Controlled IGEs: 9F/7M, Treatment resistant: 12F/2M, HC not given<br>Measures: TMS administered to the right motor cortex, EEG used for data collection. CS was set to produce 120% RMT, TS was set to produce 120% RMT. ISI: 50-200 ms. Attention was assessed with Digit Span Forwards, Digit Span Backwards, Trails A and Flanker tasks. Executive functioning was measured with Trails B, Stroop Color and Word, and the Wisconsin Card Sorting tasks.<br>Major outcome: Association of LICI and cognition in IGE patients	There was no significant LICI difference between health controls, controlled IGE patients and treatment resistant IGE patients.<br>Patients with reduced LICI performed worse in attention tasks Digit Forwards ($p=0.049$) and Digit Backwards ($p=0.001$).<br>There was no significant association between executive functioning measures and LICI.
Bolden et al. 2019	Design: Cross-sectional<br>Participants: Total of 52 participants: 30 IGE patients: 16 controlled + 14 treatment resistant, 22 HC.<br>*Age (yrs):* Controlled IGEs: 28.63 ± 7.99, Treatment resistant: 26.07 ± 10.48, HC: not given<br>*Sex:* Controlled IGEs: 9F/7M, Treatment resistant: 12F/2M, HC not given<br>Measures: TMS administered to the right motor cortex, EEG used for data collection. CS adjusted to produce 120% RMT, TS adjusted to produce 120% RMT. ISI: 50-200 ms. The Profile of Mood States (POMS) questionnaire was used to measure mood disturbances.<br>Major outcome: Association of LICI with mood state in IGE patients	Participants with excitatory response on LICI endorsed greater mood disturbance compared to participants with inhibitory response ($p=0.04$).

Huang, et al. 2019	Design: Cross-sectional<br>Participants: Total of 156 participants: 41 Poorly controlled TLE, 71 Well controlled TLE, 44 HC.<br>*Age (yrs):* Poorly Controlled TLE: 38.7 ± 10.3, Well controlled: 28.5 ± 11.7, HC: 37.6 ± 8.70<br>*Sex:* Poorly Controlled TLE: 19M/22F, Well controlled: 45M/26F, HC: 21M/23F<br>Measures: TMS administered to the affected cortex in patients and randomly selected side in controls, EMG data collected from right APB. CS was set to produce 120% RMT, TS was set to produce 120% RMT. ISI: 50, 100, 150, 200, 250, 300 ms.<br>Major outcome: Feasibility of LICI as a biomarker of seizure controllability in temporal lobe epilepsy patients	LICI was enhanced in poorly controlled TLE and well controlled TLE groups relative to HC at interstimulus intervals of 50 ms (p=0.026), 100 ms (p=0.002), 200 ms (0.001).<br>Lamotrigine caused an increase in LICI (p=0.0017)

HC: Healthy controls
TMS: Transcranial magnetic stimulation
EMG: Electromyography
APB: Abductor Pollicis Brevis
RMT: Resting motor threshold
CS: Conditioning stimulus
TS: Test stimulus
ISI: Interstimulus interval
LICI: Long-interval intracortical inhibition
IGE: Idiopathic generalized epilepsy
ADM: Abductor digiti minimi
PME: Progressive myoclonic epilepsy
JME: Juvenile myoclonic epilepsy
FDI: First dorsal interosseous
MEP: Mean evoked potential
DBS: Deep brain stimulation
AED: Antiepileptic drug
JAE: juvenile absence epilepsy
GE-TCS: Generalized epilepsy with tonic-clonic seizures
TLE: Temporal lobe epilepsy
EEG: Electroencephalogram

Supplementary Table 9 LICI in patients with Movement disorders

Reference	Study Description	Findings
Berardelli, et al. 1996	Design: Clinical trial<br>Participants: Total of 31 participants: 20 PD patients, 11 HC<br>*Age (yrs):* PD: 64.5 ± 2.5, HC: 60.5 ± 3.7<br>*Sex:* PD: 17M/3F, HC: not listed<br>Measures: TMS administered to left motor cortex, EMG data collected from contralateral FDI. CS was set to 150% RMT, TS was set to 125% RMT. ISI: 100, 150, 200, 250 ms. Measures were taken during slight voluntary contraction.<br>Major outcome: LICI alteration in PD patients.<br>Additional outcome: LICI change with L-dopa treatment in PD patients	LICI was enhanced in PD patients relative to HC at interstimulus intervals of 150 and 200 ms ($p< 0.05$)<br>LICI decreased significantly following L-dopa administration at 150 ms and 200 ms ($p= 0.01$).
Tagenhoff, et al. 1996	Design: Cross-sectional<br>Participants: Total of 34 participants: 13 HD patients: 10 classical type HD + 2 Westphal variant of HD + 1 juvenile rigid form HD, 21 HC<br>*Age (yrs):* Classical HD: 47.4 ± 9.4, Juvenile rigid HD: 27 yrs, Westphal HD: 51 and 38 yrs, HC: 40.6 ± 12.8<br>*Sex:* Classical HD: 9M/1F, Juvenile rigid HD: 1M, Westphal HD: 1F/1M, HC: 8F/13M<br>Measures: TMS administered to both motor cortex, EMG data collected from FDI. CS was set to 150% RMT, TS was set to 100% RMT. ISI: 1-999 ms.<br>Major outcome: LICI alteration in HD patients.	LICI was prolonged in classical hypotonic-hyperkinetic HD patients ($p<0.05$).<br>Patients suffering from Westphal variant had shortened LICI relative to classical type HD patients ($p<0.05$).
Chen, et al. 1997	Design: Cross-sectional<br>Participants: Total of 26 participants: 8 Writer's cramp patients: left (symptomatic) hemisphere studied in all of patients and right hemisphere tested in 6 patients, 18 HC: left hemisphere studied in 13 HC and right hemisphere tested in 7 HC.<br>*Age (yrs):* L side patients: 43 ± 10.6, R side patients: 40.5 ± 7.3, L side HC: 47.5 ± 13.7, R side HC: 41.2 ± 12.4<br>*Sex:* L side patients: 4M/4F, R side patients: 3M/3F, L side HC: 9M/4F, R side HC: 3M/4F<br>Measures: TMS administered to motor cortex, EMG data collected from extensor carpi radialis. CS was set to 110% RMT, TS was set to 110% RMT. ISI: 20-200ms. Data collected both at rest and active muscle.<br>Major outcome: LICI alteration in writer's cramp patients at rest and during muscle activation.	LICI measured from the symptomatic (left) hemisphere was reduced in the patient group relative to HC during voluntary muscle activation most prominently at 50-80 ms interstimulus interval ($p= 0.02$).<br>There was no significant difference between groups at rest in both hemispheres, and in the right hemisphere during muscle activation.

Valzania et al, 1997	Design: Cross-sectional<br>Participants: Total of 25 participants: 13 PD patients, 12 HC<br>*Age (yrs):* PD: 62.5, HC: 58.1<br>*Sex:* PD: 9M/4F, HC: 7M/5F<br>Measures: TMS administered to left motor cortex, EMG data collected from contralateral ADM. CS was set to produce 120%-140%, 160% and 180% RMT, TS was set to produce 120%-140%, 160% and 180% RMT. ISI: 40, 50, 75, 100, 150, 200 and 300 ms.<br>Major outcome: LICI alteration in PD patients.	Greater LICI in PD patients relative to HC was observed at ISIs of 40, 50, 75 ms measured with TS of 120% RMT ($p<0.01$).
Romeo, et al. 1998	Design: Cross sectional<br>Participants: Total of 18 participants: 10 Essential tremor patients, 8 HC<br>*Age (mean not given, Range):* ET: 47-75 yrs, HC: 42-71 yrs old<br>*Sex:* ET: 8M/2F, HC: not listed<br>Measures: TMS administered to left motor cortex, EMG data collected from right FDI. CS was set to produce 150% of RMT, TS was set to produce 125% of RMT. ISI: 100, 150, 200 ms. LICI was tested in both resting and voluntary contraction states.<br>Major outcome: LICI alteration in patients with essential tremor.	There was no significant LICI alteration in ET patients relative to HC.
Rona, et al. 1998	Design: Cross-sectional<br>Participants: Total of 21 participants: 10 Right arm dystonia patients, 11 HC<br>*Age (yrs):* Dystonia patients: 36 ± 9.7, HC: 40 ± 11.4<br>*Sex:* Dystonia patients: 6F/4M, HC: 3F/8M<br>Measures: TMS administered to the left motor cortex, EMG data collected from contralateral FDI. CS adjusted to produce 150% of RMT, TS adjusted to produce 125% of RMT. ISI: 100, 150, 200 and 250 ms.<br>Major outcome: LICI alteration in dystonia patients	Dystonia patients demonstrated enhanced LICI relative to HC at the ISI of 150 ms ($p=0.017$)
Priori, et al. 2000	Design: Cross-sectional<br>Participants: Total of 44 participants: 16 HD patients, 28 HC<br>*Age (Mean ages not listed, range):* HD: 32-72 yrs, HC: 24-73 yrs<br>*Sex:* HD: 9M/7F, HC: not listed<br>Measures: TMS administered to left motor cortex, EMG data collected from FDI. CS was set to 150% RMT, TS was set to 125%. ISI: 100, 150, 200 and 250 ms. Experiments were conducted during voluntary muscle contraction.<br>Major outcome: Cortical excitability alteration, measured with LICI in HD patients.	There was no significant LICI difference between groups.

Chen, et al. 2001	Design: Clinical trial<br>Participants: Total of 14 participants: 7 PD patients, 7 HC<br>*Age (yrs):* PD: 64.7 ± 3.0, HC: 63.3 ± 3.9<br>*Sex:* PD: 4M/3F, HC: 6M/ 1F<br>Measures: TMS administered to motor cortex ipsilateral to the stimulator site, EMG data collected from right FDI. CS was set to produce MEP of 1.0 mV, TS was set to produce MEP 1.0 mV. ISI: 50,100,150, 200 ms. LICI was tested in OFF, half the amplitude and ON stimulator conditions and at both resting and voluntary contraction.<br>Major outcome: LICI alteration with Gpi stimulation in PD patients.	LICI of the patient group did not significantly differ from the control group whether on or off stimulation both at rest and voluntary contraction.<br>LICI did not change significantly in three different stimulator condition states at both rest and voluntary contraction.
Pierantozzi, et al. 2001	Design: Clinical trial<br>Participants: Total of 58 participants: 29 PD patients, 29 HC<br>*Age (yrs):* PD: 62.1 ± 10.2, HC: 60.1 ± 9.1<br>*Sex:* PD: 16F/13M, HC: 14F/ 15M<br>Measures: TMS administered to motor cortex contralateral to the more affected side of patients, and left cortex in healthy subjects. EMG data collected from APB. CS was set to 120% RMT, TS was set to 120% RMT. ISI: 20-40- 60-80-100-150-200 ms.<br>Major outcome: LICI alteration in PD patients.<br>Additional outcome: Effect of Apomorphine on LICI in PD patients	LICI was reduced in untreated PD patients relative HC ($p < 0.001$) and difference between groups was most significant at 80-100 ms. Apomorphine administration restored LICI in PD patients ($p < 0.0001$).
Cunic, et al. 2002	Design: Clinical trial<br>Participants: Total of 20 participants: 12 PD patients recruited 9 of them used in analysis, 8 HC<br>*Age (yrs):* PD: 59.2 ± 12.1, HC: 59.6 ± 13.7<br>*Sex:* PD: 7M/2F, HC: 6M/ 2F<br>Measures: TMS administered to motor cortex ipsilateral to the stimulator site, EMG data collected from contralateral FDI. CS was set to produce MEP of 1.0 mV, TS was set to produce MEP 1.0 mV. ISI: 50-200 ms. LICI was tested in OFF, half the amplitude and ON STN stimulator conditions.<br>Major outcome: Effect of STN stimulation on LICI in PD patients	LICI in the PD patient was not significantly different compared to HC.<br>Stimulator condition did not affect LICI significantly.

Bares, et al. 2003	Design: Cross-sectional<br>Participants: Total of 22 participants: 12 PD patients, 10 HC<br>*Age (yrs):* PD: 60.4 ± 10.2, HC: 58.5 ± 8.9<br>*Sex:* PD: 7F/5M, HC: 6F/ 4M<br>Measures: TMS administered to the motor cortex, EMG data collected from FDI. Suprathreshold CS (intensity was not indicated), TS was set to 125% RMT. ISI: 100, 150, 200, 250 ms.<br>Major outcome: LICI alteration in L-DOPA and dopamine agonist naive PD patients	There was no significant LICI difference between PD patients and HC.
Sailer, et al. 2003	Design: Clinical trial<br>Participants: Total of 20 participants: 10 PD patients, 10 HC<br>*Age (yrs):* PD: 58.2 ± 9.8, HC: 59.5 ± 10.7<br>*Sex:* PD: 7M/3F, HC: 6M/ 4F<br>Measures: TMS administered left motor cortex ipsilateral, EMG data collected from right FDI. CS produces MEP of 1.0 mV, TS produces MEP > 1.0 mV. ISI: 100 ms. PD patients were tested both on and off medication. Long latency afferent inhibition was elicited by median nerve stimulation 200 ms before test stimulus.<br>Major outcome: Effect of medication and long latency afferent inhibition on LICI in PD patients	LICI did not differ significantly across control, PD off medication and PD on medication groups.<br>LAI exhibited inhibitory effect on LICI more in the control group relative to PD on medication (p=0.039 for more affected side, p= 0.034 for less affected side) and PD off medication (p=0.034 for more affected side, p= 0.005 for less affected side) groups.
Espay, et al. 2006	Design: Cross-sectional<br>Participants: Total of 30 participants: 10 psychogenic dystonia and 8 organic dystonia patients, 12 HC<br>*Age (yrs):* Psychogenic dystonia: 48.1 ± 12.6, Organic dystonia patients: 51.9 ± 11.6, HC: 43.6 ± 13.0<br>*Sex:* Psychogenic dystonia: 9F/1M, Organic dystonia: 5F/3M, HC: not listed<br>Measures: TMS administered to affected motor cortex of patients and dominant motor cortex of controls, EMG data collected from contralateral FDI. CS was adjusted to produce 1 mV MEP, TS was adjusted to produce 1 mV MEP. ISI: 50-200ms. Data collected both at rest and during voluntary muscle activation.<br>Major outcome: LICI alteration in psychogenic and organic dystonia	LICI at rest was reduced in organic dystonia relative to HC across all ISIs (p=0.009).<br>Psychogenic dystonia patients' LICI did not significantly differ from HC.<br>Difference between organic and psychogenic dystonia patients was significant only at 50 ms ISI at rest (p=0.047).<br>During voluntary muscle activation there was no significant LICI difference across groups.

Cantello, et al. 2007	Design: Cross-sectional<br>Participants: Total of 30 participants: 18 PD patients, 12 HC<br>*Age (yrs):* PD: 63.3 ± 12.7, HC: 59.2 ± 7.7<br>*Sex:* PD: 10M/8F, HC: 7M/5F<br>Measures: TMS administered motor cortex, EMG data collected from active FDI muscle. CS set to 150% AMT, TS set to 150% AMT. ISI: 50, 100, 150, 200, 250, 300 ms. PD patients were tested both on and off medication.<br>Major outcome: LICI alteration in early, drug naive idiopathic PD patients	Enhanced LICI in PD patients relative to HC was identified in both more affected and less affected sides at ISI of 250 ms ($p<0.05$).<br>There was no significant difference between groups at other interstimulus intervals.<br>LICI changes showed a trend to correlate with Parkinsonism signs severity ($p=0.1$).
Fierro, et al. 2008	Design: Clinical trial<br>Participants: Total of 22 participants: 14 PD patients, 8 HC<br>*Age (yrs):* PD: 69.9 ± 8.3, HC: 66.7 ± 9.8<br>*Sex:* PD: 8M/6F, HC: 5M/3F<br>Measures: TMS administered to left motor cortex, EMG data collected from contralateral APB. CS was set to 120% RMT, TS was set to 120% RMT. ISI: 80 ms. PD patients were tested both on and off medication.<br>Major outcome: Effect of high frequency rTMS over LICI in PD patients	LICI was significantly reduced in off medication PD patients relative to on medication PD patients ($p= 0.005$) and HC ($p< 0.016$).<br>LICI significantly increased following rTMS in off medication PD patients ($p< 0.01$).<br>LICI in PD patients on medication did not significantly change after rTMS.
Chu, et al. 2009	Design: Cross-sectional<br>Participants: Total of 20 participants: 11 PD patients, 9 HC<br>*Age:* PD: 65.5 ± 8.8, HC: 61.3 ± 4.3<br>*Sex:* PD: 10M/1F, HC: 2M/7F<br>Measures: TMS administered to motor cortex, EMG data collected from contralateral FDI. CS was set to produce MEP of 1.0 mV, TS was set to produce MEP of 1.0 mV. ISI: 100, 150 ms. PD patients were tested both on and off medication.<br>Major outcome: LICI alteration in PD patients.<br>Additional outcome: Effect of LICI on SICI in PD patients	LICI was reduced in PD patients both on and off medication at 100 and 150 ms interstimulus intervals relative to HC ($p=0.035$)<br>LICI led to a greater reduction of SICI in HC relative to PD patients on ($p=0.0098$) and off ($p=0.001$) medication states.
Meunier, et al. 2012	Design: Cross-sectional<br>Participants: Total of 36 participants: 17 dystonia patients, 19 HC<br>*Age (yrs):* Dystonia: 51.3 ± 2.2, HC: 46.3 ± 3.1<br>*Sex:* Dystonia: 15M/2F, HC: not listed<br>Measures: TMS administered to affected motor cortex of patients and left motor cortex of controls, EMG data collected from FPB and ADM. CS was set to 110% RMT, TS was set to 120% RMT. ISI: 90 ms.<br>Major outcome: Influence of paired associative stimulation and motor learning on LICI in dystonia patients	LICI was not significantly different across groups at baseline.<br>LICI decreased following learning of simple motor tasks ($p<0.01$) and PAS intervention in HC ($p<0.01$).<br>There was no significant change in LICI after learning of the simple motor task in dystonia patients.<br>PAS did not lead to any significant change of LICI in the patient group.

Barbin, et al. 2013	Design:Clinical trial<br>Participants: Total of 30 participants: 20 PD patients 10 with and 10 without dyskinesia, 10 HC<br>*Age (yrs):* PD dyskinetic: 54.6±2.5, PD non-dyskinetic: 60.9 ±2.6, HC: 54.4± 1.9<br>*Sex:* PD dyskinetic: 3F/7M, PD non-dyskinetic: 4F/6M, HC: not listed<br>Measures: TMS administered to motor cortex, EMG data collected from contralateral ADM. CS was set to 120, 180% RMT, TS was set to 120, 180% RMT. ISI: 100 ms. PD patients were tested both on and off medication.<br>Major outcome: LICI alteration in dyskinetic and non-dyskinetic PD patients.<br>Additional outcome: Effect of Levodopa treatment on LICI in dyskinetic and non-dyskinetic PD patients.	LICI was reduced in dyskinetic PD patients relative to HC ($p< 0.05$) at off-medication state and the difference was still present following levodopa administration ($p< 0.05$).<br>LICI difference in between non-dyskinetic patients and HC was not significant at both on and off medication states.<br>LICI was significantly reduced in dyskinetic patients relative to non-dyskinetic only at on-medication state ($p< 0.05$).<br>Increasing CS stimulus to 180% RMT led to reduction of LICI in HC and shift to facilitation ($p<0.01$).<br>LICI reduction with increased CS intensity was prominent at on-medication state for both dyskinetic ($p<0.01$) and non-dyskinetic patients ($p<0.01$) but it was non-significant at the off-medication state.
Lu, et al. 2016	Design: Cross-sectional<br>Participants: Total of 36 participants: 12 PD patients, 12 essential tremor patients, 12 HC<br>*Age (yrs):* PD: 66.1 ± 8.8, ET: 65.2 ± 8.9, HC: 68.9 ± 8.8<br>*Sex:* PD: 10M/2F, ET: 6M/6F, HC: 6M/6F<br>Measures: TMS administered to left motor cortex, EMG data collected from left APB. CS was set to produce MEP of 1.0 mV, TS was set to produce MEP of 1.0 mV. ISI: 100 ms. All participants were tested before and after PAS.<br>Major outcome: Effect of PAS induced long term potentiation like (LTP) like plasticity on LICI in PD and ET patients.	PAS caused a reduction in LICI irrespective of groups ($p<.0.01$).
Philpott, et al. 2016	Design: Cross-sectional<br>Participants: Total of 45 participants: 28 HD patients: 16 premanifest HD + 12 symptomatic HD, 17 HC<br>*Age (yrs):* Pre-HD: 42 ± 8, Symp-HD: 55 ± 9 HC: 42 ± 12<br>*Sex:* Pre-HD: 6M/10F, Symp-HD: 7M/5F HC: 6M/11F<br>Measures: TMS administered to left primary motor cortex, EMG data collected from right APB. CS was set to 120% RMT, TS was set to 120%. ISI: 100 ms. Clinical severity was measured with Unified Huntington's disease rating scale. Gene testing was performed for CAG repeats. Behavioral symptoms were assessed with Frontal Systems Behavior Scale (FrSBe).<br>Major outcome: LICI alteration in premanifest and symptomatic HD patients.	LICI was reduced in pre-HD and symp-HD participants relative to controls ($p=0.02$).<br>LICI impairment was correlated with the number of CAG repeats in pre-HD patients ($p=0.01$).<br>LICI did not show significant correlation with neurocognitive measures.<br>Association between FrSBe scores and LICI was not significant.

	Additional outcome: Correlation of LICI impairment with clinical severity, neurocognitive performance and psychiatric symptoms.	
Latorre, et al. 2021	Design: Cross-sectional<br>Participants: Total of 37 participants: 10 with dystonic syndrome, 7 with primary writing tremor, 10 with essential tremor, 10 HC<br>*Age (yrs):* DTS: 54.5 ± 9.6, PWT: 58.0 ± 17.1, ET: 55.5 ± 11.4, HC: 55.2 ± 10.7<br>*Sex:* DTS: 3F/7M, PWT: 7M, ET:7F/3M, HC: 3F/7M<br>Measures: TMS administered to primary motor cortex, EMG data controlateral FDI. CS was set to 120% RMT, TS was set to produce MEP of 1.0 mV. ISI: 100 ms. TMS measures were recorded at baseline and at the end of paired associative plasticity protocol.<br>Major outcome: LICI alteration in primary writing tremor, essential tremor and dystonic syndrome.	Baseline LICI was not significantly different across groups. Paired associative plasticity protocol led to significant LICI decrease in HC ($p<0.001$) and ET ($p<0.001$) but LICI was unchanged in PWT and DTS patients.

PD: Parkinson's disease
HC: Healthy controls
TMS: Transcranial magnetic stimulation
EMG: Electromyography
FDI: First dorsal intraosseous
CS: Conditioning stimulus
RMT: Resting motor threshold
TS: Test stimulus
ISI: Interstimulus interval
LICI: Long-interval intracortical inhibition

ADM: Abductor digiti minimi
ET: Essential tremor
HD: Huntington's disease
MEP: Mean evoked potential
GPi: Globus pallidus interna
APB: Abductor pollicis brevis
STN: Subthalamic nucleus stimulation
AMT: Active motor threshold
FPB: Flexor pollicis brevis
PAS: Paired associative stimulation
PWT: Primary writing tremor
DTS: Dystonic Syndrome

6kx

Supplementary Table 10 LICI in Multiple Sclerosis patients

Reference	Study Description	Findings
Mori, et al. 2013	Design: Cross-sectional<br>Participants: 89 Multiple sclerosis patients (74 of the had results available)<br>*Age (yrs):* 37.7 ± 9.87<br>*Sex:* 57F/ 17 M<br>Measures: TMS administered to right motor cortex, EMG data collected from left FDI. CS was set to produce 120% RMT, TS adjusted to produce 1 mV MEP. ISI: 100 ms. Disability was measured with expanded disability status scale (EDSS).<br>Major outcome: Correlation of LICI with level of disability in multiple sclerosis patients.	There was no significant correlation between LICI and EDSS scores.
Nantes, et al. 2016	Design: Cross-sectional<br>Participants: Total of 54 participants: 36 MS patients:22 relapsing-remitting+14 Progressive MS, 18 HC<br>*Age (yrs):* Relapsing remitting MS: 44 ± 12, Progressive MS: 60 ± 13, HC: 45 ± 14<br>*Sex:* Relapsing remitting MS: 15F/7M, Progressive MS: 10F/2M, HC: 13F/5M<br>Measures: TMS administered left motor cortex, EMG data collected from right FDI. CS adjusted to produce 100% RMT, TS adjusted to produce 100% RMT. ISI: 100 ms. Cortical volume and cortical magnetization transfer ratio (MTR) assessed with MRI.<br>Major outcome: Association between LICI and cortical damage in MS patients.	LICI did not differ across groups significantly.<br>LICI was not significantly correlated with cortical volume or MTR in MS patients or controls.
Squintani, et al. 2016	Design: Clinical trial<br>Participants: Total of 38 participants: 19 MS patients, 19 HC<br>*Age (yrs):* 56.1 ± 8.9, HC: not listed<br>*Sex:* 5M/14F, HC: not listed<br>Measures: TMS administered dominant motor cortex, EMG data collected from APB. CS was adjusted to produce 120% RMT, TS was adjusted to produce 120% RMT. ISI: 100 ms. Patients were assessed at baseline and 4 weeks after starting Sativex (9-tetrahydrocannabinol and cannabidiol) treatment.<br>Major outcome: Change in LICI following Sativex treatment in MS patients	LICI was reduced in patients relative to healthy controls at baseline ($p< 0.05$).<br>LICI showed significant enhancement following Sativex treatment ($p< 0.05$).<br>LICI in patients did not significantly differ from healthy controls after treatment.

HC: Healthy controls
TMS: Transcranial magnetic stimulation
EMG: Electromyography
FDI: First dorsal interosseous
CS: Conditioning stimulus
RMT: Resting motor threshold
TS: Test stimulus
ISI: Interstimulus interval
LICI: Long-interval intracortical inhibition
MS: Multiple sclerosis
APB: Abductor Pollicis Brevis

Supplementary Table 11 LICI in Stroke patients

Reference	Study Description	Findings
Swayne, et al. 2008	Design: Cohort<br>Participants: Total of 20 participants: 10 post-stroke patients, 10 HC<br>*Age (yrs):* Stroke patients: 54 yrs, HC: not listed<br>*Sex:* Stroke patients: 4F/6M, HC: not listed<br>Measures: TMS administered to affected and unaffected motor cortex, EMG data collected from FDI. Both CS and TS set to lowest stimulus intensity resulting in a MEP of stable size. ISI: 100 ms. Assessments conducted weekly until 1 month and repeated at 3 and 6 months. Clinical performance was measured with the action research arm test (ARAT) and nine-hole peg test (NHPT).<br>Major outcome: LICI changes following stroke at multiple time points	There was no significant effect of time on LICI.<br>LICI was significantly reduced in the patient group relative to HC (p=0.029) in the affected side but not in the unaffected site.<br>In the affected hemisphere LICI deficit was significantly correlated with poorer clinical scores in the acute period (p=0.0024) and at 3 months (p=0.0387) but no longer correlated at 6 months.<br>Same applied for the unaffected side at only 3 months.
Kuppuswamy, et al. 2014	Design: Cross-sectional<br>Participants: 70 post-stroke patients<br>*Age (yrs):* 60.36 ± 12.4<br>*Sex:* 20F/50M<br>Measures: TMS administered to affected side motor cortex, EMG data collected from FDI. CS adjusted to produce 1 mV MEP, TS adjusted to produce 1 mV MEP. ISI: 100 ms. Fatigue was assessed with Fatigue Severity Scale and Neurological Fatigue Index.<br>Major outcome: Association between LICI and post stroke fatigue	LICI was not correlated with measures of fatigue in post stroke patients.
Schambra, et al. 2015	Design: Cross-sectional<br>Participants: Total of 62 participants: 20 subacute stroke subjects, 21 chronic stroke subjects, 21 HC<br>*Age (yrs):* Subacute stroke: 72.2 ± 12.7, Chronic stroke: 62.0 ± 9.2, HC: 64.7 ± 10.1<br>*Sex:* Subacute stroke: 11M/9F, Chronic stroke: 15M/6F, HC: 10M/11F<br>Measures: TMS administered to bilateral motor cortex, EMG data collected from FDI. CS adjusted to produce 1 mV MEP, TS adjusted to produce 1 mV MEP. ISI: 100 ms. Participants were studied in four sessions, on two consecutive days in morning and afternoon. Standard error of measurement assessed with Smallest detectable change (SDC) and measurement property of reliability assessed with Intraclass Correlation Coefficient (ICC).<br>Major outcome: Reliability of LICI in subacute and chronic stroke patients and healthy subjects	There was no significant LICI difference between patient groups and healthy subjects.<br>LICI was indicated as an unreliable marker for detecting individual change.<br>lnLICI was found to be a reliable marker for discriminating between subjects (ICC> 0.70).

Schambra, et al. 2016	Design: Double-blind placebo-controlled randomized cross-over<br>Participants: 18 post-stroke patients<br>*Age (yrs):* 63.9 ± 10.7<br>*Sex:* 13M/5F<br>Measures: TMS administered to both lesioned and non-lesioned motor cortex, EMG data collected from FDI. CS adjusted to produce 0.5-1.0 mV MEP, TS adjusted to produce 0.5-1.0 mV MEP. ISI: 100 ms. Subjects were randomized to receive either theophylline or placebo on the first day and opposite on the second day at least one week apart. Measurements were taken in the morning immediately after capsule intake and 5 hours later in the afternoon session on each day.<br>Major outcome: Effect of theophylline on LICI in chronic stroke patient	Change of LICI from morning to afternoon session was not significantly different across placebo and theophylline groups for both hemispheres.
Mooney, et al. 2019	Design: Cross-sectional study<br>Participants: Total of 28 participants: 12 chronic stroke patients, 16 HC<br>*Age (yrs):* Chronic stroke patients: 71, HC: 70<br>*Sex:* Chronic stroke patients: 11M/1F, HC: 9M/7F<br>Measures: TMS administered to both ipsilesional and contralesional motor cortex, EMG data collected from FDI. CS adjusted to produce 130% RMT, TS adjusted in 1% maximum stimulator output steps until threshold tracking and target MEP was achieved. ISI: 150, 175, 200, 225 and 250 ms. Magnetic resonance spectroscopy was used to assess GABA levels.<br>Major outcome: LICI alteration and GABA concentration in chronic stroke patients	LICI was enhanced in chronic stroke patients relative to HC in ipsilesional motor cortex ($p<0.001$).<br>There was no significant correlation between LICI and metabolite concentrations in ipsilesional, contralesional cortex or healthy controls.
Mooney, et al. 2020	Design: Cohort<br>Participants: Total of 22 participants: 10 chronic stroke patients, 12 HC<br>*Age (yrs):* Chronic stroke patients: 77, HC: 77<br>*Sex:* Chronic stroke patients: 8M/2F, HC: 6M/6F<br>Measures: TMS administered to ipsilesional motor cortex in patients and dominant motor cortex in HC, EMG data collected from ECR. CS adjusted to produce 120% RMT, TS adjusted in 1% maximum stimulator output steps until threshold hunting target was achieved. ISI: 100, 150 ms. LICI was assessed immediately before and after skill acquisition session, 7 days and 24 days post training.<br>Major outcome: Modulation of LICI following in ipsilesional cortex during motor skill learning	Ipsilesional motor cortex LICI was enhanced in stroke group relative to controls at 150 ms interstimulus interval ($p=0.030$).<br>LICI did not change significantly after training in both HC and patient groups.

HC: Healthy controls
TMS: Transcranial magnetic stimulation
EMG: Electromyography
CS: Conditioning stimulus
MEP: Mean evoked potential
TS: Test stimulus
ISI: Interstimulus interval
LICI: Long-interval intracortical inhibition
RMT: Resting motor threshold
ECR: Extensor carpi radialis

Supplementary Table 12 LICI in TBI patients

Reference	Study Description	Findings
Tremblay, et al. 2011	Design: Cross-sectional<br>Participants: Total of 26 participants, 12 concussed, 14 non-concussed football players.<br>*Age (yrs):* Concussed: not given, Non-concussed: 22.36 ± 1.69, Sex: not given<br>*Number of concussions:* ≥2, *Time since last Concussion*: >1 year<br>Measures: TMS administered to left motor cortex, EMG data collected from right APB. CS was set to produce MEP of 0.2-1.5 mV, TS was set to produce MEP of 0.2-1.5 mV ISI: 100 ms.<br>Major outcome: Cortical inhibitory dysfunction, measuring with LICI, in concussed athletes	Concussed athletes demonstrated significantly enhanced LICI relative to the non-concussed control group. (p=0.05)
De Beaumont, et al. 2011	Design: Case-control<br>Participants: Total of 36 participants, 21 concussed, 15 non-concussed football players.<br>*Age (yrs):* 22.3 ± 3.45 *Sex:* M<br>*Number of concussions:* 1-5, *Time since last Concussion*: 9-34 months<br>Measures: TMS administered to left motor cortex, EMG data collected from right FDI. CS was set to 120% RMT, TS was set to produce MEP of 0.2-1.5 mV ISI: 100 ms. A platform was used to measure center of pressure (COP) displacement and COP oscillation regularity to evaluate postural stability.<br>Major outcome: Cortical inhibition, measuring with LICI and CSP, in concussed athletes	Football players with concussion history showed enhanced LICI compared to non-concussed athletes. (p< 0.03).<br>Number of concussions was positively correlated with cortical inhibition. (p<0.05)<br>Enhanced LICI was correlated with longer CSP (p<0.05).<br>There was no significant association between COP oscillation regularity and LICI.
De Beaumont, et al. 2012	Design: Cross-sectional<br>Participants: Total of 32 participants, 13 concussed, 19 non-concussed football players.<br>*Age (yrs):* Concussed: 23.41 ± 2.21, Non-concussed: 22.78 ± 1.88, Sex: M<br>*Number of concussions:* 2-7, *Time since last Concussion*: > 9 months<br>Measures: TMS administered to left motor cortex, EMG data collected from right APB. CS was set to produce MEP of 1.0 mV, TS was set to produce MEP 1.0 mV. ISI: 100 ms. Paired associative stimulation used to induce LTP/LTD- like effect on motor cortex, while learning serial reaction time task (SRTT)<br>Major outcome: Effect of LICI on LTP/LTD like synaptic plasticity in concussed players	Baseline LICI was enhanced in the concussed group. (p=0.037)<br>Enhancement in LICI in concussed athletes was correlated to the degree which PAS induced LTP/LTD-like synaptic plasticity (p value not indicated).

Pearce, et al. 2014	Design: Cross-sectional<br>Participants: Total of 60 participants, 40 concussed retired Australian football players.: 20 elite + 20 amateur, 20 HC<br>*Age (yrs):* Elite: 49.74 ± 5.67, Amateur: 48.46 ± 6.86, HC: 47.56 ± 6.85. Sex: M<br>*Number of concussions:* 3.2±2.4, *Time since last Concussion*: 22.1±6.7 yr<br>Measures: TMS administered to left motor cortex, EMG data collected from right FDI. CS was set to 125% RMT, TS was set to 125% RMT. ISI: 100 ms. Neuropsychological tests measured fine motor finger dexterity (O'connor test), visuomotor reaction time, spatial working memory and associative learning.<br>Major outcome: Neurobiological deficits in concussed players and its correlation with LICI	Football players had reduced LICI relative to healthy controls. (p> 0.001)<br>No significant LICI difference between elite and amateur level players detected.<br>Reduction in LICI was correlated with poorer performance in fine motor dexterity. (p=0.049)
Tremblay, et al. 2014	Design: Cross-sectional<br>Participants: Total of 30 participants, 16 concussed, 14 non-concussed football players.<br>*Age (yrs):* Concussed: 22.00 ± 1.09, Non-concussed: 22.03 ± 1.08, Sex: M<br>*Number of concussions:* 1-4, *Time since last Concussion*: 10-96 months<br>Measures: TMS administered to left motor cortex, EMG data collected from right FDI. CS was set to produce MEP of 1.0 mV, TS was set to produce MEP 1.0 mV. ISI: 100 ms. GABA and Glx levels measured with Proton magnetic resonance spectroscopy (^{1}H-MRS).<br>Major outcome: Metabolic disruption, quantified by H-MRS measured GABA levels, and cortical inhibition measured by LICI following concussion	There was no significant difference in LICI between two groups.<br>GABA levels measured with H-MRS demonstrated significant positive correlation with LICI (0.001) in the concussed group but not control group.<br>No significant correlation between Glx and LICI.
Powers, et al. 2014	Design: Cross-sectional<br>Participants: Total of 16 participants, 8 concussed, 8 non-concussed football players.<br>*Age (yrs):* Concussed: 20.16 ±1.19, Non-concussed: 20.28 ±1.47, Sex: M<br>*Number of concussions:* 0-2, *Time since last Concussion*: 1-4 weeks<br>Measures: TMS administered to left motor cortex, EMG data collected from right FDI. CS was set to produce MEP of 1.0 mV, TS was set to produce MEP 1.0 mV. ISI: 100 ms.<br>Major outcome: LICI in concussed athletes during acute asymptomatic phase following concussion	There was no significant effect of concussion on LICI levels.

Lewis, et al.<br>2017	<u>Design:</u> Cross-sectional<br><u>Participants:</u> Total of 73 participants, 23 retired elite rugby players, 28 retired community level rugby players, 22 non-contact sport control<br>*Age (yrs):* Elite palyers: 43 ±7, Community level players: 45 ± 8, Control: 44 ± 9 Sex: M<br>*Number of concussions:* ≥ 1, *Time since last Concussion*: > 5 years<br><u>Measures:</u> TMS administered to dominant motor cortex, EMG data collected from right FDI. CS was set to produce MEP of 120% RMT, TS was set to produce MEP 1.0 mV. ISI: 99 ms.<br><u>Major outcome:</u> LICI in retired competitive rugby players in comparison to retired non-contact sport athletes.	Elite rugby players had a significantly enhanced LICI relative to non-contact sport controls (p=0.005).<br>There was no significant LICI difference across community level players and controls.
Seeger, et al. 2017	<u>Design:</u> Cross sectional-controlled cohort<br><u>Participants:</u> Total of 90 participants, 62 children with mTBI: 35 symptomatic+27 asymptomatic, 28 HC<br>*Age (yrs):* Symptomatic TBI: 14.06 ± 2.55, Asymptomatic TBI: 14.13 ± 2.35, HC: 14.31 ± 3.14<br>Sex: Symptomatic TBI: 19F/16M, Asymptomatic TBI: 14F/13M HC: 15F/13M<br>*Number of previous concussions:* 0-2, *Time since TBI*: 4 weeks<br><u>Measures:</u> TMS administered to dominant motor cortex, EMG data collected from contralateral FDI. CS was set to produce MEP of 1000 μV RMT, TS was set to produce MEP 1000 μV RMT. ISI: 100 ms.<br><u>Major outcome:</u> Alteration in LICI following mTBI in children	LICI was reduced in the symptomatic mTBI group relative to HC (p=0.027).<br>LICI deficit was correlated with post-concussion symptom severity (p=0.012).<br>LICI was affected by sex, female demonstrating more pronounced inhibition (p=0.016).
Pearce, et al. 2018	<u>Design:</u> Cross-sectional<br><u>Participants:</u> Total of 50 participants, 25 retired concussed rugby players, 25 HC<br>*Age (yrs):* Concussed: 48.4, Non-concussed: 48.8 Sex: M<br>*Number of concussions:* 4-11, *Time since last Concussion*: 15-21 years<br><u>Measures:</u> TMS administered to motor cortex, EMG data collected from contralateral FDI. CS was set to 125% RMT, TS was set to 125% RMT. ISI: 100 ms. Neuropsychological tests measured fine motor finger dexterity (O'connor test), visuomotor reaction time, spatial working memory and associative learning.<br><u>Major outcome:</u> LICI in concussed rugby players and its correlation with cognitive changes	Reduced LICI was demonstrated in concussed rugby players versus healthy controls (p= 0.03).<br>Fine motor (O'Connor test) performance was negatively correlated with LICI (p < 0.01).<br>No significant correlation with reaction and movement time.

Pearce, et al. 2019	Design: Cross-sectional<br>Participants: Total of 60 participants, 20 with PCS, 20 recovered concussion, 20 HC.<br>*Age (yrs):* PCS: 36.2 ± 14.0, Recovered: 33.8 ± 6.6, HC: 37.7 ± 8.0<br>Sex: 4F/16M, Recovered: 2F/18M, HC: 3F/17M<br>*Number of concussions:* PCS: 1-7, Recovered: 2-7 *Time since last Concussion*: PCS: 8-23 months, Recovered: 6-19 months<br>Measures: TMS administered to motor cortex, EMG data collected from contralateral FDI. CS set to 130% RMT, TS set to 130% RMT. ISI: 100 ms. Fatigue measured with self-report questionnaires.<br>Major outcome: Cortical inhibition, LICI, in those with ongoing PCS<br>Additional outcome: Correlation of LICI with fatigue and somatosensory measures	LICI was significantly enhanced in PCS group relative to recovered subjects and control group ($p<0.001$).<br>LICI was not significantly different between recovered concussion patients and control groups.<br>Worsening of fatigue ($p<0.001$) and amplitude ($p=0.02$) discrimination was correlated with increase in cortical inhibition.<br>LICI was not significantly associated with reaction time and variability.
Opie, et al. 2019	Design: Cross-sectional<br>Participants: Total of 32 participants, 17 with mTBI, 15 HC<br>*Age (yrs)*: mTBI: 27.4 ± 2.5, HC: 25.3 ± 2.0<br>Sex: mTBI: 1F/16M, HC: 2F/13M *Time since mTBI*: 85-171 days<br>Measures: TMS administered to left motor cortex, EMG data collected from right FDI. CS was set to produce MEP of 1.0 mV, TS was set to produce MEP 1.0 mV. ISI: 100 ms. EEG used to measure P30, N45, N100, P200.<br>Major outcome: LICI alteration, using TMS-EEG measures, following mTBI	LICI measured with EMG was enhanced in mTBI group. ($p < 0.0001$)<br>LICI induced inhibition of TMS-evoked EEG potentials were not significantly different between groups.
King, et al. 2019	Design: Prospective, longitudinal, controlled cohort<br>Participants: Total of 133 participants, 78 with PPCS, 29 asymptomatic mTBI, 26 HC. 2nd session completed by 9 asymptomatic, 58 symptomatic patients.<br>*Age (yrs)*: PPCS: 15.2 ± 2, Asymptomatic TBI: 14.2 ± 2, HC: 14.6 ± 3<br>Sex: PPCS: 47F/31M, Asymptomatic TBI: 14F/15M, HC: 14F/12M<br>Participants in the TBI group were assessed 1 month and 2 months post injury. Control group seen 1-month postinjury.<br>Measures: TMS administered to left motor cortex, EMG data collected from right FDI. CS set to 120% RMT, TS set to 120% RMT. ISI: 100 ms.<br>Major outcome: Association between LICI and post concussive symptoms over time in children with mTBI	Groups did not differ from each other significantly based on LICI at 1-month post injury. (ns)<br>LICI did not change significantly over time.<br>LICI was not significantly different between groups at 2 months post injury.

HC: Healthy controls
TMS: Transcranial magnetic stimulation
EMG: Electromyography
APB: Abductor pollicis brevis
MEP: Mean evoked potential
CS: Conditioning stimulus
TS: Test stimulus
ISI: Interstimulus interval
LICI: Long-interval intracortical inhibition
FDI: First dorsal interosseous
RMT: Resting motor threshold
CSP: cortical silent period
LTP: Long-term potentiation
LTD: Long-term depression
PAS: Paired associative stimulation
mTBI Mild traumatic brain injury
PCS: post-concussion syndrome
EEG: Electroencephalogram
PPCS: Persistent post-concussive symptoms
